# Discovery and Mechanistic Investigation of Piperazinone Phenylalanine Derivatives with Terminal Indole or Benzene Ring as Novel HIV-1 Capsid Modulators

**DOI:** 10.3390/molecules27238415

**Published:** 2022-12-01

**Authors:** Shujing Xu, Lin Sun, Waleed A. Zalloum, Tianguang Huang, Xujie Zhang, Dang Ding, Xiaoyu Shao, Xiangyi Jiang, Fabao Zhao, Simon Cocklin, Erik De Clercq, Christophe Pannecouque, Alexej Dick, Xinyong Liu, Peng Zhan

**Affiliations:** 1 Key Laboratory of Chemical Biology (Ministry of Education), Department of Medicinal Chemistry, School of Pharmaceutical Sciences, Shandong University, 44 West Culture Road, Jinan 250012, China; 2 Department of Pharmacy, Qilu Hospital of Shandong University, Jinan 250012, China; 3 Department of Pharmacy, Faculty of Health Science, American University of Madaba, P.O. Box 2882, Amman 11821, Jordan; 4 Specifica Inc., The Santa Fe Railyard, 1607 Alcaldesa Street, Santa Fe, NM 87501, USA; 5 Laboratory of Virology and Chemotherapy, Rega Institute for Medical Research, K.U. Leuven, Herestraat 49 Postbus 1043 (09.A097), B-3000 Leuven, Belgium; 6 Department of Biochemistry & Molecular Biology, Drexel University College of Medicine, Philadelphia, PA 19102, USA

**Keywords:** HIV-1, PF74, capsid modulators, target identification, mechanistic investigation

## Abstract

HIV-1 capsid (CA) performs multiple roles in the viral life cycle and is a promising target for antiviral development. In this work, we describe the design, synthesis, assessment of antiviral activity, and mechanistic investigation of 20 piperazinone phenylalanine derivatives with a terminal indole or benzene ring. Among them, **F_2_-7f** exhibited moderate anti-HIV-1 activity with an EC_50_ value of 5.89 μM, which was slightly weaker than the lead compound **PF74** (EC_50_ = 0.75 μM). Interestingly, several compounds showed a preference for HIV-2 inhibitory activity, represented by **7f** with an HIV-2 EC_50_ value of 4.52 μM and nearly 5-fold increased potency over anti-HIV-1 (EC_50_ = 21.81 μM), equivalent to **PF74** (EC_50_ = 4.16 μM). Furthermore, **F_2_-7f** preferred to bind to the CA hexamer rather than to the monomer, similar to **PF74**, according to surface plasmon resonance results. Molecular dynamics simulation indicated that **F_2_-7f** and **PF74** bound at the same site. Additionally, we computationally analyzed the ADMET properties for **7f** and **F_2_-7f**. Based on this analysis, **7f** and **F_2_-7f** were predicted to have improved drug-like properties and metabolic stability over **PF74**, and no toxicities were predicted based on the chemotype of **7f** and **F_2_-7f**. Finally, the experimental metabolic stability results of **F_2_-7f** in human liver microsomes and human plasma moderately correlated with our computational prediction. Our findings show that **F_2_-7f** is a promising small molecule targeting the HIV-1 CA protein with considerable development potential.

## 1. Introduction

Acquired immunodeficiency syndrome (AIDS) is a series of syndromes characterized by T cell immune deficiency caused by human immunodeficiency virus (HIV) infection [1]. Since the first case of AIDS was reported in 1981, approximately 40 million people have died of AIDS worldwide [2]. The rapid spread of AIDS and its high mortality rate threaten human health and social development. HIV contains two species: HIV-1 is the predominant pathogenic pathogen, but the danger of HIV-2 infection is increasing and has been discovered globally [3,4]. Current combination antiretroviral therapy (cART) and, in light of frequent mutations within the retrovirus, available antivirals can delay disease progression and suppress viral load in patients; however, a cure is currently not possible due to latent viral pools induced by cART [5,6,7]. Therefore, focusing on potential new targets and developing novel HIV inhibitors with new mechanisms of action are of great significance for developing antiviral drugs.

HIV-1 capsid (CA) is a structural protein encoded within the HIV-1 Gag gene [8]. The capsid protein forms a higher order structure that encapsulates and protects the core proteins, viral genome, and various enzymes essential for viral replication [9,10]. Because of its important role in the early and late stages of viral replication, CA has become a promising new target for anti-HIV-1 drug design [11,12]. The mature HIV-1 CA is a conical ‘fullerene-like’ (cone) lattice shell comprising approximately 1500 CA monomers, which are arranged in approximately 250 hexamers and 12 pentamers [13,14]. The mature CA monomer consists of an N-terminal domain (NTD), a C-terminal domain (CTD), and a flexible linker in the middle [15]. CA cone stability highly depends on intermolecular contacts such as NTD-NTD, NTD-CTD, and CTD-CTD between monomers of the hexamer or pentamer [16]. Notably, the NTD-CTD interprotomer pocket is the binding site of host factor cleavage and polyadenylation specificity factor 6 (CPSF6), nucleoporin 153 (NUP153), and Sec24C [17,18,19]. Interference with interactions between CA and host factors can affect HIV-1 replication. Furthermore, the NTD-CTD interface is also critical for CA assembly, which can affect the intrinsic flexibility of CA and subsequently destroy the integrity of virus particles [20,21]. Therefore, the NTD-CTD interface is an effective site for developing novel and potent CA modulators.

**Figure 1:**
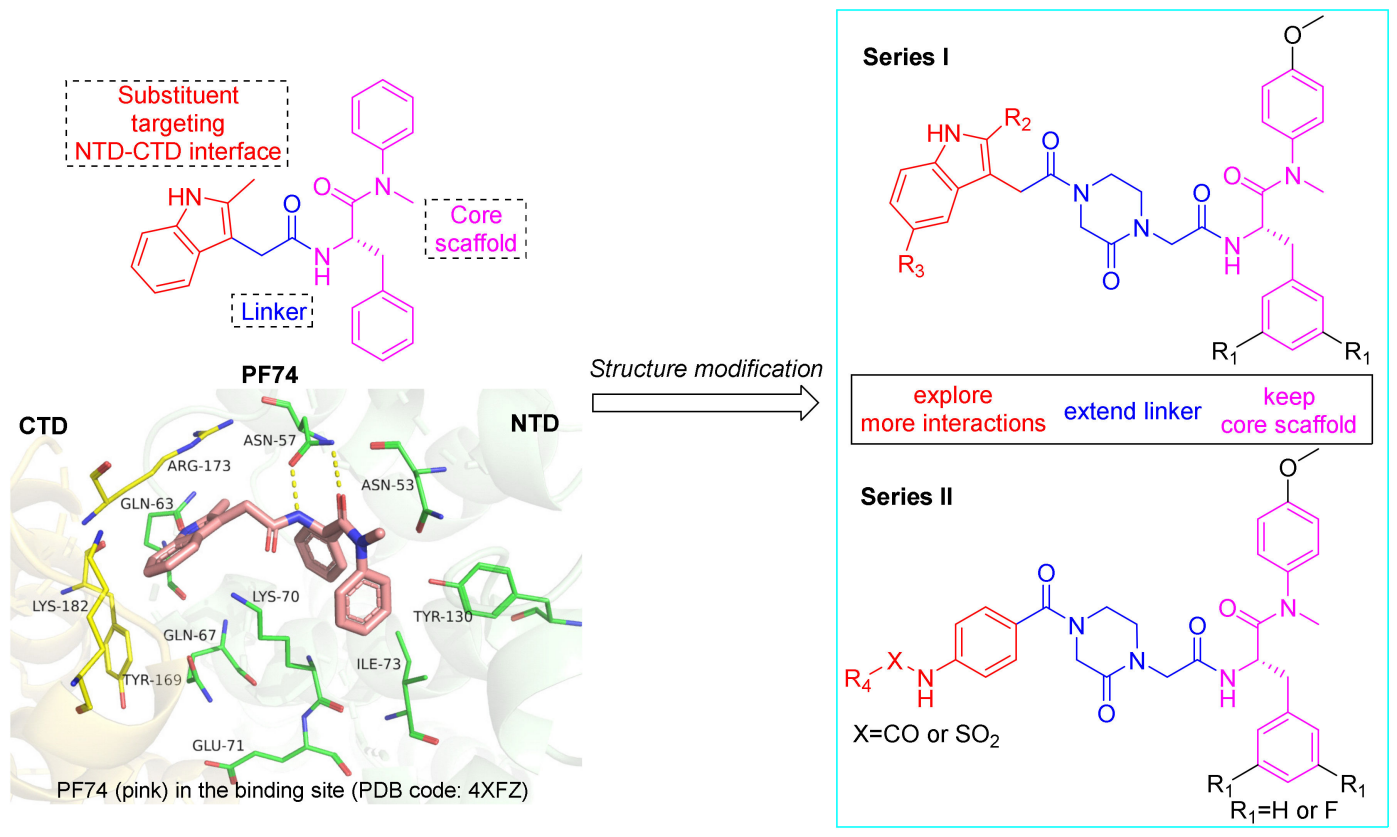
Design principle of piperazinone phenylalanine derivatives with terminal indole or benzene ring as HIV-1 CA modulators. The figure was generated in PyMOL (www.pymol.org, accessed on 20 October 2022).

**PF74**, a small molecule modulator targeting the NTD-CTD interface of the adjacent subunit of CA hexamers, can inhibit viral replication through multiple effects and has received extensive attention [22,23]. **PF74** comprises a phenylalanine core, an indole substituent, and a linker between them. Structural studies have shown that the phenylalanine core of **PF74** interacts with Asn53, Asn57, Lys70, Ile73, and Tyr130. Within this interaction, the amide forms multiple, key hydrogen bonds with Asn57. The methyl indole moiety extends to the NTD-CTD interface and interacts with Gln63, Gln67, and Lys70 of the NTD moiety and Arg173 of the CTD moiety (Figure 1) [24,25]. However, the low antiviral activity and inferior metabolic stability of **PF74** make it unsuitable for clinical applications [26]. Extensive decoration of the **PF74** skeleton by Gilead Sciences generated lenacapavir (LEN; GS-6207), which has picomolar anti-HIV-1 activity (EC_50_ = 105 pM), favorable metabolic stability, and is approved by the European Community (EC) [27]. Nevertheless, limited by its complicated structure and labor-intensive synthesis, LEN displays extremely poor water solubility, resulting in mainly injectable use, and drug-resistant strains have also already appeared in clinical trials [28]. Further structural optimization of **PF74** to identify novel CA modulators is needed to address these inadequacies. Crystallographic studies of **PF74** bound within the interprotomer pocket of HIV-1 CA hexamers provide valuable information on binding site flexibility and size, which can guide the design of further modifications of **PF74**, especially at the tolerable indole, as well as the design of novel chemotypes. In this study, we chose to retain the privileged (intolerant) phenylalanine skeleton of **PF74** and introduce a methoxy group at the para position of the aniline ring, which proved to have more favorable antiviral activity [29]. Fewer interactions between the indole ring of **PF74** and CTD might be the reason for its low antiviral activity. Therefore, we extended the linker butane-2-one fragment to a 4-acetyl-1- (2-propionyl) piperazine-2-one fragment rich in hydrogen bond acceptors to maintain interactions with Lys70 and Arg173. Then, different substituted indole groups (series I) and aniline groups (series II) were introduced into the left wing, which were expected to form more interactions with the critical amino acids Gln67, Glu71, Tyr169, and Lys182 at the NTD-CTD interface (Figure 1), thus improving antiviral activity as well as drug-like properties.

Herein, our screening identified 20 piperazinone phenylalanine derivatives with a terminal indole or benzene ring that functioned as HIV-1 CA modulators. The antiviral activities of all newly synthesized compounds were tested using MTT assays, and structure-activity relationships (SARs) were established. We then performed surface plasmon resonance (SPR) and molecular dynamics simulation to investigate the mechanism of action of the representative compounds. Furthermore, the ADMET properties of the representative compounds and **PF74** were computationally predicted. Finally, we experimentally assessed the metabolic stability of **F_2_-7f** in human liver microsomes (HLMs) and human plasma.

## 2. Results and Discussion

### 2.1. Chemistry

**Scheme 1:**
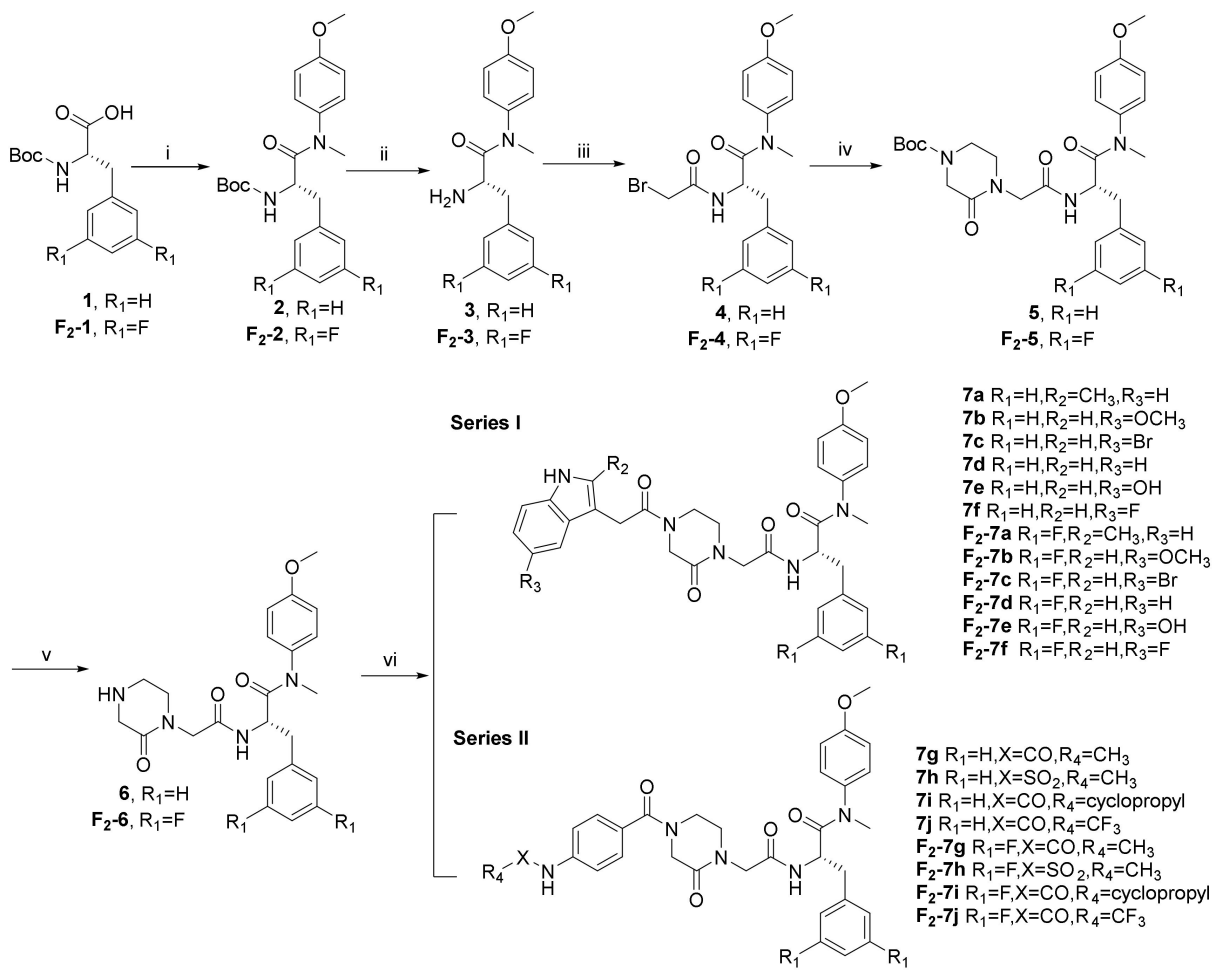
Reagents and conditions: (**i**) 4-Methoxy-*N*-methylaniline, HATU, DIEA, 0 °C to r.t.; (**ii**) TFA, DCM, r.t.; (**iii**) Bromoacetic Acid, HATU, DIEA, 0 °C to r.t.; (**iv**) 1-Boc-3-oxopiperazine, Cs_2_CO_3_, DMF, 45 °C; (**v**) TFA, DCM, r.t.; (**vi**) HATU, DIEA, 0 °C to r.t.

The synthetic route is shown in Scheme 1. *N*-(*tert*-butoxycarbonyl)-*L*-phenylalanine (**1**) and *N*-(*tert*-butoxycarbonyl)-3,5-difluoro-*L*-phenylalanine (**F_2_-1**) were treated with 4-methoxy-*N*-methylaniline and 2-(7-aza-1H-benzotriazole-1-yl)-1,1,3,3-tetramethyluronium hexafluoro (HATU) in the presence of *N*, *N*-diisopropylethyl-amine (DIEA) and dichloromethane (DCM) to obtain **2** and **F_2_-2**, followed by the removal of *tert*-butyloxycarbonyl (Boc) protection using trifluoroacetic acid (TFA), yielding the free amines **3** and **F_2_-3**. The acylation of **3** and **F_2_-3** with bromoacetic acid in DCM resulted in the crucial intermediates **4** and **F_2_-4**. Further treatment of **4** and **F_2_-4** with 1-Boc-3-oxopiperazine via a nucleophilic substitution (SN_2_) reaction afforded the intermediates **5** and **F_2_-5**. The removal of Boc protection of **5** and **F_2_-5** yielded the free amines **6** and **F_2_-6**, which were treated with the corresponding substituted indoleacetic acids and the corresponding substituted benzoic acids in DCM under the action of HATU and DIEA to obtain the target compounds **7** (**a**–**j**) and **F_2_-7** (**a**–**j**).

### 2.2. Antiviral Activity in MT-4 Cells against HIV-1 (III_B_) and HIV-2 (ROD) Replication

All newly synthesized compounds in this study were evaluated for their antiviral activity in an MT-4 cell-based MTT assay containing wild-type (WT) HIV-1 (III_B_) and HIV-2 (ROD), with **PF74** serving as an in-line control. The test compounds’ selectivity index (SI value, CC_50_/EC_50_) was determined by assessing compound toxicity in MT-4 cells using the MTT assay. Table 1 and Table 2 display the results of the analysis.

Most of the compounds in series I showed moderate anti-HIV-1 activity. Unsubstituted compounds and those with an electron-donating substituent of the indole ring led to mid-range efficacy and low toxicity, as in compounds **7d** (EC_50_ = 14.97 ± 0.24 μM), **7a** (EC_50_ = 14.28 ± 0.76 μM) and **7b** (EC_50_ = 14.58 ± 0.39 μM). The efficacy of **F_2_-7f** (EC_50_ = 5.89 ± 2.03 μM) was further improved by introducing two F atoms on the indole ring. Interestingly, this series displayed potent anti-HIV-2 activity, with **7d** (EC_50_ = 4.89 ± 1.39 μM), **7f** (EC_50_ = 4.52 ± 0.87 μM) and **F_2_-7f** (EC_50_ = 5.07 ± 0.63 μM) comparable to **PF74** (EC_50_ = 4.16 ± 2.02 μM). When R_1_ was unsubstituted, the anti-HIV-2 activity was greater than that when R_1_ was substituted with F, such as **7a** (EC_50_ = 7.74 ± 0.79 μM) > **F_2_-7a** (EC_50_ = 19.66 ± 6.59 μM), **7b** (EC_50_ = 16.95 ± 3.58 μM) > **F_2_-7b** (EC_50_ > 15.94 μM), **7d** > **F_2_-7d** (EC_50_ = 17.61 ± 9.14 μM), **7e** (EC_50_ = 186.48 ± 4.42 μM) > **F_2_-7e** (EC_50_ ≥ 49.55 μM), and **7f** > **F_2_-7f**. Remarkably, compounds **7d** and **7f** showed good selectivity for HIV-2.

However, the substitution of R_3_ with Br or OH resulted in a marked decrease in efficacy, such as compounds **7c**, **F_2_-7c**, **7e**, and **F_2_-7e**, indicating that a slight change in the substitutes targeting CTD of CA had a considerable effect on the anti-HIV activity of the compounds.

When R_1_ was substituted with F, the compound toxicities of series I were increased, such as **F_2_-7a** (CC_50_ = 76.85 ± 19.98 μM) > **7a** (CC_50_ = 107.56 ± 3.26 μM), **F_2_-7b** (CC_50_ = 15.94 ± 3.83 μM) > **7b** (CC_50_ = 104.21 ± 3.37 μM), **F_2_-7c** (CC_50_ = 3.15 ± 0.40 μM) > **7c** (CC_50_ = 16.37 ± 3.44 μM), **F_2_-7d** (CC_50_ = 57.84 ± 21.81 μM) > **7d** (CC_50_ = 112.41 ± 3.22 μM), **F_2_-7e** (CC_50_ = 131.51 ± 32.96 μM) > **7e** (CC_50_ > 209.29 μM), and **F_2_-7f** (CC_50_ = 16.36 ± 3.38 μM) > **7f** (CC_50_ = 90.06 ± 15.72 μM). It was worth noting that when R_3_ was Br, the compounds lost their efficacy against HIV-1 and HIV-2, which may have been due to high toxicity.

In series II, the rule of anti-HIV-1 activity for these compounds was that F-substituted R_1_ was superior to unsubstituted R_1_, such as **F_2_-7g** (EC_50_ = 177.40 ± 16.55 μM) > **7g** (EC_50_ = 203.14 ± 10.98 μM), **F_2_-7h** (EC_50_ = 160.53 ± 15.43 μM) > **7h** (EC_50_ = 192.39 ± 3.17 μM), **F_2_-7i** > **7i** (EC_50_ > 167.50 μM), **and F_2_-7j** > **7j** (EC_50_ = 157.50 ± 6.77 μM). When X was SO_2_, the anti-HIV-1 activity was greater than that when X was CO, such as **7h** > **7g** and **F_2_-7h** > **F_2_-7g**. Compounds **F_2_-7i** (EC_50_ = 14.36 ± 1.31 μM) and **F_2_-7j** (EC_50_ = 13.74 ± 0.70 μM) with cyclopropyl and CF_3_ substitution at R_4_ had the best efficacy with low toxicity. For anti-HIV-2 activity, the R_1_ substituent showed the opposite rule with anti-HIV-1 activity; that is, unsubstituted R_1_ was superior to F-substituted R_1_. Specifically, **7g** (EC_50_ = 184.07 ± 19.33 μM) > **F_2_-7g** (EC_50_ = 192.14 ± 4.57 μM), **7i** (EC_50_ = 15.02 ± 2.27 μM) > **F_2_-7i** (EC_50_ = 15.10 ± 0.68 μM), and **7j** (EC_50_ = 14.95 ± 4.83 μM) > **F_2_-7j** (EC_50_ = 16.55 ± 6.13 μM). When R_4_ was cyclopropyl (**7i**) and CF_3_ (**7j**), the compounds showed obvious selectivity for HIV-2.

Notably, the compounds with more fluorine atoms at the terminal of the two series had the most potent anti-HIV-1 activity, such as **F_2_-7f** and **F_2_-7j**. It is speculated that the F atom forms a hydrogen bond with the surrounding key amino acid residues. In addition, the anti-HIV activity of series I was generally better than that of series II, indicating that the indole derivatives for the NTD-CTD interface were more conducive to antiviral activity than the aniline derivatives. Preliminary SARs analysis of newly synthesized compounds revealed that the linker of **PF74** derivatives and the substituent components was particularly important for anti-HIV activity, which could serve as guidance for further research exploring the NTD-CTD interface.

Overall, the study of these compounds showed that the alteration of substituents had a significant impact on antiviral activity and selectivity, and **F_2_-7f** represented a promising lead compound for further optimization.

### 2.3. Surface Plasmon Resonance (SPR) Assay on CA Protein

Next, we selected the representative compound **F_2_-7f** for direct binding evaluation with monomeric and hexameric HIV-1 CA protein using SPR. We utilized the previously reported SPR method to test the binding affinity and off-rates of **F_2_-7f** to CA monomers and CA hexamers, using the lead compound **PF74** as an internal control [30].

**Figure 2:**
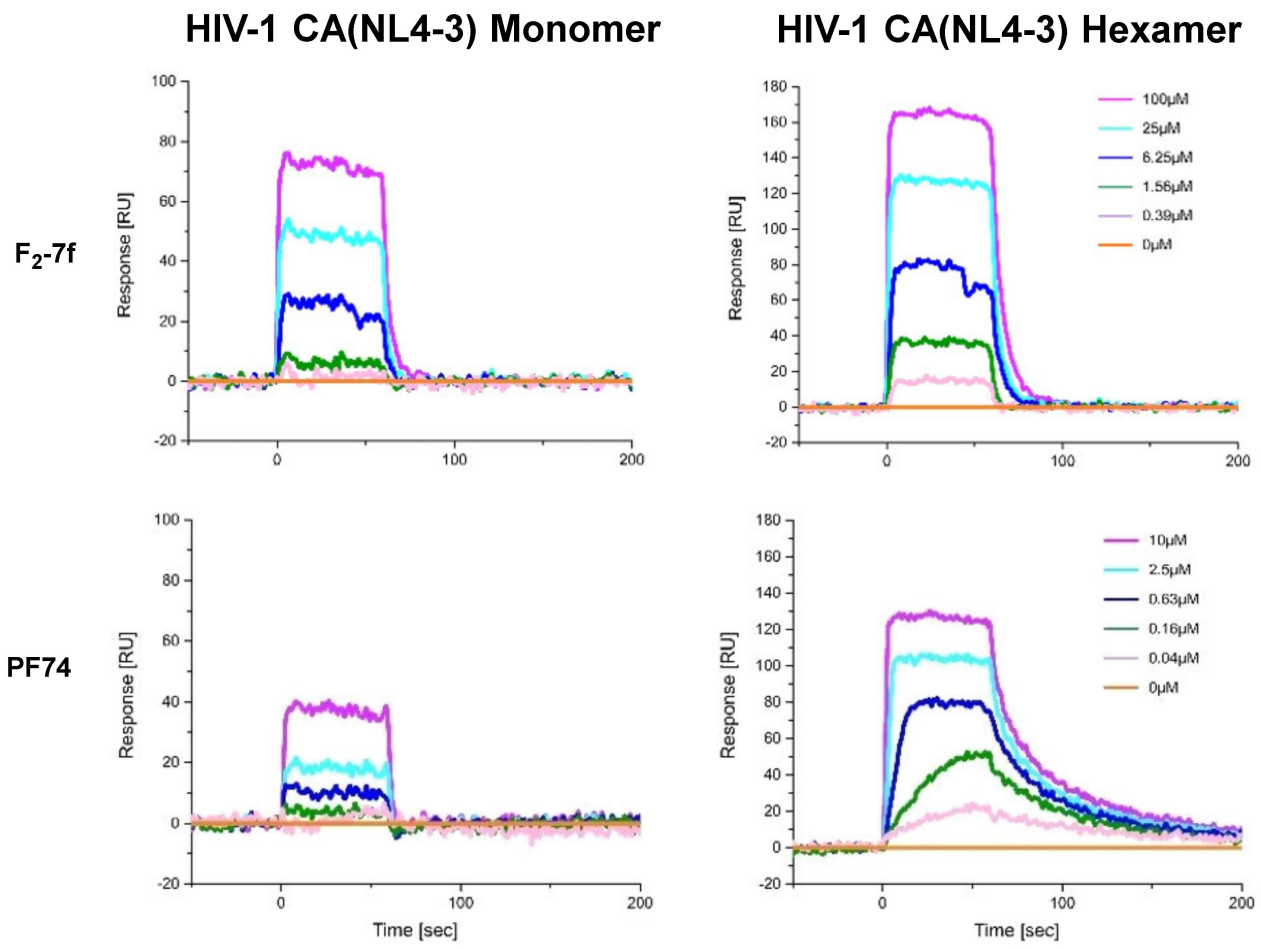
SPR sensorgram depicting the interaction of **F_2_-7f** and **PF74** with two HIV-1 CA variants (monomer and disulfide-stabilized hexamer).

**Figure 3:**
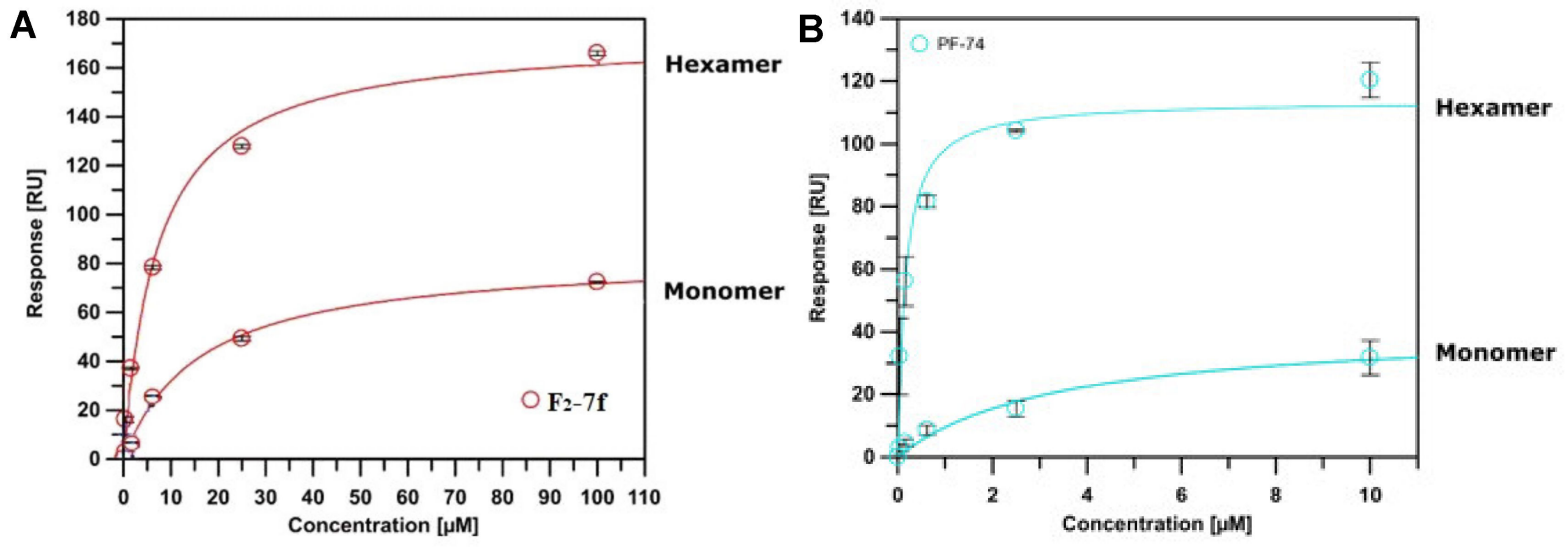
SPR isotherms of **F_2_-7f** (**A**) binding to two variants (monomer and disulfide-stabilized hexamer) of the CA protein using **PF74** (**B**) as the reference. Isotherms are an average of 3 replicates with error bars indicating the standard deviation (SD).

The SPR results are shown in Figure 2 and Figure 3 and Table 3, providing evidence for direct binding with both monomeric and hexameric CA proteins. Based on the equilibrium dissociation constant (K_D_), these compounds preferred to bind to the CA hexamer rather than to the CA monomer. The affinity of **PF74** (hexamer: K_D_ = 0.159 ± 0.041 μM; monomer: K_D_ = 3.410 ± 1.310 μM) to CA was greater than that of **F_2_-7f** (hexamer: K_D_ = 7.203 ± 1.101 μM; monomer: K_D_ = 16.063 ± 1.316 μM), which was consistent with their antiviral activity (**PF74**, EC_50_ = 0.75 ± 0.33 μM > **F_2_-7f**, EC_50_ = 5.89 ± 2.03 μM) in vitro. Simultaneously, for CA hexamers, the k_off_ values of **F_2_-7f** were approximately 8.5 times higher than that of **PF74**, indicating faster dissociation. As found in a previous study, the increase in k_off_ value was positively correlated with the decrease in antiviral activity [31], which suggested that the lower affinity and faster off-rate of **F_2_-7f** for HIV-1 CA might be the reason for its reduced anti-HIV-1 activity. Therefore, SPR experiments proved that these newly synthesized compounds could be defined as HIV-1 CA modulators.

### 2.4. Molecular Dynamics (MD) Simulation with **F_2_-7f** Bound HIV-1 CA Hexamer

**Figure 4:**
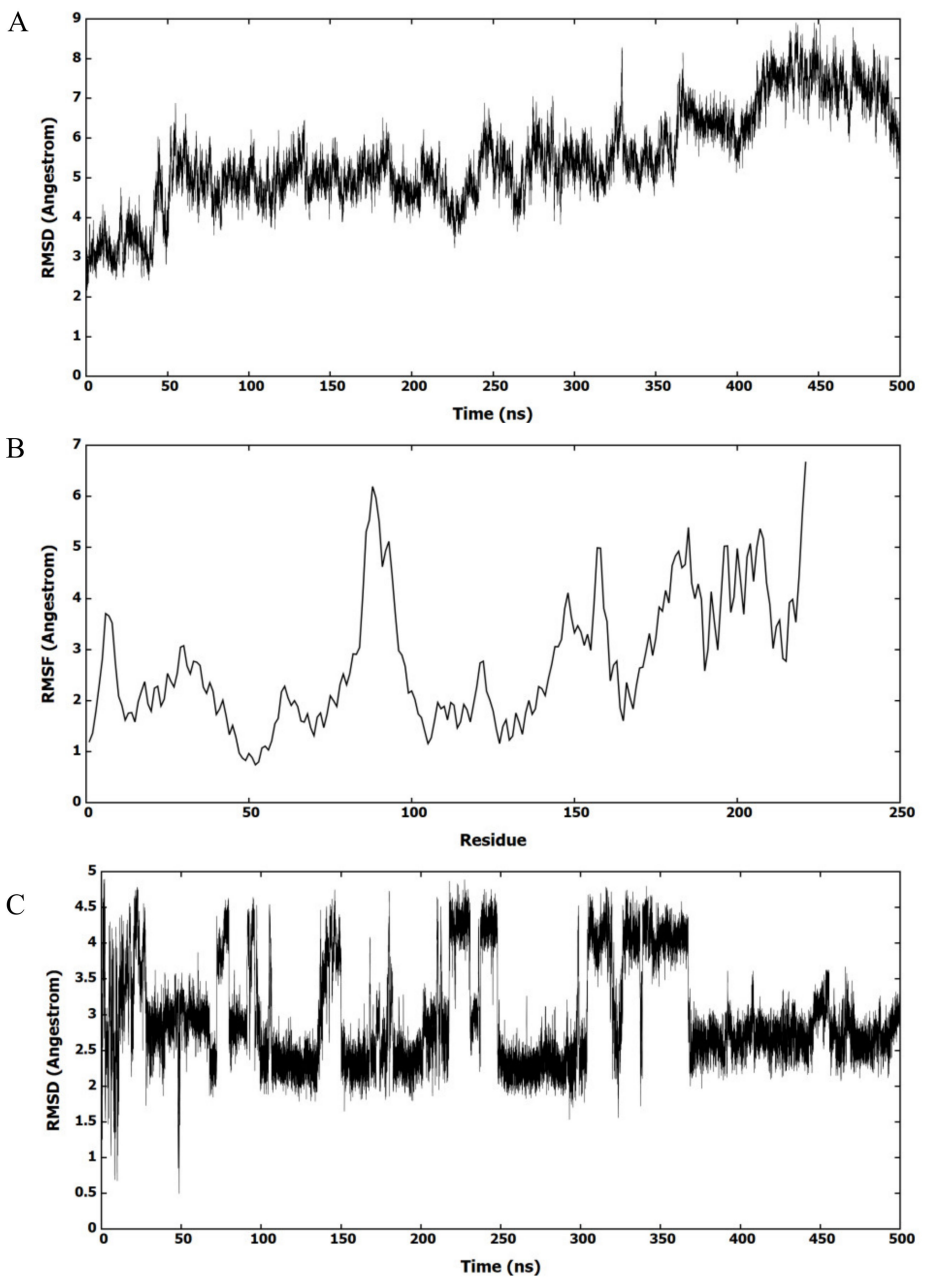
MD simulation of **F_2_-7f** bound HIV-1 CA. (**A**) RMSD of HIV-1 CA monomer amino acids refers to the first framework of the MD simulation. (**B**) RMSF of the backbone Cα atoms for amino acids of HIV-1 CA monomer. (**C**) RMSD (heavy atoms) of the bound **F_2_-7f**.

The most active molecule, **F_2_-7f**, was selected to investigate its binding to the ligand site of HIV-1 CA. Figure 4A shows the root mean square deviation (RMSD) values of HIV-1 CA residues from the first frame of the MD simulation. The figure highlights that the protein had largely deviated from the X-ray structure and formed diverse protein conformations. The root mean square fluctuation (RMSF) of amino acids was investigated to identify the deviated amino acids (Figure 4B). Most amino acids had largely deviated from the X-ray structure, indicating that HIV-1 CA had multiple conformations during MD simulation. This deviation of amino acids indicated that HIV-1 CA had different binding modes with **F_2_-7f**. To investigate the potential binding modes of **F_2_-7f** to the binding site, the RMSD values of **F_2_-7f** were calculated, as shown in Figure 4C. The figure showed that **F_2_-7f** had deviated from the docked conformation and clustered in different conformations.

**Figure 5:**
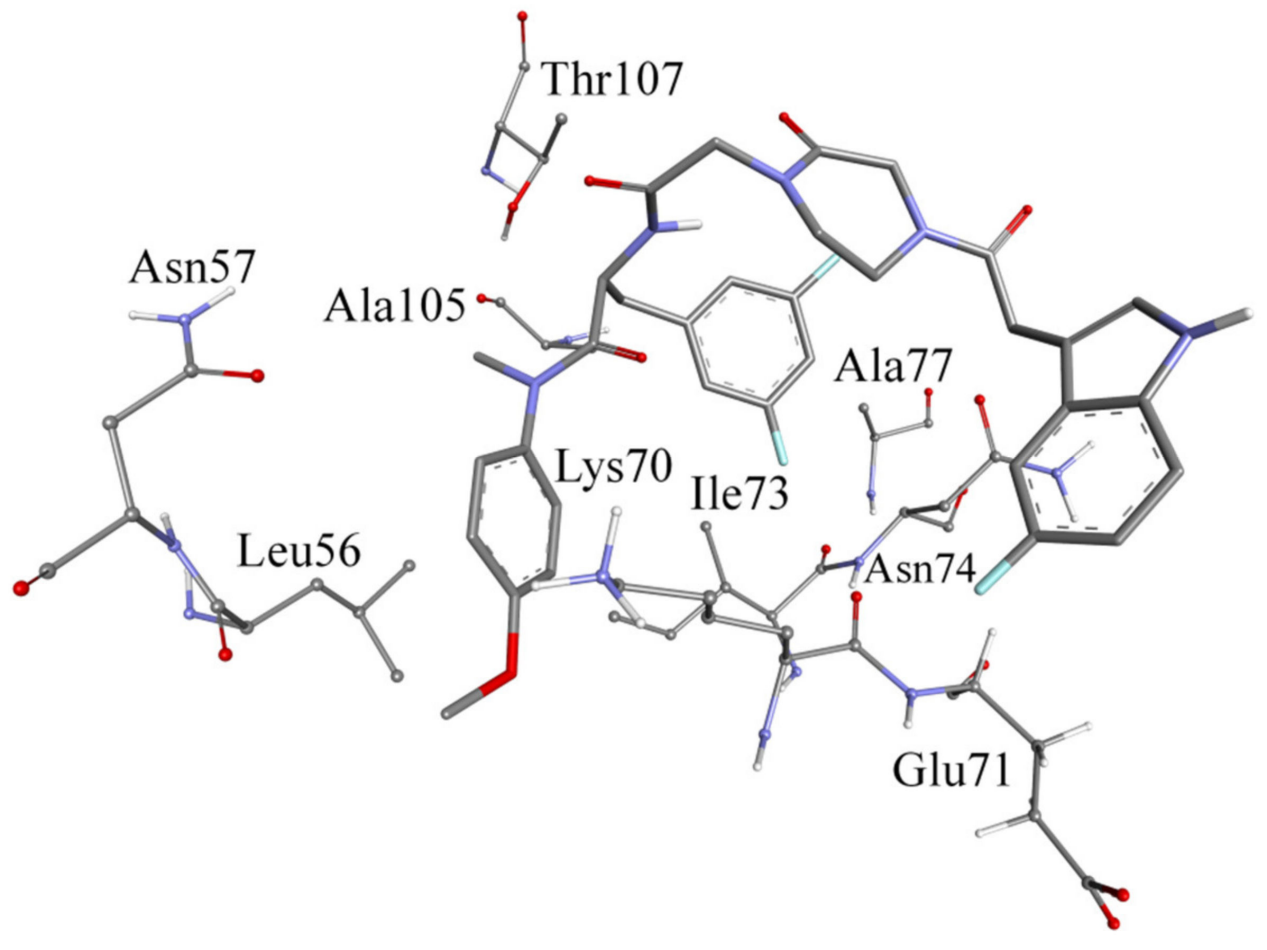
Binding of **F_2_-7f** to HIV-1 CA in the most populated cluster.

The MD trajectory was clustered based on **F_2_-7f** to explore its interactions with the binding site. The clustering procedure returned 10 clusters with one dominant. Figure 5 shows the representative structure of the most populated cluster. The conformation of the HIV-1 CA representative structure had a folded structure, which was different from the X-ray structure. The binding site in the representative structure was at the same location as the X-ray structure.

The representative structure of the dominant cluster was investigated to determine the bonding forces between HIV-1 CA and **F_2_-7f**. Methoxybenzene was embedded between Leu56 and Lys70, where it could be involved in aromatic-aliphatic hydrophobic interactions. Also, the benzene ring of methoxybenzene could be involved in ion-induced dipole with the charged nitrogen of Lys70. 3,5-difluorobenzene was involved in hydrophobic interactions with Ala105. Thr107 formed a hydrogen bond with the oxygen atom of the amide (24.5%), as shown in Figure 5. Glu71 formed a hydrogen bond with the indole ring of **F_2_-7f** in 20% of the MD simulations, which might explain why **F_2_-7f** was the most potent compound in this study. It could be seen that **F_2_-7f** and **PF74** bound in the same site, but the conformation of **F_2_-7f** was partially changed relative to that of **PF74**, resulting in the absence of some key interactions, which might explain its reduced antiviral activity.

### 2.5. In Silico Predicted ADMET Properties of Representative Compounds

#### 2.5.1. Drug-Like Properties and Metabolic Stability

**Figure 6:**
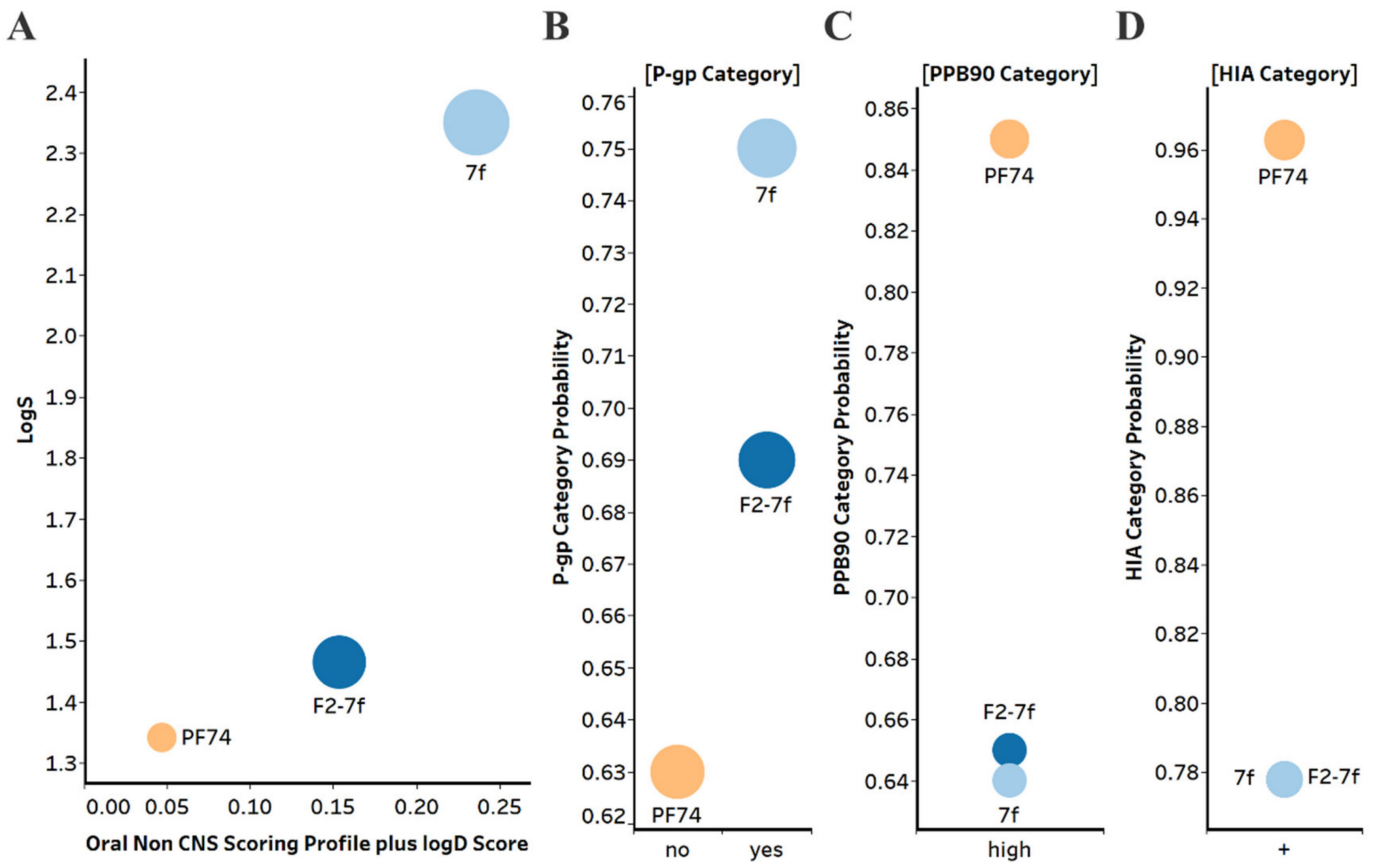
(**A**) Plot showing the StarDrop V7 (Optibrium, Ltd., Cambridge, UK)–derived logS versus a multimetric oral non-CNS profile score. Importance for the score: logS = 0.9, HIA = 0.85, logP = 0.6, logD = 0.6, hERG pIC50 = 0.4, 2D6 affinity category = 0.3, 2C9 pKi = 0.3, P-gp category = 0.3, PPB90 category = 0.2, BBB category = 0.11, BBB log(brain:blood) = 0.11 (**B**) P-gp category, (**C**) PPB90 = Plasma protein binding > 90%, (**D**) HIA = human intestinal absorption category, and probability of prediction of **7f**, **F_2_-7f** and **PF74**. The size of the circle correlates with the corresponding score or probability.

One of the main drawbacks of **PF74** is its low metabolic stability and poor drug-like property profile, which limits its clinical use. Therefore, we evaluated selected compounds from this study for their drug-like properties and metabolic stability and compared them with **PF74** (Figure 6). We utilized in silico prediction of drug-like metrics as implemented in the oral non–central nervous system (CNS) drug profile in StarDrop 7 software (Optibrium, Ltd., Cambridge, UK) [32]. This profile consists of several models, and a probabilistic scoring algorithm combines the model predictions in the oral non-CNS drug profile into an overall score. Scores range from 0 to 1, with 0 suggesting extremely non–drug-like and 1 suggesting the perfect drug.

The oral non-CNS drug profile in Optibrium’s StarDrop software combines logS (intrinsic aqueous solubility); classification for human intestinal absorption; logP (octanol/water); hERG (human ether-a-go-go-related gene) pIC_50_ (mammalian cells); cytochrome P450 CYP2D6 classification; cytochrome P450 CYP2C9 pKi values; classification of P-glycoprotein transport; classification of blood-brain barrier (BBB) penetration; and predicted BBB penetration value (Figure 6).

Based on this analysis, **F_2_-7f** and especially **7f** displayed improved aqueous solubility compared to **PF74,** as judged by the logS values. This can contribute to improved overall bioavailability. **F_2_-7f** and **7f** showed improved oral non-CNS drug profile scores, primarily due to improved solubility and low plasma protein binding compared to **PF74**.

**Figure 7:**
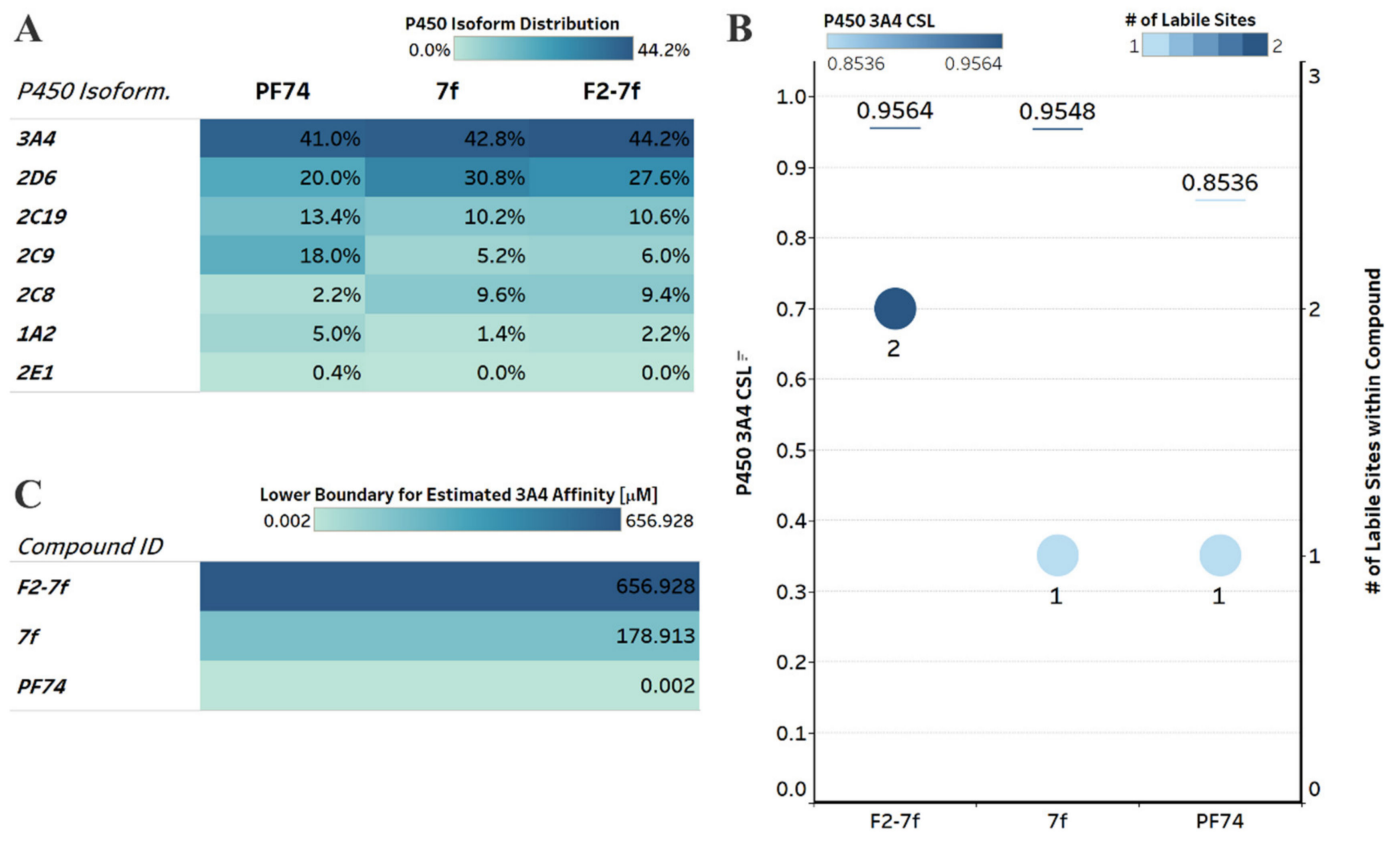
Computational prediction of metabolic stability. (**A**) Prediction of the major metabolizing CYP isoforms. All compounds are predicted to be metabolized primarily by the 3A4 isoform, including **PF74**. (**B**) Overall composite site lability (CSL) score and number of labile sites within the compound (for metabolism). A lower CSL score indicates a more stable molecule. The prediction was achieved using the StarDrop (version 7) P450 module. (**C**) Predicted affinity of docked **7f, F_2_-7f**, and **PF74** to Cytochrome P450 3A4 (PDB ID: 4D78). The lower boundary for predicted 3A4 affinity utilized the hydrogen bond and dehydration scoring function (HYDE) implemented in SeeSAR12.1.

The poor metabolic stability of **PF74** limits its use in clinical applications [31]. For orally administered drugs, the intestinal wall and portal circulation to the liver represents first-pass metabolism and can limit compound concentrations in the bloodstream. Therefore, we next sought to investigate in silico whether or not our compounds had improved predicted metabolic stability over **PF74**. We employed a computational analysis first demonstrated to be an accurate indicator of metabolic stability by the Cocklin group [33,34,35]. We utilized the P450 module in StarDrop 7 software (Optibrium, Ltd., Cambridge, UK) to predict each compound’s major metabolizing Cytochrome P450 isoforms using the WhichP450™ model. Subsequently, we predicted the affinity to that isoform using the HYDE function in See SAR (BioSolveIT Gmbh, Sankt Augustin, Germany) [34,36,37]. The results of this analysis are shown in Figure 7.

The main metabolizing isoform for all compounds, including **PF74**, is the CYP3A4 isoform [12] (Figure 7A). In addition, **F_2_-7f** and **7f** were also predicted to be metabolized to a greater extent than **PF74** by the 2D6 isoform and were within the high-affinity category for 2D6, according to the analysis in StarDrop.

We next investigated the predicted metabolic lability of our compounds and **PF74** with the CY3A4 isoform by comparing the overall composite site lability (CSL) score and number of labile sites. The CSL score can reflect the overall efficiency of metabolism of the molecule by combining the labilities of individual sites within the compound. The number of labile sites between our compounds and **PF74** was not significantly different; however, the CSL score indicated increased metabolic stability for **PF74** (Figure 7B).

In addition to the CSL score and number of labile sites, which assumed that all compounds bind with similar affinity to the CYP3A4 isoform, other factors such as compound reduction rate and actual binding affinity to the CYP3A4 isoform can influence metabolic stability. In addition, intrinsic compound properties, such as size and lipophilicity, can also infer affinity. Therefore, we performed predictive binding affinity calculations using the hydrogen bond and dehydration (HYDE) energy scoring function in SeeSAR 12.1 (BioSolveIT Gmbh, Sankt Augustin, Germany) [38] using the structure of the human CYPA4 bound to an inhibitor (PDB ID 4D78) [39]. The HYDE scoring function in SeeSAR provides a range of affinities, including an upper and lower limit. We used the lower limit as the affinity predictor to compare **F_2_-7f**, **7f**, and **PF74** (Figure 7C), which resulted in an affinity of 657 μM for **F_2_-7f**, 179 μM for **7f**, and 2 nM for **PF74**. Although **F_2_-7f** and **7f** had less favorable CSL scores and **F_2_-7f** had two labile sites (terminal methoxy and a carbon between the two F atoms within the difluorobenzene), the much lower predicted CYP3A4 affinities for **F_2_-7f** and **7f** might have compensated for the higher CSL and number of labile sites.

Combining the results from these predictions (CSL scores, labile sites, and predicted CYP3A4 affinity), this analysis indicated that compounds **F_2_-7f** and **7f** have similar metabolic stability as **PF74**.

#### 2.5.2. Genotoxicity and Hepatotoxicity

**Figure 8:**
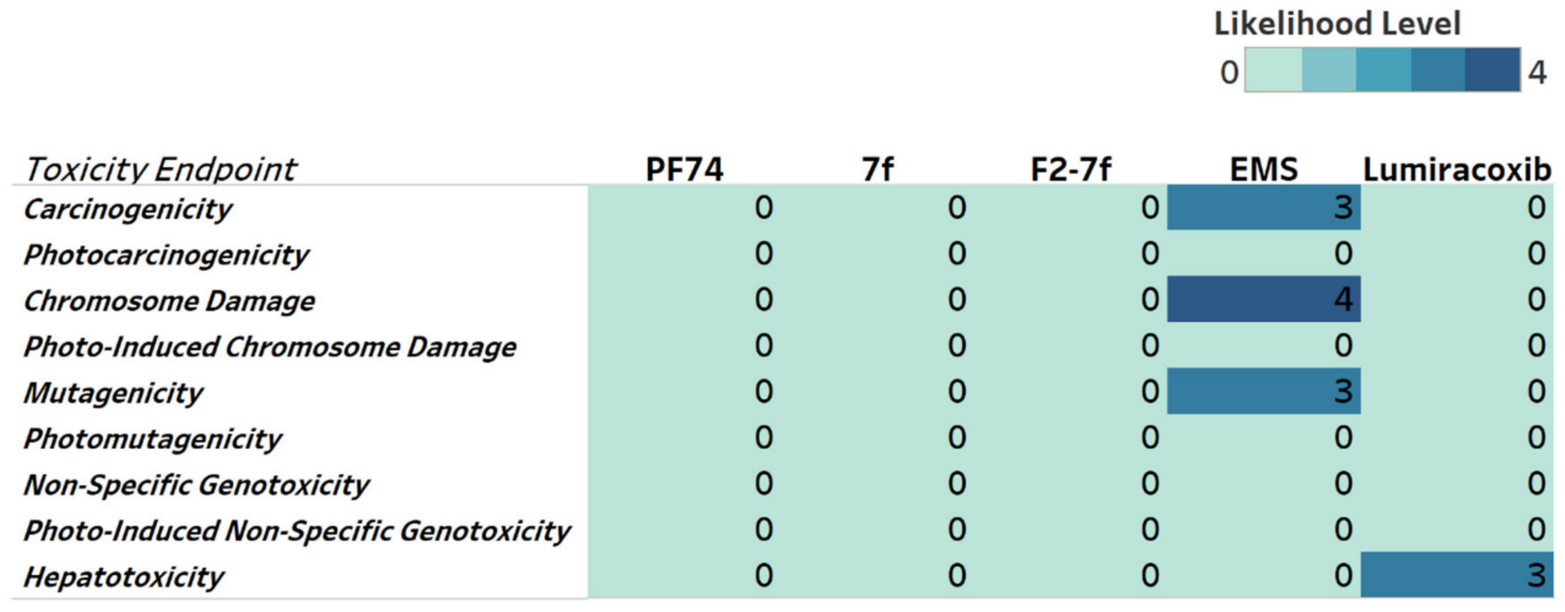
Genotoxicity and hepatotoxicity endpoints for selected compounds. The highlighted table shows the StarDrop V7 (Optibrium, Ltd., Cambridge, UK)–derived toxicity endpoints using the Derek Nexus module with knowledge-based prediction. Structural alerts within the molecule with a level of likelihood for concern are based on precedence from experimental data. Likelihood level 0 = No report; 1 = Inactive; 2 = Equivocal; 3 = Plausible; 4 = Probable. Ethyl methanesulfonate (EMS) was used as a positive control for genotoxicity, and lumiracoxib was a positive control for hepatotoxicity endpoints.

To obtain a toxicity profile for the lead compounds, we included genotoxicity and hepatotoxicity endpoints in our multiparameter optimization for **F_2_-7f**, **7f**, and **PF74** using the Derek Nexus module within StarDrop V7 software. Derek Nexus utilizes a knowledge- and rule-based expert system for semi-quantitative estimations of DNA-reactive moieties within molecules. None of the lead compounds nor **PF74** showed any concerning likelihood of genotoxicity or hepatotoxicity (Figure 8). To evaluate in silico the accuracy of the prediction, we used positive controls in our prediction. Ethyl methanesulfonate (EMS) [40] and lumiracoxib [41] are known to have in vivo genotoxic and hepatotoxic effects.

### 2.6. Metabolic Stability in the Presence of Human Liver Microsomes and Human Plasma

Equipped with the computational predictions, we next performed metabolic stability assays in human liver microsomes (HLMs) and human plasma. Firstly, testosterone, diclofenac, and propafenone with moderate metabolic stability were selected as control drugs, and we tested the metabolic stability of **F_2_-7f** and **PF74** in HLMs. As shown in Table 4, **F_2_-7f** and **PF74** could be rapidly metabolized in HLMs with a half-life of 0.5 min. The intrinsic clearance (CL_int_) of **F_2_-7f** was slightly lower than that of **PF74** (2759.1 and 2862.5 μL/min/mg, respectively). The results of the human plasma stability assay for **F_2_-7f** and **PF74** are shown in Table 5. After 120 min of incubation, the residual amount of the original **F_2_-7f** decreased to 86.9%, and the residual amount of the original **PF74** decreased to 85.2%, indicating that the metabolic stability of **F_2_-7f** in human plasma was slightly improved compared to that of **PF74**. This concurred with the lower plasma protein binding prediction probability for **F_2_-7f** (PPB90, Figure 6C). Overall, the experimental data moderately correlated with the computational prediction, and improving the metabolic stability of **PF74**-like small molecules remains an urgent issue for future optimization efforts.

## 3. Experimental Section

### 3.1. Chemistry

All melting points (mp) of the new compounds were determined on a micro melting point apparatus. ^1^H NMR and ^13^C NMR spectra were obtained in DMSO-*d*_6_ on a Bruker AV-400 spectrometer or Bruker AV-600 spectrometer using tetramethylsilane (TMS) as the internal reference. Chemical shifts were reported in *δ* values (ppm) and *J* values were expressed in hertz (Hz). Thin layer chromatography (TLC) for monitoring reactions or purifying products was performed on silica gel GF254 or Huanghai HSGF254, 0.15–0.2 mm, respectively. Spots were visualized with iodine vapor or by irradiation with UV light (*λ* = 254 nm or λ = 365 nm). Mass spectrometry (MS) was carried out using a Standard G1313A LC autosampler instrument. Flash column chromatography was performed on a column packed with silica gel 60 (200–300 mesh). Solvents were of reagent grade and, if needed, were purified and dried using standard methods. Rotary evaporators were involved in concentrating the reaction solutions under reduced pressure. The solvents dichloromethane, TEA, methanol, etc. were obtained from Sinopharm Chemical Reagent Co., Ltd. (SCRC, Shanghai, China) and were of AR grade. The key reactants, including 4-methoxy-*N*-methylaniline (CAS: 5961-59-1), *N*-(*tert*-butoxycarbonyl)-*L*-phenylalanine (CAS: 13734-34-4), and *N*-(*tert*-butoxycarbonyl)-3,5-difluoro-*L*-phenylalanine (CAS: 205445-52-9), were purchased from Shanghai Haohong Scientific Co., Ltd. (Shanghai, China). The purity of all target compounds was analyzed by high-performance liquid chromatography (HPLC) and was >95%.

#### 3.1.1. Procedure for the Synthesis of Intermediates

The procedure for the synthesis of key intermediates is provided in the Supplementary Materials [29,30].

#### 3.1.2. General Procedure for the Synthesis of **7 (a-f)** and **F_2_-7 (a-f)**

Corresponding substituted indoleacetic acids (1.2 eq.) were first dissolved in 20 mL dichloromethane (DCM), and then HATU (1.5 eq.) was added. The reaction mixture was stirred at 0 °C for 0.5 h. Then, the key intermediates **6** and **F_2_-6** (1 eq.) were added dropwise into the solution, and DIEA (2 eq.) was added. The reaction mixture was restored at room temperature for 3 h. After monitoring the reaction by TLC, the excess solvent was evaporated under reduced pressure, and the residue was redissolved in water and extracted with DCM (3 × 20 mL). Subsequently, the organic layers were combined and washed with saturated sodium bicarbonate (3 × 20 mL), dried over anhydrous Na_2_SO_4_, filtered, and concentrated under reduced pressure to obtain the corresponding crude products. These products were purified by flash column chromatography to provide target compounds **7** (**a-f**) and **F_2_-7** (**a-f**).

##### (S)-N-(4-methoxyphenyl)-N-methyl-2-(2-(4-(2-(2-methyl-1H-indol-3-yl)acetyl)-2-oxopiperazin-1-yl)acetamido)-3-phenylpropanamide (**7a**)

White solid. Yield: 72%. Purity: 99%. Mp: 109–111 °C. ^1^H NMR (400 MHz, DMSO-d_6_) δ 10.78 (s, 1H, NH), 8.35 (d, J = 7.9 Hz, 1H, NH), 7.40 (d, J = 7.7 Hz, 1H, Ph-H), 7.23 (d, J = 7.9 Hz, 1H, Ph-H), 7.20–7.06 (m, 5H, Ph-H), 6.97 (d, J = 8.4 Hz, 3H, Ph-H), 6.91 (d, J = 7.0 Hz, 1H, Ph-H), 6.84 (d, J = 6.6 Hz, 2H, Ph-H), 4.47 (q, J = 8.5 Hz, 1H, CH), 4.14 (s, 1H, CH), 3.99 (d, J = 5.6 Hz, 1H, CH), 3.96–3.83 (m, 2H, CH_2_), 3.79 (s, 3H, OCH_3_), 3.72 (d, J = 7.9 Hz, 2H, CH_2_), 3.68 (s, 1H, CH), 3.60 (s, 1H, CH), 3.10 (s, 3H, NCH_3_), 3.08 (s, 2H, CH_2_), 2.85 (dd, J = 13.5, 4.7 Hz, 1H, PhCH), 2.64 (dd, J = 13.1, 9.7 Hz, 1H, PhCH), 2.31 (s, 3H, CH_3_). ^13^C NMR (150 MHz, DMSO-d_6_) δ 171.40 (C=O), 169.91 (C=O), 167.58 (C=O), 159.04 (C=O), 137.93, 135.98, 135.60, 129.32, 129.12, 128.82, 128.57, 126.85, 120.47, 118.68, 118.15, 115.18, 110.78, 55.91, 51.81, 47.21, 37.79, 11.92. HRMS (ESI) calculated for C_34_H_37_N_5_O_5_ [M+H]^+^ 596.2867, found 596.2872.

##### (S)-2-(2-(4-(2-(5-methoxy-1H-indol-3-yl)acetyl)-2-oxopiperazin-1-yl)acetamido)-N-(4-methoxyphenyl)-N-methyl-3-phenylpropanamide (**7b**)

White solid. Yield: 69%. Purity: 99%. Mp: 142–144 °C. ^1^H NMR (400 MHz, DMSO-d_6_) δ 10.77 (d, J = 8.9 Hz, 1H, NH), 8.40 (d, J = 7.9 Hz, 1H, NH), 7.24 (d, J = 8.7 Hz, 1H, Ph-H), 7.19 (s, 1H, Ph-H), 7.18–7.09 (m, 5H, Ph-H), 7.06 (s, 1H, Ph-H), 6.98 (d, J = 8.3 Hz, 2H, Ph-H), 6.85 (d, J = 6.6 Hz, 2H, Ph-H), 6.73 (d, J = 8.7 Hz, 1H, Ph-H), 4.54–4.41 (m, 1H, CH), 4.18 (s, 1H, CH), 4.03 (t, J = 6.7 Hz, 1H, CH), 3.99–3.84 (m, 2H, CH_2_), 3.79 (s, 3H, OCH_3_), 3.77 (s, 2H, CH_2_), 3.74 (s, 3H, OCH_3_), 3.71(m, 1H, CH), 3.63 (s, 1H, CH), 3.11 (s, 5H, NCH_3_, CH_2_), 2.86 (dd, J = 13.4, 4.3 Hz, 1H, PhCH), 2.71–2.59 (m, 1H, PhCH). ^13^C NMR (100 MHz, DMSO-d_6_) δ 171.42 (C=O), 169.78 (C=O), 167.64 (C=O), 159.02 (C=O), 137.94, 135.94, 131.73, 129.32, 129.14, 128.60, 126.88, 124.69, 115.16, 112.46, 111.66, 101.08, 55.90, 55.83, 51.88, 51.84, 37.78, 37.73, 30.81. HRMS (ESI) calculated for C_34_H_37_N_5_O_6_ [M+H]^+^ 612.2817, found 612.2819.

##### (S)-2-(2-(4-(2-(5-bromo-1H-indol-3-yl)acetyl)-2-oxopiperazin-1-yl)acetamido)-N-(4-methoxyphenyl)-N-methyl-3-phenylpropanamide (**7c**)

White solid. Yield: 70%. Purity: 99%. Mp: 115–116 °C. ^1^H NMR (400 MHz, DMSO-d_6_) δ 11.11 (d, J = 8.8 Hz, 1H, NH), 8.37 (d, J = 7.8 Hz, 1H, NH), 7.74 (s, 1H, Ph-H), 7.30 (t, J = 10.3 Hz, 2H, Ph-H), 7.14 (d, J = 13.2 Hz, 6H, Ph-H), 6.97 (d, J = 8.3 Hz, 2H, Ph-H), 6.84 (d, J = 6.6 Hz, 2H, Ph-H), 4.54–4.42 (m, 1H, CH), 4.20 (s, 1H, CH), 4.01 (q, J = 9.9 Hz, 1H, CH), 3.97–3.88 (m, 2H, CH_2_), 3.86 (s, 2H, CH_2_), 3.79 (s, 3H, OCH_3_), 3.73 (s, 1H, CH), 3.62 (s, 1H, CH), 3.15 (s, 2H, CH_2_), 3.11 (s, 3H, NCH_3_), 2.85 (dd, J = 13.3, 4.1 Hz, 1H, PhCH), 2.71–2.59 (m, 1H, PhCH). ^13^C NMR (150 MHz, DMSO-d_6_) δ 171.40 (C=O), 169.51 (C=O), 167.61 (C=O), 159.04 (C=O), 137.93, 135.98, 135.33, 129.68, 129.33, 129.11, 128.57, 126.86, 125.85, 123.96, 121.68, 115.18, 113.81, 111.59, 108.20, 55.91, 51.80, 49.23, 46.23, 42.91, 37.79, 30.38. HRMS (ESI) calculated for C_33_H_34_BrN_5_O_5_ [M+H]^+^ 660.1816, found 660.1819.

##### (S)-2-(2-(4-(2-(1H-indol-3-yl)acetyl)-2-oxopiperazin-1-yl)acetamido)-N-(4-methoxyphenyl)-N-methyl-3-phenylpropanamide (**7d**)

White solid. Yield: 75%. Purity: 99%. Mp: 170–171 °C. ^1^H NMR (400 MHz, DMSO-d_6_) δ 10.90 (d, J = 7.6 Hz, 1H, NH), 8.38 (d, J = 7.8 Hz, 1H, NH), 7.55 (d, J = 7.4 Hz, 1H, Ph-H), 7.34 (d, J = 8.0 Hz, 1H, Ph-H), 7.21 (d, J = 11.6 Hz, 1H, Ph-H), 7.13 (dt, J = 15.4, 6.2 Hz, 5H, Ph-H), 7.06 (d, J = 7.5 Hz, 1H, Ph-H), 6.97 (d, J = 8.0 Hz, 3H, Ph-H), 6.84 (d, J = 6.7 Hz, 2H, Ph-H), 4.46 (q, J = 7.5 Hz, 1H, CH), 4.17 (s, 1H, CH), 4.00 (q, J = 13.0, 10.9 Hz, 1H, CH), 3.96–3.83 (m, 2H, CH_2_), 3.80 (s, 2H, CH_2_), 3.78 (s, 3H, OCH_3_), 3.72 (s, 1H, CH), 3.61 (s, 1H, CH), 3.10 (s, 5H, NCH_3_, CH_2_), 2.85 (dd, J = 13.3, 4.3 Hz, 1H, PhCH), 2.71–2.59 (m, 1H, PhCH). ^13^C NMR (100 MHz, DMSO-d_6_) δ 171.42 (C=O), 169.73 (C=O), 167.63 (C=O), 159.02 (C=O), 137.94, 136.60, 135.95, 129.32, 129.14, 128.60, 127.67, 126.88, 124.07, 121.53, 119.22, 118.86, 115.16, 111.81, 55.90, 51.83, 48.52, 47.36, 46.16, 42.93, 30.79. HRMS (ESI) calculated for C_33_H_35_N_5_O_5_ [M+H]^+^ 582.2711, found 582.2716.

##### (S)-2-(2-(4-(2-(5-hydroxy-1H-indol-3-yl)acetyl)-2-oxopiperazin-1-yl)acetamido)-N-(4-methoxyphenyl)-N-methyl-3-phenylpropanamide (**7e**)

White solid. Yield: 78%. Purity: 99%. Mp: 131–132 °C. ^1^H NMR (400 MHz, DMSO-d_6_) δ 10.60 (d, J = 5.8 Hz, 1H, NH), 8.60 (s, 1H, OH), 8.39 (d, J = 7.8 Hz, 1H, NH), 7.24–7.06 (m, 7H, Ph-H), 6.98 (d, J = 8.2 Hz, 2H, Ph-H), 6.92–6.81 (m, 3H, Ph-H), 6.60 (d, J = 8.6 Hz, 1H, Ph-H), 4.54–4.42 (m, 1H, CH), 4.16 (s, 1H, CH), 4.02 (p, J = 11.4, 9.9 Hz, 1H, CH), 3.96–3.84 (m, 2H, CH_2_), 3.80 (s, 3H, OCH_3_), 3.73 (d, J = 15.2 Hz, 2H, CH_2_), 3.69 (s, 1H, CH), 3.62 (s, 1H, CH), 3.11 (s, 5H, NCH_3_, CH_2_), 2.86 (dd, J = 13.0, 3.9 Hz, 1H, PhCH), 2.71–2.60 (m, 1H, PhCH). ^13^C NMR (100 MHz, DMSO-d_6_) δ 171.42 (C=O), 169.76 (C=O), 167.64 (C=O), 159.02 (C=O), 150.78, 137.94, 135.94, 131.18, 129.32, 129.15, 128.60, 128.34, 126.89, 124.45, 115.17, 112.09, 111.95, 107.19, 103.24, 55.90, 51.82, 48.52, 47.36, 46.14, 37.79. HRMS (ESI) calculated for C_33_H_35_N_5_O_6_ [M+H]^+^ 598.2660, found 598.2658.

##### (S)-2-(2-(4-(2-(5-fluoro-1H-indol-3-yl)acetyl)-2-oxopiperazin-1-yl)acetamido)-N-(4-methoxyphenyl)-N-methyl-3-phenylpropanamide (**7f**)

White solid. Yield: 72%. Purity: 97%. Mp: 95–96 °C. ^1^H NMR (400 MHz, DMSO-d_6_) δ 11.00 (d, J = 8.4 Hz, 1H, NH), 8.37 (d, J = 8.0 Hz, 1H, NH), 7.37–7.27 (m, 3H, Ph-H), 7.14 (dd, J = 19.0, 6.6 Hz, 5H, Ph-H), 6.97 (d, J = 8.5 Hz, 2H, Ph-H), 6.91 (t, J = 9.1 Hz, 1H, Ph-H), 6.84 (d, J = 6.9 Hz, 2H, Ph-H), 4.47 (q, J = 8.2 Hz, 1H, CH), 4.19 (s, 1H, CH), 4.07–3.97 (m, 1H, CH), 3.97–3.84 (m, 2H, CH_2_), 3.79 (s, 3H, OCH_3_), 3.76 (s, 2H, CH_2_), 3.75–3.69 (m, 1H, CH), 3.65–3.58 (m, 1H, CH), 3.13 (s, 2H, CH_2_), 3.11 (s, 3H, NCH_3_), 2.85 (dd, J = 13.5, 4.8 Hz, 1H, PhCH), 2.65 (dd, J = 13.1, 9.6 Hz, 1H, PhCH). ^13^C NMR (150 MHz, DMSO-d_6_) δ 171.40 (C=O), 169.59 (C=O), 167.60 (C=O), 159.04 (C=O), 157.19 (d, ^1^J_C-F_ = 231.0 Hz), 137.93, 135.98, 133.29, 129.32, 129.11, 128.57, 126.85, 126.23, 115.18, 112.73 (d, ^3^J_C-F_ = 10.0 Hz), 109.61 (d, ^2^J_C-F_ = 26.2 Hz), 108.53, 103.95 (d, ^2^J_C-F_ = 23.4 Hz), 55.91, 51.80, 48.59, 46.21, 42.93, 37.79. HRMS (ESI) calculated for C_33_H_34_FN_5_O_5_ [M+H]^+^ 600.2617, found 600.2619.

##### (S)-3-(3,5-difluorophenyl)-N-(4-methoxyphenyl)-N-methyl-2-(2-(4-(2-(2-methyl-1H-indol-3-yl)acetyl)-2-oxopiperazin-1-yl)acetamido)propenamide (**F_2_-7a**)

White solid. Yield: 68%. Purity: 99%. Mp: 106–108 °C. ^1^H NMR (400 MHz, DMSO-d_6_) δ 10.81 (s, 1H, NH), 8.43 (d, J = 7.9 Hz, 1H, NH), 7.41 (d, J = 7.7 Hz, 1H, Ph-H), 7.24 (t, J = 6.3 Hz, 3H, Ph-H), 7.03 (d, J = 8.0 Hz, 3H, Ph-H), 7.01–6.95 (m, 1H, Ph-H), 6.91 (t, J = 7.3 Hz, 1H, Ph-H), 6.50 (d, J = 7.1 Hz, 2H, Ph-H), 4.46 (s, 1H, CH), 4.20–4.11 (m, 1H, CH), 4.06–3.99 (m, 1H, CH), 3.94 (d, J = 20.4 Hz, 2H, CH_2_), 3.80 (s, 3H, OCH_3_), 3.75 (s, 1H, CH), 3.72 (s, 2H, CH_2_), 3.68–3.56 (m, 1H, CH), 3.17 (s, 2H, CH_2_), 3.14 (s, 3H, NCH_3_), 2.92–2.83 (m, 1H, PhCH), 2.75–2.64 (m, 1H, PhCH), 2.32 (s, 3H, CH_3_). ^13^C NMR (100 MHz, DMSO-d_6_) δ 170.88 (C=O), 169.88 (C=O), 167.76 (C=O), 165.80 (C=O), 162.50 (dd, ^1^J_C-F_ = 245.6 Hz, ^3^J_C-F_ = 13.4 Hz), 159.16, 142.54 (t, ^3^J_C-F_ = 9.5 Hz), 135.82, 135.56, 133.31, 128.80, 120.47, 118.67, 118.18, 115.27, 112.35 (d, ^2^J_C-F_ = 24.6 Hz), 110.78, 104.08, 102.50 (t, ^2^J_C-F_ = 25.7 Hz), 55.92, 51.49, 48.57, 47.39, 46.23, 42.74, 37.75, 29.64, 11.92. HRMS (ESI) calculated for C_34_H_35_F_2_N_5_O_5_ [M+H]^+^ 632.2679, found 632.2679.

##### (S)-3-(3,5-difluorophenyl)-2-(2-(4-(2-(5-methoxy-1H-indol-3-yl)acetyl)-2-oxopiperazin-1-yl)acetamido)-N-(4-methoxyphenyl)-N-methylpropanamide (**F_2_-7b**)

White solid. Yield: 73%. Purity: 99%. Mp: 108–109 °C. ^1^H NMR (400 MHz, DMSO-d_6_) δ 10.81 (d, J = 7.0 Hz, 1H, NH), 8.48 (d, J = 7.9 Hz, 1H, NH), 7.28 (d, J = 8.1 Hz, 3H, Ph-H), 7.22 (d, J = 9.9 Hz, 1H, Ph-H), 7.16–7.03 (m, 4H, Ph-H), 6.77 (d, J = 8.6 Hz, 1H, Ph-H), 6.54 (d, J = 7.1 Hz, 2H, Ph-H), 4.50 (s, 1H, CH), 4.30–4.17 (m, 1H, CH), 4.08 (t, J = 5.2 Hz, 1H, CH), 4.03–3.90 (m, 2H, CH_2_), 3.84 (s, 3H, OCH_3_), 3.82 (s, 1H, CH), 3.79 (s, 2H, CH_2_), 3.79 (s, 3H, OCH_3_), 3.67 (ddt, J = 26.0, 13.8, 6.8 Hz, 1H, CH), 3.27–3.19 (m, 2H, CH_2_), 3.18 (s, 3H, NCH_3_), 2.97–2.87 (m, 1H, PhCH), 2.79–2.69 (m, 1H, PhCH). ^13^C NMR (100 MHz, DMSO-d_6_) δ 170.88 (C=O), 169.82 (C=O), 167.79 (C=O), 165.75 (C=O), 162.51 (dd, ^1^J_C-F_ = 245.4 Hz, ^3^J_C-F_ = 13.6 Hz), 159.16, 153.54, 142.53 (t, ^3^J_C-F_ = 9.4 Hz), 135.82, 131.73, 129.17, 128.01, 124.69, 115.27, 112.35 (d, ^2^J_C-F_ = 24.4 Hz), 111.67, 107.85, 102.49 (t, ^2^J_C-F_ = 25.8 Hz), 101.09, 55.92, 55.82, 51.49, 48.62, 47.54, 46.15, 42.96, 37.76, 37.14, 30.80. HRMS (ESI) calculated for C_34_H_35_F_2_N_5_O_6_ [M+H]^+^ 648.2628, found 648.2625.

##### (S)-2-(2-(4-(2-(5-bromo-1H-indol-3-yl)acetyl)-2-oxopiperazin-1-yl)acetamido)-3-(3,5-difluorophenyl)-N-(4-methoxyphenyl)-N-methylpropanamide (**F_2_-7c**)

White solid. Yield: 72%. Purity: 99%. Mp: 120–121 °C. ^1^H NMR (400 MHz, DMSO-d_6_) δ 11.13 (d, J = 7.1 Hz, 1H, NH), 8.43 (d, J = 7.9 Hz, 1H, NH), 7.74 (s, 1H, Ph-H), 7.30 (t, J = 9.8 Hz, 2H, Ph-H), 7.24 (d, J = 7.9 Hz, 2H, Ph-H), 7.18 (d, J = 8.6 Hz, 1H, Ph-H), 7.02 (d, J = 8.1 Hz, 3H, Ph-H), 6.49 (d, J = 7.1 Hz, 2H, Ph-H), 4.45 (s, 1H, CH), 4.26–4.14 (m, 1H, CH), 4.03 (d, J = 8.0 Hz, 1H, CH), 3.91 (t, J = 12.1 Hz, 2H, CH_2_), 3.82 (m, 1H, CH), 3.79 (s, 3H, OCH_3_), 3.76 (s, 2H, CH_2_), 3.71–3.55 (m, 1H, CH), 3.18 (d, J = 26.9 Hz, 2H, CH_2_), 3.13 (s, 3H, NCH_3_), 2.92–2.82 (m, 1H, PhCH), 2.69 (dd, J = 13.2, 9.8 Hz, 1H, PhCH). ^13^C NMR (100 MHz, DMSO-d_6_) δ 170.89 (C=O), 169.47 (C=O), 165.73 (C=O), 165.73 (C=O), 162.50 (dd, ^1^J_C-F_ = 245.8 Hz, ^3^J_C-F_ = 13.6 Hz), 159.16, 142.54 (t, ^3^J_C-F_ = 9.4 Hz), 135.82, 135.28, 129.67, 129.18, 125.87, 123.95, 121.71, 115.27, 113.82, 112.36 (dd, ^2^J_C-F_ = 19.7 Hz), 111.53, 108.10, 102.52 (t, ^2^J_C-F_ = 25.8 Hz), 55.92, 51.50, 47.51, 46.17, 42.85, 37.75, 37.12, 30.35. HRMS (ESI) calculated for C_33_H_32_BrF_2_N_5_O_5_ [M+H]^+^ 696.1628, found 696.1632.

##### (S)-2-(2-(4-(2-(1H-indol-3-yl)acetyl)-2-oxopiperazin-1-yl)acetamido)-3-(3,5-difluorophenyl)-N-(4-methoxyphenyl)-N-methylpropanamide (**F_2_-7d**)

White solid. Yield: 71%. Purity: 96%. Mp: 91–92 °C. ^1^H NMR (600 MHz, DMSO-d_6_) δ 10.88 (d, J = 8.7 Hz, 1H, NH), 8.38 (dd, J = 7.7, 3.0 Hz, 1H, NH), 7.59–7.52 (m, 1H, Ph-H), 7.38–7.31 (m, 1H, Ph-H), 7.22 (t, J = 10.8 Hz, 3H, Ph-H), 7.07 (t, J = 7.5 Hz, 1H, Ph-H), 7.01 (d, J = 8.3 Hz, 3H, Ph-H), 6.99–6.94 (m, 1H, Ph-H), 6.50 (d, J = 7.7 Hz, 2H, Ph-H), 4.51–4.42 (m, 1H, CH), 4.24–4.12 (m, 1H, CH), 4.06–3.97 (m, 1H, CH), 3.92 (q, J = 16.8 Hz, 2H, CH_2_), 3.80 (s, 1H, CH), 3.79 (s, 3H, OCH_3_), 3.75 (s, 2H, CH_2_), 3.71–3.57 (m, 1H, CH), 3.17 (d, J = 21.0 Hz, 2H, CH_2_), 3.13 (s, 3H, NCH_3_), 2.90–2.83 (m, 1H, PhCH), 2.74–2.65 (m, 1H, PhCH). ^13^C NMR (150 MHz, DMSO-d_6_) δ 170.86 (C=O), 169.78 (C=O), 169.75 (C=O), 165.78 (C=O), 162.53 (dd, ^1^J_C-F_ = 246.0 Hz, ^3^J_C-F_ = 13.1 Hz), 159.18, 142.53 (t, ^3^J_C-F_ = 9.4 Hz), 136.64, 135.86, 129.13, 127.70, 124.05, 121.51, 119.19, 118.86, 115.30, 112.36 (dd, ^2^J_C-F_ = 19.8 Hz), 111.80, 108.12, 102.45 (t, ^2^J_C-F_ = 25.9 Hz), 55.93, 51.44, 48.64, 47.50, 46.20, 42.99, 37.76, 37.26, 30.76. HRMS (ESI) calculated for C_33_H_33_F_2_N_5_O_5_ [M+H]^+^ 618.2523, found 618.2526.

##### (S)-3-(3,5-difluorophenyl)-2-(2-(4-(2-(5-hydroxy-1H-indol-3-yl)acetyl)-2-oxopiperazin-1-yl)acetamido)-N-(4-methoxyphenyl)-N-methylpropanamide (**F_2_-7e**)

White solid. Yield: 71%. Purity: 96%. Mp: 131–132 °C. ^1^H NMR (400 MHz, DMSO-d_6_) δ 10.59 (s, 1H, NH), 8.59 (s, 1H, OH), 8.41 (d, J = 7.8 Hz, 1H, NH), 7.23 (d, J = 7.6 Hz, 2H, Ph-H), 7.11 (t, J = 8.9 Hz, 2H, Ph-H), 7.02 (d, J = 7.8 Hz, 3H, Ph-H), 6.87 (s, 1H, Ph-H), 6.59 (d, J = 8.6 Hz, 1H, Ph-H), 6.49 (d, J = 7.3 Hz, 2H, Ph-H), 4.45 (s, 1H, CH), 4.23–4.09 (m, 1H, CH), 4.05–3.98 (m, 1H, CH), 3.98–3.85 (m, 2H, CH_2_), 3.79 (s, 3H, OCH_3_), 3.73 (s, 1H, CH), 3.69 (d, J = 10.0 Hz, 2H, CH_2_), 3.66–3.53 (m, 1H, CH), 3.22–3.14 (m, 2H, CH_2_), 3.13 (s, 3H, NCH_3_), 2.91–2.82 (m, 1H, PhCH), 2.74–2.64 (m, 1H, PhCH). ^13^C NMR (100 MHz, DMSO-d_6_) δ 170.87 (C=O), 169.75 (C=O), 167.73 (C=O), 165.75 (C=O), 162.51 (dd, ^1^J_C-F_ = 246.0 Hz, ^3^J_C-F_ = 13.2 Hz), 159.16, 150.77, 142.53 (t, ^3^J_C-F_ = 9.5 Hz), 135.82, 131.18, 129.17, 128.34, 124.45, 115.28, 112.36 (d, ^2^J_C-F_ = 24.7 Hz), 112.09, 111.94, 107.07, 103.25, 102.51 (t, ^2^J_C-F_ = 25.6 Hz), 55.92, 51.49, 48.60, 47.54, 46.13, 42.93, 37.76, 37.15, 31.13. HRMS (ESI) calculated for C_33_H_33_F_2_N_5_O_6_ [M+H]^+^ 634.2472, found 634.2477.

##### (S)-3-(3,5-difluorophenyl)-2-(2-(4-(2-(5-fluoro-1H-indol-3-yl)acetyl)-2-oxopiperazin-1-yl)acetamido)-N-(4-methoxyphenyl)-N-methylpropanamide (**F_2_-7f**)

White solid. Yield: 71%. Purity: 96%. Mp: 86–88 °C. ^1^H NMR (600 MHz, DMSO-d_6_) δ 10.99 (d, J = 10.8 Hz, 1H, NH), 8.39 (d, J = 7.8 Hz, 1H, NH), 7.37–7.27 (m, 3H, Ph-H), 7.23 (d, J = 7.4 Hz, 2H, Ph-H), 7.01 (d, J = 8.5 Hz, 3H, Ph-H), 6.91 (t, J = 9.0 Hz, 1H, Ph-H), 6.50 (d, J = 7.2 Hz, 2H, Ph-H), 4.46 (s, 1H, CH), 4.24–4.14 (m, 1H, CH), 4.07–3.98 (m, 1H, CH), 3.98–3.86 (m, 2H, CH_2_), 3.79 (s, 3H, OCH_3_), 3.76 (s, 2H, CH_2_), 3.70–3.57 (m, 1H, CH), 3.18 (d, J = 29.2 Hz, 2H, CH_2_), 3.13 (s, 3H, NCH_3_), 2.87 (dd, J = 13.4, 3.6 Hz, 1H, PhCH), 2.74–2.66 (m, 1H, PhCH). ^13^C NMR (150 MHz, DMSO-d_6_) δ 170.86 (C=O), 169.58 (C=O), 167.75 (C=O), 165.77 (C=O), 162.53 (dd, ^1^J_C-F_ = 246.0 Hz, ^3^J_C-F_ = 13.2 Hz), 159.18, 157.19 (d, ^1^J_C-F_ = 231.2 Hz), 142.53 (t, ^3^J_C-F_ = 9.4 Hz), 135.86, 133.30, 129.13, 128.02 (d, ^3^J_C-F_ = 10.2 Hz), 126.24, 115.30, 112.72 (d, ^3^J_C-F_ = 9.8 Hz), 112.35 (dd, ^2^J_C-F_ = 19.6 Hz), 109.61 (d, ^2^J_C-F_ = 26.0 Hz), 108.52, 103.95 (d, ^2^J_C-F_ = 23.3 Hz), 102.45 (t, ^2^J_C-F_ = 25.7 Hz), 55.93, 51.44, 47.48, 46.21, 42.95, 37.76, 37.25. HRMS (ESI) calculated for C_33_H_32_F_3_N_5_O_5_ [M+H]^+^ 636.2428, found 636.2427.

#### 3.1.3. General Procedure for the Synthesis of **7 (g-j)** and **F_2_-7 (g-j)**

Corresponding substituted indoleacetic acids (1.2 eq.) were first dissolved in 20 mL DCM, and then HATU (1.5 eq.) was added. The reaction mixture was stirred at 0 °C for 0.5 h. Then, the key intermediates **6** and **F_2_-6** (1 eq.) were added dropwise into the solution and DIEA (2 eq.) was added. The reaction mixture was restored to room temperature for 3 h. After monitoring the reaction by TLC, the excess solvent was evaporated under reduced pressure, and the residue was redissolved in water and extracted with DCM (3 × 20 mL). Subsequently, the organic layers were combined and washed with saturated sodium bicarbonate (3 × 20 mL), dried over anhydrous Na_2_SO_4_, filtered, and concentrated under reduced pressure to obtain the corresponding crude products. These products were purified by flash column chromatography to provide target compounds **7** (**g-j**) and **F_2_-7** (**g-j**).

##### (S)-2-(2-(4-(4-acetamidobenzoyl)-2-oxopiperazin-1-yl)acetamido)-N-(4-methoxyphenyl)-N-methyl-3-phenylpropanamide (**7g**)

White solid. Yield: 75%. Purity: 95%. Mp: 116–118 °C. ^1^H NMR (400 MHz, DMSO-*d*_6_) δ 10.14 (s, 1H, NH), 8.40 (d, *J* = 8.0 Hz, 1H, NH), 7.66 (d, *J* = 8.5 Hz, 2H, Ph-H), 7.39 (d, *J* = 8.4 Hz, 2H, Ph-H), 7.16 (dq, *J* = 12.8, 6.5 Hz, 5H, Ph-H), 6.98 (d, *J* = 8.9 Hz, 2H, Ph-H), 6.85 (d, *J* = 6.8 Hz, 2H, Ph-H), 4.47 (td, *J* = 8.7, 5.1 Hz, 1H, CH), 4.09 (s, 2H, CH_2_), 4.01–3.86 (m, 2H, CH_2_), 3.79 (s, 3H, OCH_3_), 3.73–3.54 (m, 2H, CH_2_), 3.22–3.13 (m, 2H, CH_2_), 3.11 (s, 3H, NCH_3_), 2.90–2.82 (m, 1H, PhCH), 2.65 (dd, *J* = 13.3, 9.4 Hz, 1H, PhCH), 2.07 (s, 3H, COCH_3_). ^13^C NMR (100 MHz, DMSO-*d*_6_) δ 171.39 (C=O), 169.11 (C=O), 169.07 (C=O), 167.55 (C=O), 165.21 (C=O), 159.04, 141.43, 137.96, 135.97, 130.81, 129.55, 129.33, 129.13, 128.69, 128.58, 126.85, 118.84, 115.18, 55.91, 51.84, 48.68, 37.79, 24.53. HRMS (ESI) calculated for C_32_H_35_N_5_O_6_ [M+H]^+^ 586.2660, found 586.2663.

##### (S)-N-(4-methoxyphenyl)-N-methyl-2-(2-(4-(4-(methylsulfonamido)benzoyl)-2-oxopiperazin-1-yl)acetamido)-3-phenylpropanamide (**7h**)

White solid. Yield: 80%. Purity: 99%. Mp: 106–108 °C. ^1^H NMR (400 MHz, DMSO-*d*_6_) δ 10.07 (s, 1H, NH), 8.40 (d, *J* = 8.0 Hz, 1H, NH), 7.43 (d, *J* = 8.3 Hz, 2H, Ph-H), 7.26 (d, *J* = 8.5 Hz, 2H, Ph-H), 7.16 (dq, *J* = 13.6, 6.8 Hz, 5H, Ph-H), 6.98 (d, *J* = 8.9 Hz, 2H, Ph-H), 6.85 (d, *J* = 6.8 Hz, 2H, Ph-H), 4.52–4.41 (m, 1H, CH), 4.09 (s, 2H, CH_2_), 4.01–3.86 (m, 2H, CH_2_), 3.79 (s, 3H, OCH_3_), 3.75–3.52 (m, 2H, CH_2_), 3.16 (d, *J* = 7.5 Hz, 2H, CH_2_), 3.11 (s, 3H, NCH_3_), 3.06 (s, 3H, SO_2_CH_3_), 2.90–2.81 (m, 1H, PhCH), 2.65 (dd, *J* = 13.4, 9.5 Hz, 1H, PhCH). ^13^C NMR (100 MHz, DMSO-*d*_6_) δ 171.40 (C=O), 168.89 (C=O), 167.56 (C=O), 165.17 (C=O), 159.03, 140.62, 137.95, 135.95, 130.10, 129.32, 129.23, 129.13, 128.59, 126.86, 118.85, 115.17, 55.90, 51.86, 48.68, 37.79. HRMS (ESI) calculated for C_31_H_35_N_5_O_7_S [M+H]^+^ 622.2330, found 622.2335.

##### (S)-N-(4-(4-(2-((1-((4-methoxyphenyl)(methyl)amino)-1-oxo-3-phenylpropan-2-yl)amino)-2-oxoethyl)-3-oxopiperazine-1-carbonyl)phenyl)cyclopropanecarboxamide (**7i**)

White solid. Yield: 69%. Purity: 99%. Mp: 108–110 °C. ^1^H NMR (400 MHz, DMSO-d_6_) δ 10.41 (s, 1H, NH), 8.42 (d, J = 8.0 Hz, 1H, NH), 7.68 (d, J = 8.5 Hz, 2H, Ph-H), 7.40 (d, J = 8.3 Hz, 2H, Ph-H), 7.16 (dq, J = 10.1, 5.1, 3.8 Hz, 5H, Ph-H), 6.98 (d, J = 8.8 Hz, 2H, Ph-H), 6.84 (d, J = 6.9 Hz, 2H, Ph-H), 4.46 (td, J = 8.8, 5.1 Hz, 1H, CH), 4.09 (s, 2H, CH_2_), 4.00–3.87 (m, 2H, CH_2_), 3.79 (s, 3H, OCH_3_), 3.72–3.53 (m, 2H, CH_2_), 3.16 (q, J = 7.8, 5.0 Hz, 2H, CH_2_), 3.11 (s, 3H, NCH_3_), 2.90–2.81 (m, 1H, PhCH), 2.65 (dd, J = 13.4, 9.5 Hz, 1H, PhCH), 1.79 (td, J = 11.2, 10.3, 5.1 Hz, 1H, CH), 0.82 (d, J = 5.2 Hz, 4H, CH_2_×2). ^13^C NMR (100 MHz, DMSO-d_6_) δ 172.47 (C=O), 171.40 (C=O), 169.08 (C=O), 167.56 (C=O), 165.21 (C=O), 159.03, 141.44, 137.96, 135.95, 129.44, 129.32, 129.13, 128.75, 128.59, 126.86, 118.83, 115.17, 55.90, 51.86, 48.67, 37.79, 15.11, 7.86. HRMS (ESI) calculated for C_34_H_37_N_5_O_6_ [M+H]^+^ 612.2817, found 636.2821.

##### (S)-N-(4-methoxyphenyl)-N-methyl-2-(2-(2-oxo-4-(4-(2,2,2-trifluoroacetamido)benzoyl)piperazin-1-yl)acetamido)-3-phenylpropanamide (**7j**)

White solid. Yield: 76%. Purity: 98%. Mp: 123–125 °C. ^1^H NMR (400 MHz, DMSO-d_6_) δ 11.45 (s, 1H, NH), 8.41 (d, J = 8.0 Hz, 1H, NH), 7.78 (d, J = 8.5 Hz, 2H, Ph-H), 7.51 (d, J = 8.2 Hz, 2H, Ph-H), 7.16 (dq, J = 12.3, 7.9, 7.2 Hz, 5H, Ph-H), 6.98 (d, J = 8.8 Hz, 2H, Ph-H), 6.85 (d, J = 6.8 Hz, 2H, Ph-H), 4.48 (dq, J = 8.3, 5.3 Hz, 1H, CH), 4.11 (s, 2H, CH_2_), 4.02–3.86 (m, 2H, CH_2_), 3.80 (s, 3H, OCH_3_), 3.72–3.46 (m, 2H, CH_2_), 3.22–3.14 (m, 2H, CH_2_), 3.11 (s, 3H, NCH_3_), 2.91–2.82 (m, 1H, PhCH), 2.66 (dd, J = 13.3, 9.5 Hz, 1H, PhCH). ^13^C NMR (100 MHz, DMSO-d_6_) δ 172.22 (C=O), 169.44 (C=O), 168.38 (C=O), 159.84 (C=O), 156.13 (C=O), 155.77, 139.11, 138.76, 136.74, 133.15, 130.13, 129.95, 129.50, 129.41, 127.68, 121.93, 118.40, 115.97, 56.70, 52.69, 49.47, 38.59, 38.55. HRMS (ESI) calculated for C_32_H_32_F_3_N_5_O_6_ [M+H]^+^ 640.2377, found 640.2378.

##### (S)-2-(2-(4-(4-acetamidobenzoyl)-2-oxopiperazin-1-yl)acetamido)-3-(3,5-difluorophenyl)-N-(4-methoxyphenyl)-N-methylpropanamide (**F_2_-7g**)

White solid. Yield: 79%. Purity: 95%. Mp: 105–106 °C. ^1^H NMR (400 MHz, DMSO-d_6_) δ 10.15 (s, 1H, NH), 8.43 (d, J = 8.0 Hz, 1H, NH), 7.67 (d, J = 8.6 Hz, 2H, Ph-H), 7.40 (d, J = 8.4 Hz, 2H, Ph-H), 7.25 (d, J = 8.7 Hz, 2H, Ph-H), 7.03 (d, J = 8.8 Hz, 3H, Ph-H), 6.50 (d, J = 6.5 Hz, 2H, Ph-H), 4.47 (td, J = 9.0, 4.5 Hz, 1H, CH), 4.10 (s, 2H, CH_2_), 4.02–3.89 (m, 2H, CH_2_), 3.80 (s, 3H, OCH_3_), 3.75–3.57 (m, 2H, CH_2_), 3.28–3.20 (m, 2H, CH_2_), 3.13 (s, 3H, NCH_3_), 2.88 (dd, J = 13.2, 4.0 Hz, 1H, PhCH), 2.70 (dt, J = 13.5, 7.0 Hz, 1H, PhCH), 2.07 (s, 3H, COCH_3_). ^13^C NMR (100 MHz, DMSO-d_6_) δ 171.67 (C=O), 169.97 (C=O), 169.88 (C=O), 168.51 (C=O), 166.02 (C=O), 163.30 (dd, ^1^J_C-F_ = 245.7 Hz, ^3^J_C-F_ = 13.3 Hz), 159.97, 143.40 (d, ^3^J_C-F_ = 9.4 Hz), 142.24, 136.62, 131.64, 130.28, 129.97, 129.51, 119.63, 116.09, 113.16 (d, ^2^J_C-F_ = 24.6 Hz), 103.16 (d, ^2^J_C-F_ = 25.7 Hz), 56.73, 52.30, 49.49, 38.57, 37.96, 25.34. HRMS (ESI) calculated for C_32_H_33_F_2_N_5_O_6_ [M+H]^+^ 622.2472, found 622.2476.

##### (S)-3-(3,5-difluorophenyl)-N-(4-methoxyphenyl)-N-methyl-2-(2-(4-(4-(methylsulfonamido)benzoyl)-2-oxopiperazin-1-yl)acetamido)propenamide (**F_2_-7h**)

White solid. Yield: 81%. Purity: 95%. Mp: 114–116 °C. ^1^H NMR (400 MHz, DMSO-d_6_) δ 10.08 (s, 1H, NH), 8.44 (d, J = 8.0 Hz, 1H, NH), 7.44 (d, J = 8.3 Hz, 2H, Ph-H), 7.26 (t, J = 8.3 Hz, 4H, Ph-H), 7.03 (d, J = 8.9 Hz, 3H, Ph-H), 6.50 (d, J = 6.6 Hz, 2H, Ph-H), 4.47 (td, J = 8.9, 4.5 Hz, 1H, CH), 4.10 (s, 2H, CH_2_), 3.93 (t, J = 9.7 Hz, 2H, CH_2_), 3.80 (s, 3H, OCH_3_), 3.74–3.56 (m, 2H, CH_2_), 3.28–3.20 (m, 2H, CH_2_), 3.13 (s, 3H, NCH_3_), 3.07 (s, 3H, SO_2_CH_3_), 2.88 (dd, J = 13.4, 4.0 Hz, 1H, PhCH), 2.70 (dd, J = 13.5, 9.6 Hz, 1H, PhCH). ^13^C NMR (100 MHz, DMSO-d_6_) δ 170.86 (C=O), 168.89 (C=O), 167.70 (C=O), 165.19 (C=O), 162.50 (dd, ^1^J_C-F_ = 245.8 Hz, ^3^J_C-F_ = 13.6 Hz), 159.16, 142.54 (d, ^3^J_C-F_ = 9.5 Hz), 140.63, 135.82, 131.35, 130.05, 129.17, 118.80, 118.09, 115.28, 112.33 (d, ^2^J_C-F_ = 25.0 Hz), 102.49 (t, ^2^J_C-F_ = 25.4 Hz), 55.92, 51.49, 48.70, 37.75, 37.15. HRMS (ESI) calculated for C_31_H_33_F_2_N_5_O_7_S [M+H]^+^ 658.2142, found 658.2146.

##### (S)-N-(4-(4-(2-((3-(3,5-difluorophenyl)-1-((4-methoxyphenyl)(methyl)amino)-1-oxopropan-2-yl)amino)-2-oxoethyl)-3-oxopiperazine-1-carbonyl)phenyl)cyclopropanecarboxamide (**F_2_-7i**)

White solid. Yield: 73%. Purity: 97%. Mp: 119–121 °C. ^1^H NMR (400 MHz, DMSO-d_6_) δ 10.40 (s, 1H, NH), 8.42 (d, J = 8.0 Hz, 1H, NH), 7.68 (d, J = 8.5 Hz, 2H, Ph-H), 7.40 (d, J = 8.4 Hz, 2H, Ph-H), 7.24 (d, J = 8.6 Hz, 2H, Ph-H), 7.03 (d, J = 8.8 Hz, 3H, Ph-H), 6.50 (d, J = 6.6 Hz, 2H, Ph-H), 4.47 (td, J = 8.9, 4.5 Hz, 1H, CH), 4.10 (s, 2H, CH_2_), 4.01–3.88 (m, 2H, CH_2_), 3.80 (s, 3H, OCH_3_), 3.75–3.59 (m, 2H, CH_2_), 3.23 (t, J = 4.9 Hz, 2H, CH_2_), 3.13 (s, 3H, NCH_3_), 2.88 (dd, J = 13.3, 4.0 Hz, 1H, PhCH), 2.75–2.65 (m, 1H, PhCH), 1.80 (p, J = 6.2 Hz, 1H, CH), 0.89–0.78 (m, 4H, CH_2_×2). ^13^C NMR (100 MHz, DMSO-d_6_) δ 172.49 (C=O), 170.86 (C=O), 169.08 (C=O), 167.70 (C=O), 165.22 (C=O), 162.49 (dd, ^1^J_C-F_ = 245.9 Hz, ^3^J_C-F_ = 13.4 Hz), 159.16, 142.54 (d, ^3^J_C-F_ = 9.6 Hz), 141.44, 135.81, 129.38, 129.16, 128.74, 118.82, 115.27, 112.35 (d, ^2^J_C-F_ = 24.7 Hz), 102.48 (t, ^2^J_C-F_ = 25.6 Hz), 55.91, 51.50, 48.69, 37.75, 37.16, 15.10, 7.87. HRMS (ESI) calculated for C_34_H_35_F_2_N_5_O_6_ [M+H]^+^ 648.2628, found 648.2627.

##### (S)-3-(3,5-difluorophenyl)-N-(4-methoxyphenyl)-N-methyl-2-(2-(2-oxo-4-(4-(2,2,2-trifluoroacetamido)benzoyl)piperazin-1-yl)acetamido)propenamide (**F_2_-7j**)

White solid. Yield: 78%. Purity: 96%. Mp: 104–106 °C. ^1^H NMR (400 MHz, DMSO-d_6_) δ 11.44 (s, 1H, NH), 8.43 (d, J = 8.0 Hz, 1H, NH), 7.78 (d, J = 8.5 Hz, 2H, Ph-H), 7.51 (d, J = 8.4 Hz, 2H, Ph-H), 7.25 (d, J = 8.6 Hz, 2H, Ph-H), 7.03 (d, J = 8.8 Hz, 3H, Ph-H), 6.50 (d, J = 6.8 Hz, 2H, Ph-H), 4.47 (td, J = 8.9, 4.4 Hz, 1H, CH), 4.11 (s, 2H, CH_2_), 4.01–3.89 (m, 2H, CH_2_), 3.80 (s, 3H, OCH_3_), 3.74–3.50 (m, 2H, CH_2_), 3.28–3.20 (m, 2H, CH_2_), 3.14 (s, 3H, NCH_3_), 2.88 (dd, J = 13.0, 3.5 Hz, 1H, PhCH), 2.76–2.65 (m, 1H, PhCH). ^13^C NMR (100 MHz, DMSO-d_6_) δ 170.87 (C=O), 168.64 (C=O), 167.71 (C=O), 162.49 (dd, ^1^J_C-F_ = 245.9 Hz, ^3^J_C-F_ = 13.4 Hz), 159.16 (C=O), 155.32, 154.95, 142.59 (d, ^3^J_C-F_ = 9.6 Hz), 138.31, 135.81, 129.17, 128.70, 121.11, 120.94, 117.58, 115.27, 114.71, 112.33 (d, ^2^J_C-F_ = 24.8 Hz), 102.49 (t, ^2^J_C-F_ = 25.9 Hz), 55.91, 51.51, 48.69, 37.76, 37.13. HRMS (ESI) calculated for C_32_H_30_F_5_N_5_O_6_ [M+H]^+^ 676.2189, found 676.2194.

### 3.2. In Vitro Anti-HIV Assay with MT-4 Cells

The protocol for the in vitro anti-HIV assay with MT-4 cells is provided in the Supplementary Materials.

### 3.3. Analysis of Binding to HIV-1 CA Proteins via Surface Plasmon Resonance

The protocol for analysis of binding to CA proteins via surface plasmon resonance is provided in the Supplementary Materials.

### 3.4. Molecular Dynamics Simulation

The protocol for the molecular dynamics simulation is provided in the Supplementary Materials.

### 3.5. In Silico ADMET Analysis

The protocol for the in silico ADMET analysis is provided in the Supplementary Materials.

### 3.6. Metabolic Stability in Human Liver Microsomes

The protocol for the metabolic stability assay using human liver microsomes is provided in the Supplementary Materials.

### 3.7. Metabolic Stability in Human Plasma

The protocol for the metabolic stability assay using human plasma is provided in the Supplementary Materials.

## 4. Conclusions

The multiple key roles of HIV-1 CA in the viral life cycle make it a novel and attractive target for drug design. However, due to the structural plasticity of CAs, especially between interprotomer pockets, rational drug design and optimization remain challenging. To address this issue, we thoroughly explored the crystallographic information available for the **PF74**-CA complex, focusing on key residues Gln67, Glu71, Tyr169, and Lys182 of the NTD-CTD interface as new binding sites for structural optimization to obtain compounds with improved antiviral activity and metabolic stability. In this study, we designed and synthesized 20 piperazinone phenylalanine derivatives with a terminal indole or benzene ring, preliminarily verifying the HIV-1 CA protein as the target of these compounds via SPR and MD simulation. According to our SAR studies, compounds with higher numbers of fluorine atoms at the terminal benzene of the two series showed the most potent anti-HIV-1 activity, as shown by **F_2_-7f** (EC_50_ = 5.89 μM) and **F_2_-7j** (EC_50_ = 13.74 μM). Mechanistically, our MD simulation results confirmed this finding, as compounds with terminal F atoms, such as **F_2_-7f**, had a 20% probability of forming a hydrogen bond with key residue Glu71. However, we acknowledge that the terminal electron-withdrawing F atoms introduce metabolic lability at the benzene ring, as our in silico analysis predicted.

Nevertheless, the anti-HIV-1 activities of the newly synthesized compounds were lower than that of **PF74** (EC_50_ = 0.75 μM), which might be due to a partial change in their binding modes to CA, resulting in the disappearance of two key hydrogen bonds between the compound and Asn57. Therefore, in our follow-up work, we will employ a conformational restriction strategy to maintain the conformation of the phenylalanine core to maintain its critical interactions. Notably, the newly synthesized compounds displayed significant anti-HIV-2 activity, with compound **7f** (EC_50_ = 4.52 μM) comparable to **PF74** (EC_50_ = 4.16 μM).

We used a previously reported workflow to predict the ADMET properties of representative compounds **7f** and **F_2_-7f**. Compared with **PF74**, **F_2_-7f** exhibited an improved oral non-CNS drug profile score but no significant improvements in metabolic stability. Moreover, the knowledge-based in silico prediction indicated that the compounds should not induce any genotoxicity and hepatotoxicity issues. Finally, we experimentally verified the in silico metabolic stability results utilizing HLMs and human plasma stability. Although lacking significant metabolic improvements, **F_2_-7f** represents a new chemotype with the potential for further modifications to improve anti-HIV potency and metabolic stability.

## Figures and Tables

**Table 1:** Anti-HIV activity and cytotoxicity of series I in MT-4 cells.

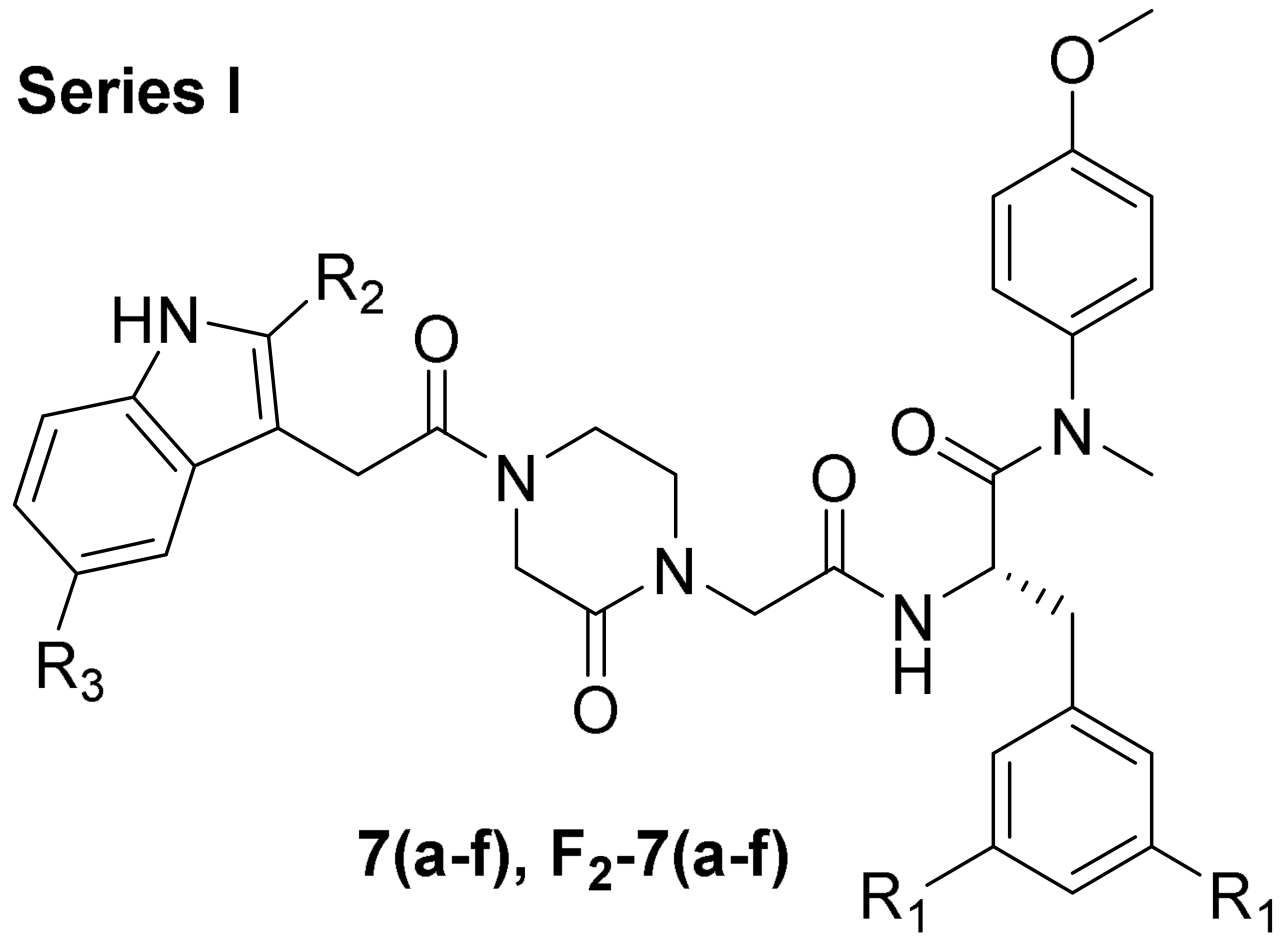								
Compound ID	R_1_	R_2_	R_3_	EC_50_^a^ (μM)		CC_50_^b^ (μM)	SI ^c^	
				III_B_	ROD		III_B_	ROD
**7a**	H	CH_3_	H	14.28 ± 0.76	7.74 ± 0.79	107.56 ± 3.26	7.73	13.90
**7b**	H	H	OCH_3_	14.58 ± 0.39	16.95 ± 3.58	104.21 ± 3.37	7.15	6.15
**7c**	H	H	Br	>16.37	>16.37	16.37 ± 3.44	<1	<1
**7d**	H	H	H	14.97 ± 0.24	4.89 ± 1.39	112.41 ± 3.22	7.51	22.99
**7e**	H	H	OH	198.10 ± 9.74	186.48 ± 4.42	>209.29	>1.06	>1.12
**7f**	H	H	F	21.81 ± 4.81	4.52 ± 0.87	90.06 ± 15.72	4.13	19.92
**F_2_-7a**	F	CH_3_	H	20.83 ± 5.09	19.66 ± 6.59	76.85 ± 19.98	3.69	3.91
**F_2_-7b**	F	H	OCH_3_	>15.94	>15.94	15.94 ± 3.83	<1	<1
**F_2_-7c**	F	H	Br	>3.15	>3.15	3.15 ± 0.40	<1	<1
**F_2_-7d**	F	H	H	29.37 ± 7.03	17.61 ± 9.14	57.84 ± 21.81	1.97	3.28
**F_2_-7e**	F	H	OH	>131.51	≥49.55	131.51 ± 32.96	<1	≤2.65
**F_2_-7f**	F	H	F	5.89 ± 2.03	5.07 ± 0.63	16.36 ± 3.38	2.78	3.23
**PF74**				0.75 ± 0.33	4.16 ± 2.02	32.27 ± 2.94	43.03	7.76

^a^ EC_50_: concentration of the test compound to attain 50% protection against HIV-induced cytotoxicity, determined by the MTT assay. ^b^ CC_50_: concentration of the test compound to reduce the viability of mock-infected cell cultures by 50%, determined by the MTT assay; values were averaged from at least three independent experiments. ^c^ SI: selectivity index, the ratio of CC_50_/EC_50_.

**Table 2:** Anti-HIV activity and cytotoxicity of series II in MT-4 cells.

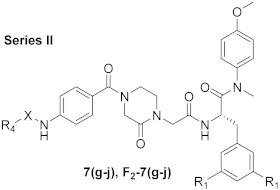								
Compound ID	R_1_	X	R_4_	EC_50_^a^ (µM)		CC_50_^b^ (µM)	SI ^c^	
				III_B_	ROD		III_B_	ROD
**7g**	H	CO	CH_3_	203.14 ± 10.98	184.07 ± 19.33	>213.43	>1.05	>1.16
**7h**	H	SO2	CH_3_	192.39 ± 3.17	>201.06	>201.06	>1.05	ND
**7i**	H	CO	cyclopropyl	>167.50	15.02 ± 2.27	167.50 ± 19.81	<1	11.15
**7j**	H	CO	CF_3_	157.50 ± 6.77	14.95 ± 4.83	>195.42	>1.24	>13.08
**F_2_-7g**	F	CO	CH_3_	177.40 ± 16.55	192.14 ± 4.57	196.03 ± 6.80	1.11	1.02
**F_2_-7h**	F	SO2	CH_3_	160.53 ± 15.43	183.40 ± 2.77	>190.06	>1.18	>1.04
**F_2_-7i**	F	CO	cyclopropyl	14.36 ± 1.31	15.10 ± 0.68	88.56 ± 11.27	6.17	5.87
**F_2_-7j**	F	CO	CF_3_	13.74 ± 0.70	16.55 ± 6.13	150.38 ± 25.46	10.95	9.09
**PF74**				0.75 ± 0.33	4.16 ± 2.02	32.27 ± 2.94	43.03	7.76

^a^ EC_50_: concentration of the test compound to attain 50% protection against HIV-induced cytotoxicity, determined by the MTT assay. ^b^ CC_50_: concentration of the test compound to reduce the viability of mock-infected cell cultures by 50%, determined by the MTT assay; values were averaged from at least three independent experiments. ^c^ SI: selectivity index, the ratio of CC_50_/EC_50_.

**Table 3:** SPR results of **F_2_-7f** and **PF74** binding to monomeric and hexameric CA constructs.

Compound ID	K_D_^a^ (μM)		Ratio ^b^	k_off_^c^ (s^−1^)
	Monomer	Hexamer		Hexamer
**F_2_-7f**	16.063 ± 1.316	7.203 ± 1.101	2.230	0.151 ± 0.028
**PF74**	3.410 ± 1.310	0.159 ± 0.041	21.446	0.0177 ± 0.0004

^a^ All values represent the average response from at least three replicates. Error bars indicate standard deviation (SD); ^b^ Ratio = K_D_^Monomer^/K_D_^Hexamer^. ^c^ k_off_ = off-rate measured for HIV-1 CA hexamer.

**Table 4:** Metabolic stability assay in human liver microsomes.

Sample	HLM (Final Concentration of 0.5 mg Protein/mL)					
	R^2 a^	T_1/2_^b^ (min)	CL_int(mic)_^c^ (μL/min/mg)	CL_int(liver)_^d^ (mL/min/kg)	Remaining (T = 60 min)	Remaining (NCF ^e^ = 60 min)
**F_2_-7f**	1.0000	0.5	2759.1	2483.2	0.0%	103.5%
**PF74**	1.0000	0.5	2862.5	2576.2	0.0%	112.6%
**Testosterone**	0.9982	16.7	82.8	74.5	7.9%	90.7%
**Diclofenac**	0.9947	3.7	372.0	334.8	0.0%	96.7%
**Propafenone**	0.9350	5.0	279.5	251.5	0.0%	93.6%

^a^ R^2^ is the correlation coefficient of the linear regression for determination of the kinetic constant. ^b^ T_1/2_ is half-life, and CLint(mic) is the intrinsic clearance. ^c^ CL_int(mic)_ = (0.693/half-life)/mg microsome protein per mL. ^d^ CL_int(liver)_ = CL_int(mic)_ × mg microsomal protein/g liver weight × g liver weight/kg body weight. ^e^ NCF: no cofactor. No NADPH regenerating system was added to the NCF sample (replaced by buffer) during the 60 min incubation. Less than 60% remaining indicates that a non-NADPH-dependent reaction occurred.

**Table 5:** Result of human plasma stability assay.

Compound ID	Batch	Time Point (min)	% Remaining ^a^	T_1/2_ (min)
			Human	Human
**F_2_-7f**	/	0	100.0	>289.1
		10	98.0	
		30	97.8	
		60	90.9	
		120	86.9	
**PF74**	/	0	100.0	>289.1
		10	84.7	
		30	81.7	
		60	80.7	
		120	85.2	
**Propantheline bromide**	ODR-4547	0	100.0	11.2
		10	56.5	
		30	19.4	
		60	1.9	
		120	0.1	

^a^ % remaining = 100 × (PAR at appointed incubation time/PAR at time T_0_). PAR is the peak area ratio of test compound to internal standard. Accuracy should be within 80−120% of the indicated value.

## Data Availability

Not applicable.

## Supplementary Materials

The following supporting information can be downloaded at https://www.mdpi.com/article/10.3390/molecules27238415/s1. References [29,30,32,34,35,37,42,43,44,45,46,47,48,49,50,51,52,53,54,55,56,57] are cited in the Supplementary Materials.

## Sample Availability

Samples of the compounds are available from the authors.
