# Supplementary material for: Discovery and Mechanistic Investigation of Piperazinone Phenylalanine Derivatives with Terminal Indole or Benzene Ring as Novel HIV-1 Capsid Modulators
Source: Molecules. 2022 Dec 1;27(23):8415. doi: 10.3390/molecules27238415 (PMC9739877; doi:10.3390/molecules27238415)
Supplement: Supplementary file 1 [file molecules-27-08415-s001.zip › molecules-2047387-supplementary.pdf]

Discovery and Mechanistic Investigation of Piperazinone Phenylalanine Derivatives with Terminal Indole or Benzene Ring as Novel HIV-1 Capsid Modulators

Shujing Xu [1], Lin Sun [1,2], Waleed A. Zalloum [3], Tianguang Huang [1], Xujie Zhang [1], Dang Ding [1], Xiaoyu Shao [1], Xiangyi Jiang [1], Fabao Zhao [1], Simon Cocklin [4], Erik De Clercq [5], Christophe Pannecouque [5,*], Alexej Dick [6,*], Xinyong Liu [1,*] and Peng Zhan [1,*]

[1] Key Laboratory of Chemical Biology (Ministry of Education), Department of Medicinal Chemistry, School of Pharmaceutical Sciences, Shandong University, 44 West Culture Road, Jinan 250012, China
[2] Department of Pharmacy, Qilu Hospital of Shandong University, Jinan 250012, China
[3] Department of Pharmacy, Faculty of Health Science, American University of Madaba, P.O. Box 2882, Amman 11821, Jordan
[4] Specifica Inc., The Santa Fe Railyard, 1607 Alcaldesa Street, Santa Fe, NM 87501, USA
[5] Laboratory of Virology and Chemotherapy, Rega Institute for Medical Research, K.U. Leuven, Herestraat 49 Postbus 1043 (09.A097), B-3000 Leuven, Belgium
[6] Department of Biochemistry & Molecular Biology, Drexel University College of Medicine, Philadelphia, PA 19102, USA
* Correspondence: christophe.pannecouque@kuleuven.be (C.P.); ad3474@drexel.edu (A.D.); xinyongl@sdu.edu.cn (X.L.); zhanpeng1982@sdu.edu.cn (P.Z.)

Table of Contents

I. Procedure for the Synthesis of Intermediates

II. *In vitro* anti-HIV Assay in MT-4 Cells

III. Binding Analysis to HIV-1 CA Proteins *via* Surface Plasmon Resonance

IV. Molecular Dynamics Simulation

V. *In silico* ADMET Analysis

VI. Metabolic Stability in Human Liver Microsomes

VII. Metabolic Stability in Human Plasma

VIII. ^{1}H-NMR, ^{13}C-NMR and HRMS Spectra for Target Compounds

IX. References

I. Procedure for the Synthesis of Intermediates [1,2]

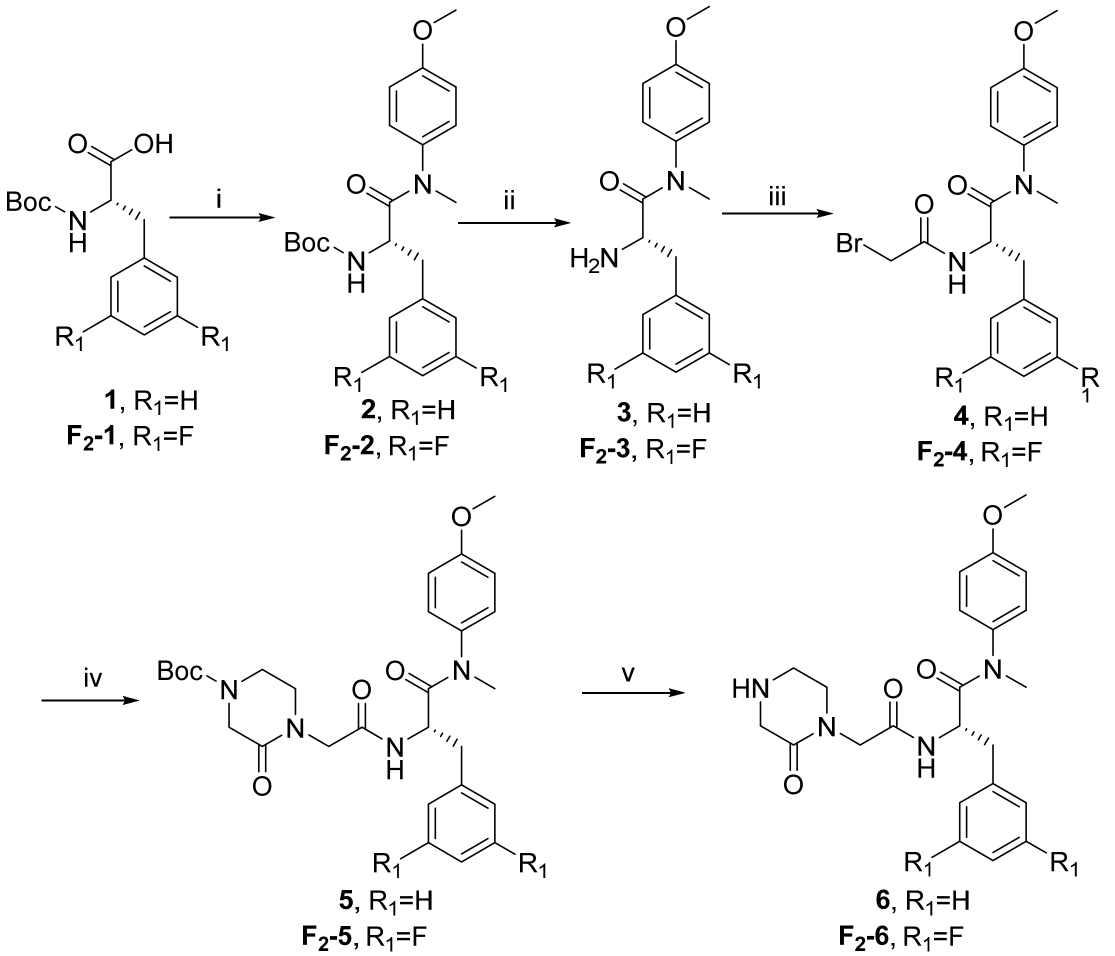

Scheme 1. Reagents and conditions:(i) 4-Methoxy-*N*-methylaniline, HATU, DIEA, 0°C to r.t.; (ii) TFA, DCM, r.t.; (iii) Bromoacetic Acid, HATU, DIEA, 0°C to r.t.; (iv) 1-Boc-3-oxopiperazine, Cs_2CO_3, DMF, 45°C; (v) TFA, DCM, r.t..

1.1 General procedure for the synthesis of 2 and F_2-2.

To a solution of (*tert*-butoxycarbonyl)-*L*-phenylalanine or (*tert*-butoxycarbonyl)-3,5-difluoro-*L*-phenylalanine (1.5 eq.) in 20 mL dichloromethane was added HATU (1.5 eq.) at 0ºC, and the mixture was stirred for 0.5 h. Subsequently, DIEA (3 eq.) and 4-methoxy-*N*-methylaniline (1 eq.) were added to the mixture and then stirred at room temperature for another 6 h (monitored by TLC). The resulting mixture was evaporated under reduced pressure, and the residue was initially washed by 1N HCl and extracted with ethyl acetate (3 × 20 mL). Then, the combined organic layer was washed with

saturated sodium bicarbonate (3 × 20 mL), dried over anhydrous Na_2SO_4, filtered, and concentrated under reduced pressure to afford a corresponding crude product, purified by flash column chromatography to afford intermediate **2** and **F_2-2**.

1.1.1 tert-butyl (*S*)-(1-((4-methoxyphenyl)(methyl)amino)-1-oxo-3-phenylpropan-2-yl)carbamate (2)

Yellow oil, yield: 85%. ^{1}H NMR (400 MHz, DMSO-d6) δ 7.22 (d, *J* = 8.3 Hz, 2H, Ph-H), 7.20 – 7.11 (m, 3H, Ph-H), 7.09 (d, *J* = 8.2 Hz, 1H, NH), 7.03 (d, *J* = 8.6 Hz, 2H, Ph-H), 6.79 (d, *J* = 7.3 Hz, 2H, Ph-H), 4.27 – 4.06 (m, 1H, CH), 3.81 (s, 3H, OCH_3), 3.13 (s, 3H, NCH_3), 2.75 (dd, *J* = 13.4, 3.8 Hz, 1H, PhCH), 2.61 (dd, *J* = 13.3, 10.3 Hz, 1H, PhCH), 1.30 (s, 9H, $C(CH_3)_3$). ^{13}C NMR (100 MHz, DMSO-d6) δ 172.22 (C=O), 158.98, 155.75 (C=O), 138.53, 136.12, 129.28, 128.47, 126.70, 115.21, 78.33, 55.94, 53.55, 37.86, 37.07, 28.65. ESI-MS: m/z 385.4 $(M+1)^+$, 407.5 $(M+23)^+$. $C_{22}H_{28}N_2O_4$ [384.48].

1.1.2 tert-butyl (*S*)-(3-(3,5-difluorophenyl)-1-((4-methoxyphenyl)(methyl)amino)-1-oxopropan-2-yl)carbamate (F_2-2)

Yellow oil, yield: 90%. ^{1}H NMR (400 MHz, DMSO-*d*6) δ 7.31 (d, *J* = 8.6 Hz, 2H, Ph-H), 7.08 (dd, *J* = 12.9, 9.0 Hz, 3H, Ph-H×2, NH), 7.00 (t, *J* = 9.4 Hz, 1H, Ph-H), 6.44 (d, *J* = 7.1 Hz, 2H, Ph-H), 4.20 – 4.10 (m, 1H, CH), 3.81 (s, 3H, OCH_3), 3.14 (s, 3H, NCH_3), 2.83 – 2.73 (m, 1H, PhCH), 2.71 – 2.61 (m, 1H, PhCH), 1.29 (s, 9H, $C(CH_3)$). ^{13}C NMR (150 MHz, DMSO-*d*6) δ 171.56, 162.47 (dd, $^1J_{C-F}$ = 245.8 Hz, $^3J_{C-F}$ =13.7 Hz), 159.18, 155.59, 143.14 (t, $^3J_{C-F}$ = 9.5 Hz), 136.06, 115.37, 112.32 (d, $^2J_{C-F}$ = 23.1 Hz), 102.23 (t, $^2J_{C-F}$ = 25.4 Hz), 78.52, 55.97, 53.10, 37.81, 36.85, 28.58. ESI-MS: m/z 421.07 $(M+1)^+$, 443.17 $(M+23)^+$. $C_{22}H_{26}F_2N_2O_4$ [420.46].

1.2 General procedure for the synthesis of 3 and F_2-3.

Trifluoroacetic acid (5.0 eq.) was added dropwise to the corresponding substituted intermediate **2** and **F_2-2** (1.0 eq.) in 30 mL dichloromethane and stirred at room temperature for 1 h (monitored by TLC). Then, the resulting mixture solution was alkalized to pH ~7 with saturated sodium bicarbonate solution and then extracted with

dichloromethane (40 mL). Then, the combined organic layer was washed with saturated sodium bicarbonate (3 × 20 mL), dried over anhydrous Na_2SO_4, filtered, and concentrated under reduced pressure to afford corresponding crude products **3** and **F_2-3**.

1.2.1 (*S*)-2-amino-*N*-(4-methoxyphenyl)-*N*-methyl-3-phenylpropanamide (3)

Yellow oil, yield: 82%. ^1H NMR (400 MHz, DMSO-d6) δ 7.29 – 7.13 (m, 3H, Ph-H), 7.03 – 6.75 (m, 6H, Ph-H), 3.77 (s, 3H, OCH_3), 3.44 – 3.35 (m, 1H, CH), 3.06 (s, 3H, NCH_3), 2.75 (dd, J = 12.8, 6.7 Hz, 1H, PhCH), 2.45 (dd, J = 12.9, 7.1 Hz, 1H, PhCH), 1.87 (s, 2H, NH_2). ^{13}C NMR (100 MHz, DMSO-d6) δ 174.89 (C=O), 158.75, 139.00, 136.35, 129.51, 128.93, 128.47, 126.55, 115.04, 55.85, 53.35, 42.19, 37.45. ESI-MS: m/z 285.05 $(M+1)^+$. $C_{17}H_{20}N_2O_2$ [284.36].

1.2.2 (*S*)-2-amino-3-(3,5-difluorophenyl)-*N*-(4-methoxyphenyl)-*N*-methylpropanamide (F_2-3)

Yellow oil, yield: 86%. ^1H NMR (400 MHz, DMSO-*d6*) δ 7.07 (d, J = 8.4 Hz, 2H, Ph-H), 7.02 (d, J = 9.5 Hz, 1H, Ph-H), 6.97 (d, J = 8.9 Hz, 2H, Ph-H), 6.57 (d, J = 6.9 Hz, 2H, Ph-H), 3.78 (s, 3H, OCH_3), 3.38 – 3.33 (m, 1H, CH), 3.09 (s, 3H, NCH_3), 2.74 (dd, J = 13.0, 5.8 Hz, 1H, PhCH), 2.54 – 2.45 (m, 1H, PhCH), 1.82 (s, 2H, NH_2). ^{13}C NMR (150 MHz, DMSO-*d6*) δ 174.57, 162.50 (dd, $^1J_{C-F}$ = 245.5 Hz, $^3J_{C-F}$ =13.3 Hz), 158.96, 143.82 (t, $^3J_{C-F}$ = 9.3 Hz), 136.31, 128.95, 115.21, 112.50 (d, $^2J_{C-F}$ = 24.3 Hz), 101.96 (t, $^2J_{C-F}$ = 25.8 Hz), 55.91, 52.99, 41.27, 37.48. ESI-MS: m/z 321.11 $(M+1)^+$, m/z 343.25 $(M+23)^+$. $C_{17}H_{18}F_2N_2O_2$ [320.34].

1.3 General procedure for the synthesis of 4 and F_2-4

Bromoacetic acid (1.2 eq.) and HATU (1.5 eq.) were mixed in 15 mL dichloromethane and stirred in an ice bath for 0.5 h. Then, the corresponding substituted intermediate **3** and **F_2-3** (1 eq.) and DIEA (2 eq.) were slowly added to the above solution at 0 ºC. The reaction system was then stirred at room temperature for an additional 0.5 h (monitored by TLC). The resulting mixture was evaporated under reduced pressure, and the residue was initially washed by 1N HCl and extracted with ethyl acetate (3 × 20 mL). Then, the

combined organic layer was washed with saturated sodium bicarbonate (3 × 20 mL), dried over anhydrous Na_2SO_4, filtered, and concentrated under reduced pressure to afford a corresponding crude product, purified by flash column chromatography to afford intermediates **4** and **F_2-4**.

1.3.1 (*S*)-2-(2-bromoacetamido)-*N*-(4-methoxyphenyl)-*N*-methyl-3-phenylpropanamide (4)

White oil, yield: 70%. ^{1}H NMR (600 MHz, DMSO-*d*$_6$) δ 8.62 (d, *J* = 7.9 Hz, 1H, NH), 7.22 – 7.16 (m, 3H, Ph-H), 7.09 – 7.01 (m, 2H, Ph-H), 6.96 (d, *J* = 9.0 Hz, 2H, Ph-H), 6.90 – 6.86 (m, 2H, Ph-H), 4.44 (td, *J* = 8.3, 5.7 Hz, 1H, CH), 3.84 – 3.80 (m, 2H, CH_2), 3.79 (s, 3H, OCH_3), 3.10 (s, 3H, NCH_3), 2.87 (dd, *J* = 13.5, 5.5 Hz, 1H, PhCH), 2.65 (dd, *J* = 13.5, 8.7 Hz, 1H, PhCH). ^{13}C NMR (150 MHz, DMSO-*d*$_6$) δ 171.06 (C=O), 165.90 (C=O), 159.08, 137.63, 135.89, 129.37, 129.06, 128.61, 126.95, 115.18, 55.92, 52.11, 37.95, 37.78, 29.52. ESI-MS: m/z 405.4 $(M+1)^+$. $C_{19}H_{21}BrN_2O_3$ [405.29].

1.3.2 (*S*)-2-(2-bromoacetamido)-3-(3,5-difluorophenyl)-*N*-(4-methoxyphenyl)-*N*-methylpropanamide (F_2-4)

White solid, yield: 86%. ^{1}H NMR (400 MHz, DMSO-*d*$_6$) δ 8.70 (d, *J* = 8.0 Hz, 1H, NH), 7.22 (d, *J* = 8.6 Hz, 2H, Ph-H), 7.02 (d, *J* = 8.7 Hz, 3H, Ph-H), 6.52 (d, *J* = 6.6 Hz, 2H, Ph-H), 4.43 (td, *J* = 8.7, 4.6 Hz, 1H, CH), 3.81 (s, 2H, CH_2), 3.80 (s, 3H, OCH_3), 3.13 (s, 3H, NCH_3), 2.89 (dd, *J* = 13.7, 4.5 Hz, 1H, PhCH), 2.70 (dt, *J* = 13.6, 9.3 Hz, 1H, PhCH). ^{13}C NMR (150 MHz, DMSO-*d*$_6$) δ 170.57 (C=O), 166.05 (C=O), 162.55 (dd, $^1J_{C\text{-}F}$ = 246.0 Hz, $^3J_{C\text{-}F}$ = 13.3 Hz), 159.24, 142.21 (t, $^3J_{C\text{-}F}$ = 9.5 Hz), 135.79, 129.10, 112.42 (dd, $^2J_{C\text{-}F}$ = 19.8 Hz), 102.50 (t, $^2J_{C\text{-}F}$ = 25.6 Hz), 55.95, 37.76, 37.31, 29.33. ESI-MS: m/z 443.15 $(M+2)^+$, m/z 463.16 $(M-1+23)^+$. $C_{17}H_{18}F_2N_2O_2$ [441.27].

1.4 General procedure for the synthesis of 5 and F_2-5

Under ice bath, the corresponding substituted key intermediate **4** and **F_2-4** (1 eq.), 1-Boc-3-oxopiperazine (1.2 eq.), Cs_2CO_3 (2 eq.) were dissolved in the solution of DMF (6 mL). The resulting mixture was then stirred at 40°C (monitored by TLC). Then the reaction mixture was extracted with ethyl acetate (20 mL), and the combined organic

phase was washed with saturated NaCl solution (3 × 20 mL), dried over anhydrous Na_2SO_4, filtered, and concentrated under reduced pressure to give the corresponding crude product which was purified by flash column chromatography to afford products **5** and **F_2-5**.

1.4.1 *tert*-butyl (*S*)-4-(2-((1-((4-methoxyphenyl)(methyl)amino)-1-oxo-3-phenylpropan-2-yl)amino)-2-oxoethyl)-3-oxopiperazine-1-carboxylate (5)

White solid, yield: 58%. ^{1}H NMR (600 MHz, DMSO-*d6*) δ 8.41 (d, *J* = 8.1 Hz, 1H, NH), 7.21 – 7.09 (m, 5H, Ph), 6.98 (d, *J* = 7.7 Hz, 2H, Ph), 6.86 (d, *J* = 6.5 Hz, 2H, Ph), 4.48 (td, *J* = 9.1, 8.6, 5.1 Hz, 1H, CH), 4.00 – 3.88 (m, 4H, CH_2×2), 3.79 (s, 3H, OCH_3), 3.48 (dt, *J* = 11.2, 5.4 Hz, 4H, CH_2×2), 3.11 (s, 3H, NCH_3), 2.86 (dd, *J* = 13.6, 5.0 Hz, 1H, PhCH), 2.66 (dd, *J* = 13.5, 9.3 Hz, 1H, PhCH), 1.42 (s, 9H, $C(CH_3)_3$). ^{13}C NMR (150 MHz, DMSO-*d6*) δ 171.38 (C=O), 167.58 (C=O), 159.00, 153.81 (C=O), 153.57 (C=O), 137.91, 135.92, 129.31, 129.08, 128.54, 126.82, 115.11, 80.03, 79.90, 55.84, 51.81, 48.68, 46.99, 40.33, 37.81, 37.73, 28.40. ESI-MS: m/z 523.09 $(M-1)^-$. $C_{28}H_{36}N_4O_6$ [524.62].

1.4.2 tert-butyl (*S*)-4-(2-((3-(3,5-difluorophenyl)-1-((4-methoxyphenyl)(methyl)amino)-1-oxopropan-2-yl)amino)-2-oxoethyl)-3-oxopiperazine-1-carboxylate (F_2-5)

White solid, yield: 88%. ^{1}H NMR (400 MHz, DMSO-*d*6) δ 8.42 (d, *J* = 8.0 Hz, 1H, NH), 7.24 (d, *J* = 8.6 Hz, 2H, Ph-H), 7.01 (dd, *J* = 8.9, 4.6 Hz, 3H, Ph-H), 6.49 (d, *J* = 6.6 Hz, 2H, Ph-H), 4.46 (td, *J* = 8.9, 4.4 Hz, 1H, CH), 3.98 – 3.90 (m, 2H, CH_2), 3.88 (d, *J* = 8.8 Hz, 2H, CH_2), 3.79 (s, 3H, OCH_3), 3.50 (t, *J* = 4.8 Hz, 2H, CH_2), 3.16 (t, *J* = 5.3 Hz, 2H, CH_2), 3.13 (s, 3H, NCH_3), 2.87 (dd, *J* = 13.6, 4.2 Hz, 1H, PhCH), 2.69 (dd, *J* = 13.5, 9.6 Hz, 1H, PhCH), 1.41 (s, 9H, $C(CH_3)_3$). ^{13}C NMR (150 MHz, DMSO-*d*6) δ 170.85, 167.70, 167.63, 162.53 (dd, $^1J_{C\text{-}F}$ = 245.9 Hz, $^3J_{C\text{-}F}$ = 13.5 Hz), 159.19, 153.63, 142.55 (t, $^3J_{C\text{-}F}$ = 9.6 Hz), 135.86, 115.30, 112.36 (d, $^2J_{C\text{-}F}$ = 24.8 Hz), 102.44 (t, $^2J_{C\text{-}F}$ = 26.0 Hz), 80.09, 55.93, 51.42, 51.33, 48.77, 48.74, 47.12, 37.76, 37.26, 28.45. ESI-MS: m/z 561.00 $(M+1)^+$, 583.19 $(M+23)^+$, 599.16 $(M+39)^+$, 559.22 $(M-1)^-$. $C_{28}H_{34}F_2N_4O_6$ [560.60].

1.5 General procedure for the synthesis of 6 and F_2-6

Trifluoroacetic acid (5.0 eq.) was added dropwise to corresponding substituted intermediate **5** and **F_2-5** (1.0 eq.) in 30 mL dichloromethane and stirred at room temperature for 1 h (monitored by TLC). Then, the resulting mixture solution was alkalized to pH ~7 with saturated sodium bicarbonate solution and then extracted with dichloromethane (40 mL). Then, the combined organic layer was washed with saturated sodium bicarbonate (3 × 20 mL), dried over anhydrous Na_2SO_4, filtered, and concentrated under reduced pressure to afford a corresponding crude product purified by flash column chromatography to afford products **6** and **F_2-6**.

1.5.1 (*S*)-*N*-(4-methoxyphenyl)-*N*-methyl-2-(2-(2-oxopiperazin-1-yl)acetamido)-3-phenylpropanamide (6)

Yellow oil, yield: 72%.^{1}H NMR (600 MHz, DMSO-d_6) δ 8.23 (d, J = 8.0 Hz, 1H, NH), 7.22 – 7.08 (m, 5H, Ph-H), 6.97 (d, J = 8.9 Hz, 2H, Ph-H), 6.85 (d, J = 6.5 Hz, 2H, Ph-H), 4.46 (td, J = 8.7, 5.2 Hz, 1H, CH), 3.87 (s, 2H, CH_2), 3.80 (s, 3H, OCH_3), 3.22 (s, 2H, CH_2), 3.11 (s, 3H, NCH_3), 3.04 (t, J = 5.5 Hz, 2H, CH_2), 2.86 (dd, J = 13.7, 5.1 Hz, 2H, CH_2), 2.82 (d, J = 5.8 Hz, 1H, PhCH), 2.68 (dd, J = 13.7, 9.2 Hz, 1H, PhCH), 1.24 (s, 1H, NH). ^{13}C NMR (100 MHz, DMSO-d_6) δ 171.46 (C=O), 167.90 (C=O), 159.03 (C=O), 137.97, 136.00, 129.34, 129.13, 128.57, 126.86, 115.16, 55.91, 51.78, 50.06, 48.61, 48.58, 42.80, 37.79. HRMS: m/z 425.2162 $(M+1)^+$. $C_{23}H_{28}N_4O_4$ [424.2111].

1.5.2 (*S*)-3-(3,5-difluorophenyl)-*N*-(4-methoxyphenyl)-*N*-methyl-2-(2-(2-oxopiperazin-1-yl)acetamido)propenamide (F_2-6)

Yellow oil, yield: 85%. ^{1}H NMR (400 MHz, DMSO-d_6) δ 8.31 (d, J = 8.0 Hz, 1H, NH), 7.26 (d, J = 8.5 Hz, 2H, Ph-H), 7.03 (d, J = 8.6 Hz, 3H, Ph-H), 6.50 (d, J = 6.8 Hz, 2H, Ph-H), 4.46 (td, J = 9.1, 4.2 Hz, 1H, CH), 3.87 (s, 2H, CH_2), 3.80 (s, 3H, OCH_3), 3.21 (s, 2H, CH_2), 3.13 (s, 3H, NCH_3), 3.04 (t, J = 5.2 Hz, 2H, CH_2), 2.86 (dd, J = 13.7, 4.1 Hz, 1H, PhCH), 2.84 – 2.77 (m, 2H, CH_2), 2.70 (dd, J = 13.7, 9.8 Hz, 1H, PhCH), 1.24 (s, 1H, NH). ^{13}C NMR (100 MHz, DMSO-d_6) δ 170.94 (C=O), 168.22 (C=O), 168.03 (C=O), 162.51 (dd, $^1J_{C\text{-}F}$ = 245.8 Hz, $^3J_{C\text{-}F}$ = 13.3 Hz), 159.18, 142.60 (t, $^3J_{C\text{-}F}$ = 9.4 Hz), 135.89, 112.39 (dd, $^2J_{C\text{-}F}$ = 19.8 Hz), 102.44 (t, $^2J_{C\text{-}F}$ = 25.7 Hz), 55.94, 51.43, 50.24,

48.81, 48.66, 42.88, 37.75, 37.16. HRMS: m/z 461.1993 $(M+1)^+$, 921.3886 $(2M+1)^+$. $C_{23}H_{26}F_2N_4O_4$ [460.1922].

II. *In vitro* anti-HIV Assay in MT-4 Cells

Evaluation of the antiviral activity of the compounds against HIV in MT-4 cells was performed using the MTT assay as described below. Stock solutions (10 × final concentration) of test compounds were added in 25 μL volumes to two series of triplicate wells to allow simultaneous evaluation of their effects on mock- and HIV-infected cells at the beginning of each experiment. Serial 5-fold dilutions of test compounds were made directly in flat-bottomed 96-well microtiter trays using a Biomek 3000 robot (Beckman Instruments, Fullerton, CA). Untreated HIV- and mock-infected cell samples were included as controls. HIV stock (50 μL) at 100-300 $CCID_{50}$ (50 % cell culture infectious doses) or culture medium was added to either the infected or mock-infected wells of the microtiter tray. Mock-infected cells were used to evaluate the effects of the test compound on uninfected cells to assess the test compounds' cytotoxicity. Exponentially growing MT-4 cells were centrifuged for 5 minutes at 220 g, and the supernatant was discarded. The MT-4 cells were resuspended at 6×10^5 cells/mL, and 50 μL volumes were transferred to the microtiter tray wells. Five days after infection, the viability of mock-and HIV-infected cells was examined spectrophotometrically using the MTT assay. The MTT assay is based on the reduction of yellow colored 3-(4,5-dimethylthiazol-2-yl)-2,5-diphenyltetrazolium bromide (MTT) (Acros Organics) by mitochondrial dehydrogenase activity in metabolically active cells to a blue-purple formazan that can be measured spectrophotometrically. The absorbances were read in an eight-channel computer-controlled photometer (Infinite M1000, Tecan), at two wavelengths (540 and 690 nm). All data were calculated using the median absorbance value of three wells. The 50% cytotoxic concentration (CC_{50}) was defined as the concentration of the test compound that reduced the absorbance (OD540) of the mock-infected control sample by 50%. The concentration achieving 50% protection against the cytopathic effect of the virus in infected cells was defined as the 50% effective concentration (EC_{50}).

III. Binding Analysis to HIV-1 CA Proteins *via* Surface Plasmon Resonance

3.1 Proteins

IgG b12 anti-HIV-1 gp120; was obtained through the NIH AIDS Reagent Program, Division of AIDS, NIAID, NIH: Anti-HIV-1 gp120 Monoclonal (IgG1 b12) from Dr. Dennis Burton and Carlos Barbas); p24 was produced in-house as previously described [3]. Briefly, a vector containing C-terminally His-tagged HIV-1_{NL4-3}CA (a gift from Dr. Eric Barklis, Oregon Health and Science University, Portland, OR) was transformed into BL21-Codon Plus (DE3)-RIL Competent Cells (Agilent Technologies, Wilmington, DE) and grown up in autoinduction ZYP-5052 medium overnight with shaking (225 rpm) at 30°C [4]. Bacterial cultures were spun down at 7000 rpm, and the supernatant was discarded. Cell pellets were resuspended in PBS and lysed via sonication. The resultant supernatant was clarified and immediately applied to a Talon cobalt resin affinity column (Clonetech Laboratories, Mountain View, CA). Protein was eluted using 1X PBS with 250 mM imidazole. Purified CA-H6 monomers were dialyzed overnight into 20 mM Tris-HCl pH 8.0 at 4°C, concentrated to 120 µM, flash-frozen in liquid nitrogen, aliquoted, and stored at -80°C. The CA hexamer was generated by introducing mutations at the following sites: A14C, E45C, W184A, and M185A through site-directed mutagenesis (Stratagene). The CA hexamer construct was expressed and purified following the same protocol as described above. After purification, the CA-H6 hexamers were dialyzed into 200mM ß-ME followed by sequential dialyzes to remove the ß-ME to allow for hexamer assembly slowly.

3.2 SPR direct interaction analysis

All binding assays were performed on a ProteOn XPR36 SPR Protein Interaction Array System (Bio-Rad Laboratories, Hercules, CA). The instrument temperature was set at 25 °C for all kinetic analyses. ProteOn GLH sensor chips were preconditioned with two short pulses each (10 s) of 50mM NaOH, 100mM HCl, and 0.5% sodium dodecyl

sulfide. Then the system was equilibrated with PBS-T buffer (20mM sodium phosphate, 150mM NaCl, and 0.005% polysorbate 20, pH 7.4). The surface of a GLH sensor chip was activated with a 1:100 dilution of a 1:1 mixture of 1-ethyl-3-(3-dimethylaminopropyl) carbodiimide hydrochloride (0.2 M) and sulfo-N-hydroxysuccinimide (0.05 M). Immediately after chip activation, the HIV-1 NL4-3 capsid protein constructs, purified as described above, were prepared at a concentration of 100 μg/mL in 10mM sodium acetate, pH 5.0 and injected across ligand flow channels for 5 min at a flow rate of 30 uL/min. Then, after unreacted protein had been washed out, excess active ester groups on the sensor surface were capped by a 5 min injection of 1M ethanolamine HCl (pH 8.0) at a flow rate of 5 μL/min. A reference surface was similarly created by immobilizing a non-specific protein (IgG b12 anti-HIV-1 gp120; was obtained through the NIH AIDS Reagent Program, Division of AIDS, NIAID, NIH: Anti-HIV-1 gp120 Monoclonal (IgG1 b12) from Dr. Dennis Burton and Carlos Barbas) and was used as a background to correct non-specific binding. To prepare a compound for direct binding analysis, compound stock solutions, along with 100% DMSO, and totalling 30 μL was made to a final volume of 1 mL by addition of sample preparation buffer (PBS, pH 7.4). Preparation of analyte in this manner ensured that the concentration of DMSO was matched with that of running buffer with 3% DMSO. Serial dilutions were then prepared in the running buffer (PBS, 3% DMSO, 0.005% polysorbate 20, pH 7.4) and injected at a flow rate of 100 μl/min, for a 1 min association phase, followed by up to a 5 min dissociation phase using the "one-shot kinetics" capability of the Proteon instrument. Data were analyzed using the ProteOn Manager Software version 3.0 (Bio-Rad). The responses from the reference flow cell were subtracted to account for the nonspecific binding and injection artifacts. Experimental data were fitted to a simple 1:1 binding model (where applied). The average kinetic (dissociation [k_d] rates) and equilibrium parameters generated from 3 replicates were used to define the off-rates and equilibrium dissociation constant (K_D).

IV. Molecular Dynamics Simulation

4.1 Initial structure preparation

The hexameric HIV-1 CA (5HGL) was used in the molecular dynamics simulation study (MD) [5]. One monomer of the hexameric structure was used in the molecular dynamics simulation. The x-ray structure misses amino acids Ala88 to Gln95 and Lys182 to Ala185. Accordingly, we used x-ray structure 3GV2 to obtain the missed amino acids with their corresponding tertiary structure. Then, these amino acids were added to 5HGL structure after their alignment using discovery studio software [6]. The modified structure was energy minimized to remove any strains.

Compound **F_2-7f** was sketched in its S-configuration by Marvin, Marvin 20.21, ChemAxon (https://www.chemaxon.com). Also, Marvin was used to check the ionization states of **F_2-7f**, where no ionisable groups were detected at physiological pH. Then, the conformers of **F_2-7f** were generated by OMEGA, OPENEYE Scientific Software Inc. [7,8]. The generated conformers were used in the subsequent docking procedure to the binding site of HIV-1 CA monomer by OPENEYE Scientific Software Inc. module OEDOCKING 3.0.1 [9-12]. The conformer with the highest score was used in the subsequent molecular dynamics simulation.

4.2 Molecular dynamics simulation production

Atomic charges of **F_2-7f** atoms were derived from AM1-BCC charge model with ANTECHAMBER module of AMBER14 [13]. Force field ff14sb was used to generate parameters of the protein residues, and GAFF force field for **F_2-7f**. The complex was solvated by TIP3PBOX octahedral water model with 8Å cut, and the system was neutralized by Na^+ ions. The system was minimized in two steps: in the first step the solvent was minimized for 10000 cycles using steepest descent and then conjugate gradient algorithms, and in the second step the whole system was minimized for 5000 cycles using steepest descent followed by conjugate gradient algorithms. Water was then heated to 300 K for 20 ps at constant volume and periodic boundaries with weak strength restraints. Then, the whole system was equilibrated for 40 ps using constant pressure periodic boundaries. The equilibrated structure was used in the subsequent 500 ns NPT MD simulation. Non-bonded forces were calculated at a Cutoff distance of 10 Å.

4.3 Clustering

The trajectory was imaged and water molecules and Na^+ ions were stripped off. All frames were aligned against the first frame of the MD simulation using protein only. Then, the MD trajectory was clustered by DBSCAN algorithm [7,14] on **F_2-7f** using minimum points of 2.0 and epsilon of 2.1 with no frame orientation (no fit). This clustered all frames according to the **F_2-7f**.

V. *In silico* ADMET Analysis

Drug-like properties, metabolic stability, and toxicity were assessed using the P450 and Derek Nexus module within Stardrop V7 [15] (Optibrium, Ltd., Cambridge, UK).

For metabolic stability evaluation, representative compounds were docked into the human CYP3A4 isoform (PDB ID 4D78). CYP3A4 was prepared using Autodock tools, where essential hydrogen atoms, Kollman united atom type charges, and solvation parameters were added. The grid box for the docking search was centered around the catalytic center of CYP3A4 [16-18]. Docking calculations were performed using AutoDock via DockingServer [19]. The docked poses were further evaluated with SeeSAR 12.1 (BioSolveIT Gmbh, Germany) utilizing the predictive binding affinity calculations using the HYdrogen bond and DEhydration (HYDE) energy scoring function [20-22]. The lower boundary of the affinity prediction for the poses with acceptable torsion angles and clash scores were used in combination with composite site lability (CSL), and number of labile sites within the molecule to predict metabolic stability.

VI. Metabolic Stability in Human Liver Microsomes

The metabolic stability in human liver microsomes of compounds was determined in WuXi AppTec Co. Ltd. (Shanghai), China. The detailed procedure is as follows:

1. Test Compounds

Table S1. Compounds information

Compound No.	Compound ID	Batch No.	Exact Mass	Stock Concentration (mM)
1	F_2-7f	F_2-7f	635.24	10
2	PF74	PF74	425.21	10

Control	Testosterone	288.42	10
Control	Diclofenac	295.14	10
Control	Propafenone	341.44	10

2. Experimental Procedure

2.1. Test Compound and Control Working Solution Preparation

2.1.1. Working solution: 5 μL of compound and control stock solution (10 mM in dimethyl sulfoxide (DMSO)) were diluted with 495 μL of acetonitrile (ACN) (intermediate solution concentration: 100 μM, 99% ACN).

2.2. NADPH Cofactor Preparation

2.2.1. Materials

NADPH powder: β-Nicotinamide adenine dinucleotide phosphate reduced form, tetrasodium salt; NADPH·4Na (Vendor: Chem-Impex International, Cat. No. 00616).

2.2.2. Preparation Procedure

The appropriate amount of NADPH powder was weighed and diluted into a 10 mM $MgCl_2$ solution (working solution concentration: 10 mM; final concentration in reaction system: 1 mM).

2.3. Liver Microsomes Preparation

2.3.1. Materials

Table S2. Liver Microsomes Information

Species	Product Information	Vendor	Abbrevation
Human	Cat No. 452117<br>Lot No. 38295	Corning	HLM

2.3.2. Preparation Procedure

The appropriate concentrations of microsome working solutions were prepared in 100 mM potassium phosphate buffer.

2.4. Stop Solution Preparation

Cold (4°C) acetonitrile (ACN) containing 200 ng/mL tolbutamide and 200 ng/mL labetalol as internal standards (IS) was used as the stop solution.

2.5. Assay Procedure

2.5.1. Pre-warm empty 'Incubation' plates T60 and NCF60 for 10 min minutes.

2.5.2. Dilute liver microsomes to 0.56 mg/mL in 100 mM phosphate buffer.

2.5.3. Transfer 445 uL microsome working solutions (0.56 mg/mL) into pre-warmed 'Incubation' plates T60 and NCF60, Then pre-incubate 'Incubation' plates T60 and NCF60 for 10 min at 37°C with constant shaking. Transfer 54 μL liver microsomes to blank plate, then add 6 μL NAPDH cofactor to blank plate, and then add 180 μL quenching solution to blank plate.

2.5.4 Add 5 μL compound working solution (100 μM) into 'incubation' plates (T60 and NCF60) containing microsomes and mix 3 times thoroughly.

2.5.5. For the NCF60 plate, add 50 uL of buffer and mix 3 times thoroughly. Start timing; plate will be incubated at 37°C for 60 min while shaking.

2.5.6. In 'Quenching' plate T0, add 180 μL quenching solution and 6 μL NAPDH cofactor. Ensure the plate is chilled to prevent evaporation.

2.5.7. For the T60 plate, mix 3 times thoroughly, and immediately remove 54 μL mixture for the 0-min time point to 'Quenching' plate. Then add 44 μL NAPDH cofactor to incubation plate (T60). Start timing; plate will be incubated at 37°C for 60 min while shaking.

Table S3. Final Concentration of Each Component in Incubation Medium

Component	Concentration
Microsome	0.5 mg protein/mL
Test Compound	1 μM
Control Compound	1 μM
Acetonitrile	0.99%
DMSO	0.01%

2.5.8. At 5, 15, 30, 45, and 60 min, add 180 μL quenching solution to 'Quenching' plates, mix once, and serially transfer 60 μL sample from T60 plate per time point to 'Quenching' plates.

Table S4. Reaction Plates Incubation

Time Point	Start Time	End Time
Blank	1:00:00	0:00:00
T60	1:00:00	0:00:00
T45	0:45:00	0:00:00

T30	0:30:00	0:00:00
T15	0:15:00	0:00:00
T5	0:05:00	0:00:00
T0	mix 3 times and remove out to 'Quenching' plate	

2.5.9. For NCF60: mix once, and transfer 60 μL sample from the NCF60 incubation to 'Quenching' plate containing quenching solution at the 60-min time point.

Table S5. NCF60 Incubation

Time Point	Start Time	End Time
NCF60	1:00:00	0:00:00

2.5.10. All sampling plates are shaken for 10 min, then centrifuged at 4000 rpm for 20 minutes at 4°C.

2.5.11. Transfer 80 μL supernatant into 240 μL HPLC water, and mix by plate shaker for 10 min.

2.5.12. Each bioanalysis plate was sealed and shaken for 10 minutes prior to LC-MS/MS analysis.

3. Data Analysis

3.1. The equation of first order kinetics was used to calculate T1/2 and CLint(mic) (μL/min/mg).

Equation of first order kinetics:

$$C_t = C_0 \bullet e^{-k_e \bullet t}$$

$$\text{when } C_t = \frac{1}{2} C_0 \text{ ,}$$

$$T_{1/2} = \frac{Ln2}{k_e} = \frac{0.693}{k_e}$$

$$CL_{int(mic)} = \frac{0.693}{\text{In vitro } T_{1/2}} \bullet \frac{1}{\text{mg / mL microsomal protein in reaction system}}$$

$$CL_{int(liver)} = CL_{int(mic)} \bullet \frac{\text{mg microsomes}}{\text{g liver}} \bullet \frac{\text{g liver}}{\text{kg body weight}}$$

4. Raw Data

Compound ID	Compound & Species	Time (min)	Analyte Peak Area	IS Peak Area	Analyte/IS	% Remaining	Time (min)	% Remaining	Ln (%Remaining)		R^2	k_e (min^{-1})	$T_{1/2}$ (min)	$CL_{int(mic)}$ (μL/min/mg)	Remaining (T=60min)	Remaining (NCF=60min)
F_2-7f	F_2-7f HLM 0.5	Blank	0	107,580	0.000	0.0					1.0000	1.3796	0.5	2759.1	0.0%	103.5%
F_2-7f	F_2-7f HLM 0.5	60	77	105,915	0.001	0.0				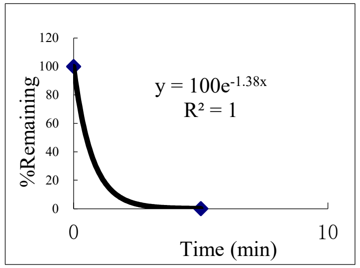						
F_2-7f	F_2-7f HLM 0.5	45	90	109,932	0.001	0.0										
F_2-7f	F_2-7f HLM 0.5	30	153	108,625	0.001	0.0										
F_2-7f	F_2-7f HLM 0.5	15	149	103,094	0.001	0.1										
F_2-7f	F_2-7f HLM 0.5	5	297	103,381	0.003	0.1	5	0.1	-2.3							
F_2-7f	F_2-7f HLM 0.5	0	282,308	98,897	2.855	100.0	0	100.0	4.6							
F_2-7f	F_2-7f HLM 0.5	NCF60	319,373	108,087	2.955	103.5										
PF74	PF74HLM 0.5	Blank	0	116,908	0.000	0.0					1.0000	1.4312	0.5	2862.5	0.0%	112.6%
PF74	PF74HLM 0.5	60	0	115,817	0.000	0.0				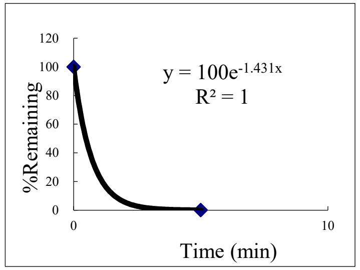						
PF74	PF74HLM 0.5	45	0	102,033	0.000	0.0										
PF74	PF74HLM 0.5	30	61	106,730	0.001	0.0										
PF74	PF74HLM 0.5	15	212	99,964	0.002	0.0										
PF74	PF74HLM 0.5	5	1,231	104,970	0.012	0.1	5	0.1	-2.6							
PF74	PF74HLM 0.5	0	1,486,739	98,654	15.070	100.0	0	100.0	4.6							
PF74	PF74HLM 0.5	NCF60	1,738,223	102,451	16.966	112.6										
Diclofenac	DiclofenacHLM 0.5	Blank	0	105,690	0.000	0.0					0.9947	0.1860	3.7	372.0	0.0%	96.7%

Diclofenac	DiclofenacHLM 0.5	60	0	104,657	0.000	0.0									
Diclofenac	DiclofenacHLM 0.5	45	0	98,189	0.000	0.0									
Diclofenac	DiclofenacHLM 0.5	30	303	101,980	0.003	0.4	30	0.4	-1.0						
Diclofenac	DiclofenacHLM 0.5	15	3,460	99,988	0.035	4.2	15	4.2	1.4						
Diclofenac	DiclofenacHLM 0.5	5	26,146	102,565	0.255	30.7	5	30.7	3.4						
Diclofenac	DiclofenacHLM 0.5	0	82,212	98,999	0.830	100.0	0	100.0	4.6						
Diclofenac	DiclofenacHLM 0.5	NCF60	83,751	104,298	0.803	96.7									

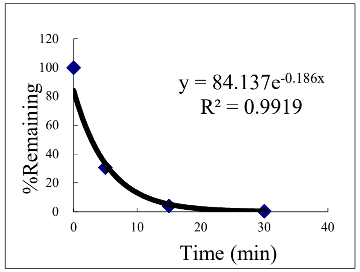

Propafenone	PropafenoneHLM 0.5	Blank	63	105,722	0.001	0.0				0.9350	0.1397	5.0	279.5	0.0%	93.6%
Propafenone	PropafenoneHLM 0.5	60	63	100,264	0.001	0.0									
Propafenone	PropafenoneHLM 0.5	45	1,024	97,056	0.011	0.2	45	0.2	-1.9						
Propafenone	PropafenoneHLM 0.5	30	40,148	101,776	0.394	5.6	30	5.6	1.7						
Propafenone	PropafenoneHLM 0.5	15	258,004	95,967	2.688	38.5	15	38.5	3.7						
Propafenone	PropafenoneHLM 0.5	5	499,263	104,023	4.800	68.7	5	68.7	4.2						
Propafenone	PropafenoneHLM 0.5	0	659,116	94,347	6.986	100.0	0	100.0	4.6						
Propafenone	PropafenoneHLM 0.5	NCF60	671,724	102,762	6.537	93.6									

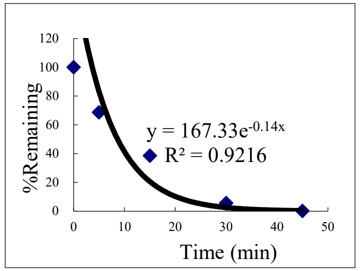

Testosterone	TestosteroneHLM 0.5	Blank	223	112,647	0.002	0.2				0.9982	0.0414	16.7	82.8	7.9%	90.7%
Testosterone	TestosteroneHLM 0.5	60	7,607	108,076	0.070	7.9	60	7.9	2.1						
Testosterone	TestosteroneHLM 0.5	45	14,283	107,692	0.133	14.9	45	14.9	2.7						
Testosterone	TestosteroneHLM 0.5	30	25,374	103,996	0.244	27.5	30	27.5	3.3						
Testosterone	TestosteroneHLM 0.5	15	47,238	100,489	0.470	53.0	15	53.0	4.0						

Testosterone	TestosteroneHLM 0.5	5	66,880	104,311	0.641	72.2	5	72.2	4.3	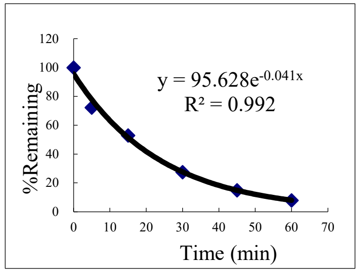
Testosterone	TestosteroneHLM 0.5	0	90,117	101,541	0.887	100.0	0	100.0	4.6	
Testosterone	TestosteroneHLM 0.5	NCF60	80,291	99,693	0.805	90.7				

VII. Metabolic Stability in Human Plasma

The Stability in Human Plasma of compounds was determined in WuXi AppTec Co. Ltd. (Shanghai), China. The detailed procedure is as follows:

1. Materials

1.1. Test Compounds and Stock Solutions

Compound ID	Batch	MW	FW	Purity (%)	Stock Conc. (mM)	Final Conc.(μM)
F_2-7f	F_2-7f	635.6	NA	95.9	10	2
PF74	PF74	425.5	NA	97.1	10	2
Propantheline bromide	R000190915	448.39	448.39	97.00	10	2

1.2. Test Compound and Control Working Solution Preparation

1.2.1. Test compound Working solution: 5 μL of compound stock solution (10 mM in dimethyl sulfoxide (DMSO)) were diluted with 495 μL of DMSO (Working solution concentration: 100 uM, 100% DMSO).

1.2.2. Propantheline bromide Working solution: 5 μL of Propantheline bromide stock solution (10 mM in H_2O) were diluted with 495 μL of H_2O (Working solution concentration: 100 uM, 100% H_2O).

1.3. Test System

Species / Matrix	Minimum No. of Individuals	Anticoagulant Used	Vendor	Cat#	Batch
Human Plasma	3 Male & 3 Female	EDTA-K2	Bioreclamation IVT	HUMANPLK2 P2N	HMN514548

2. Methods

2.1. The pooled frozen plasma was thawed in a water bath at 37°C prior to experiment. Plasma was centrifuged at 4000 rpm for 5 min and the clots were removed if any.

2.2. Using an Apricot automation workstation, 98 μL/well of blank plsma were added to all 96-well reaction plates. (Blank, T0, T10, T30, T60, and T120)

2.3. An Apricot automation workstation was used to add 2 μL/well of working solution (100 μM) to all reaction plates except Blank. (T0, T10, T30, T60, and T120)

2.4. All reaction plates containing mixtures of compound and plasma were incubated at

37°C in water bath.

2.5. The reaction plates were incubated at 37°C, and timer was started.

Table S6. Reaction Plates Incubation

Time Point	Start Time	End Time
Blank	0:00:00	0:00:00
T120	2:00:00	0:00:00
T60	1:00:00	0:00:00
T30	0:30:00	0:00:00
T10	0:10:00	0:00:00
T0		

2.6. At the end of incubation, added 400 μL of stop solution (200 ng/mL tolbutamide and 200 ng/mL labetalol in ACN) to precipitate protein. Mixed thoroughly.

2.7. Each plate was sealed and shaken for 20 minutes

2.8. After shaking, each plate was centrifuged at 4000 rpm and 4°C for 20 minutes

2.9. After centrifugation, an Apricot automation workstation was used to transfer 150 μL supernatant.

2.10. Each bioanalysis plate was sealed and shaken for 10 minutes prior to LC-MS/MS analysis

3. Data Analysis

The % remaining of test compound after incubation in plasma was calculated using following equation:

% Remaining= 100 x (PAR at appointed incubation time / PAR at T0 time)

where PAR is the peak area ratio of analyte versus internal standard (IS)

The appointed incubation time points are T0 (0 min), Tn (n=0, 10, 30, 60, 120 min)

4. Raw data

Sample_ID	Time (min)	Analyte Peak Area	IS Peak Area	Aa/Ai	% Remaining (n=2)	Ln (% Remaining)			
F2-7f _H_0	0	3.49E+06	1.36E+06	2.5621	100.0	0.00	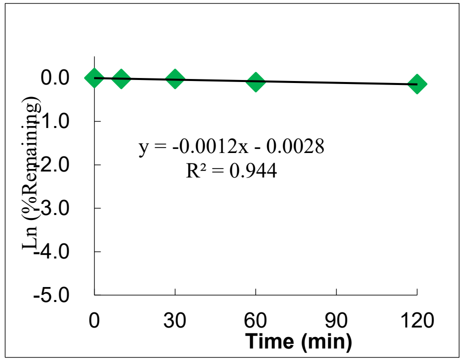	k=	-0.00121
F2-7f _H_0		3.27E+06	1.35E+06	2.4205				R^2=	0.9440
F2-7f _H_10	10	3.35E+06	1.45E+06	2.3071	98.0	-0.02		t1/2=0.693/k	>289.1
F2-7f _H_10		3.70E+06	1.44E+06	2.5743					
F2-7f _H_30	30	3.80E+06	1.62E+06	2.3457	97.8	-0.02			
F2-7f _H_30		4.37E+06	1.73E+06	2.5284					
F2-7f _H_60	60	3.42E+06	1.54E+06	2.2253	90.9	-0.10			
F2-7f _H_60		3.27E+06	1.42E+06	2.3020					
F2-7f _H_120	120	3.18E+06	1.50E+06	2.1242	99.3	-0.01			
F2-7f _H_120		3.37E+06	1.53E+06	2.2043					
PF74_H_0	0	1.03E+07	1.45E+06	7.1259	86.9	-0.14	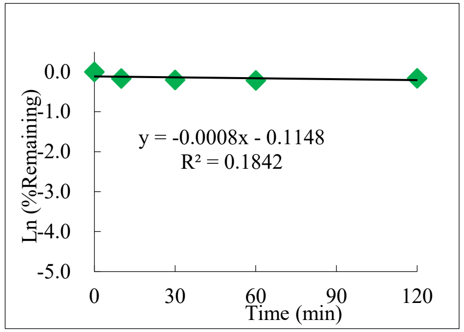	k=	0.0008
PF74_H_0		1.08E+07	1.56E+06	6.8745				R^2=	0.1842
PF74_H_10	10	9.50E+06	1.59E+06	5.9628	84.7	-0.17		t1/2=0.693/k	>289.1
PF74_H_10		8.68E+06	1.47E+06	5.8962					
PF74_H_30	30	8.42E+06	1.54E+06	5.4832	81.7	-0.20			
PF74_H_30		8.91E+06	1.50E+06	5.9541					
PF74_H_60	60	8.74E+06	1.42E+06	6.1381	80.7	-0.21			
PF74_H_60		7.65E+06	1.48E+06	5.1652					
PF74_H_120	120	8.79E+06	1.61E+06	5.4640	85.2	-0.16			
PF74_H_120		1.04E+07	1.62E+06	6.4621					
Propantheline bromide_H_0	0	3.32E+06	1.74E+06	1.9093	100.0	0.00		k=	0.0617

Propantheline bromide_H_0		3.22E+06	1.64E+06	1.9647			R^2=	0.9961
Propantheline bromide_H_10	10	2.01E+06	1.86E+06	1.0818	56.5	-0.57	t1/2=0.693/k	11.2
Propantheline bromide_H_10		1.91E+06	1.73E+06	1.1067				
Propantheline bromide_H_30	30	6.75E+05	1.89E+06	0.3581	19.4	-1.64		
Propantheline bromide_H_30		7.44E+05	1.90E+06	0.3922				
Propantheline bromide_H_60	60	6.60E+04	1.77E+06	0.0374	1.9	-3.99		
Propantheline bromide_H_60		6.11E+04	1.78E+06	0.0344				
Propantheline bromide_H_120	120	3.75E+03	1.98E+06	0.0019	0.1	-7.29		
Propantheline bromide_H_120		1.14E+03	1.52E+06	0.0007				

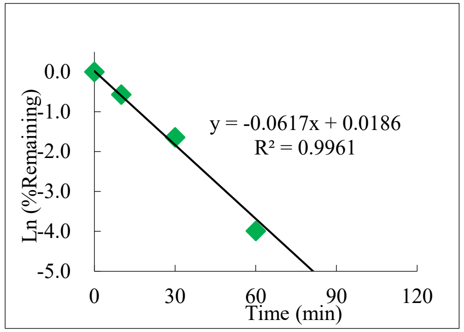

VIII. ^{1}H-NMR and ^{13}C-NMR and HRMS Spectra for Representative Target Compounds

1. ^{1}H-NMR, ^{13}C-NMR and HRMS Spectra for 7a

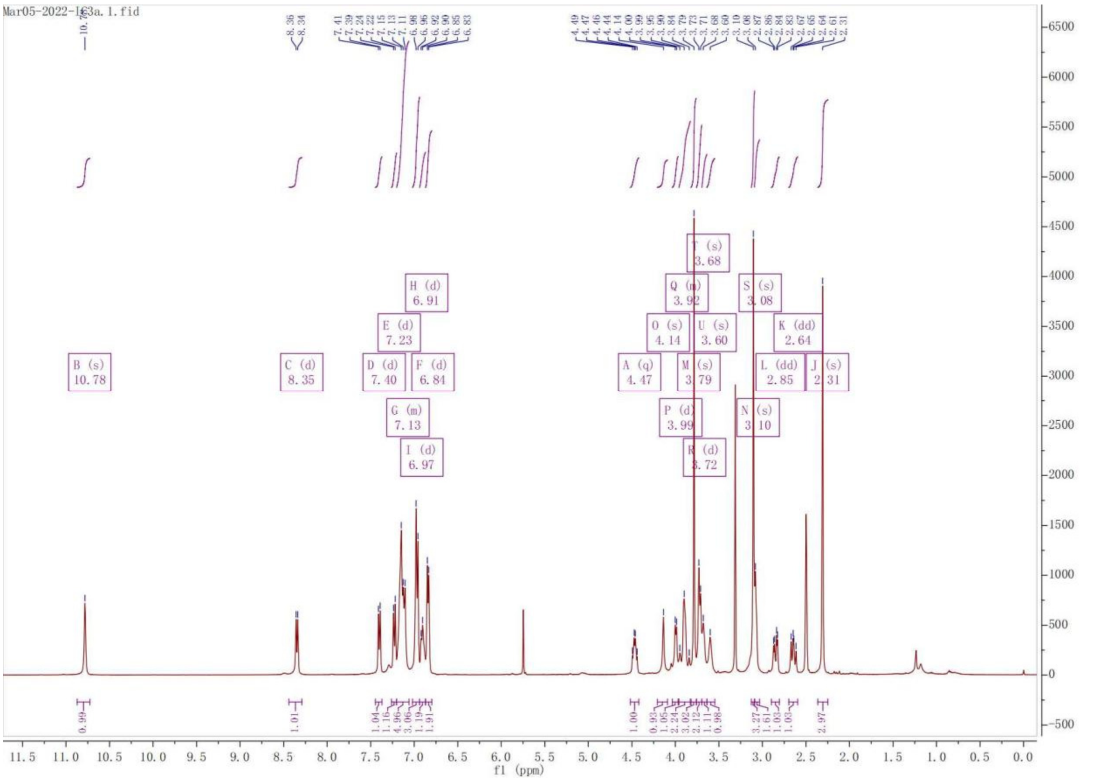

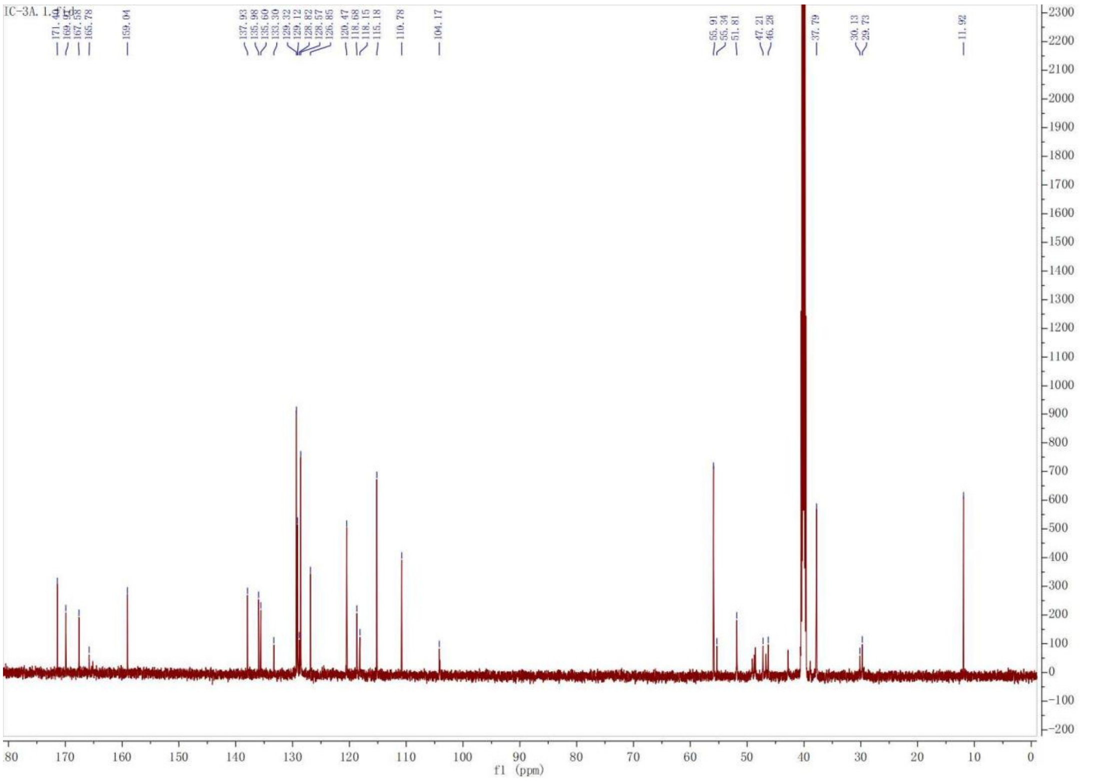

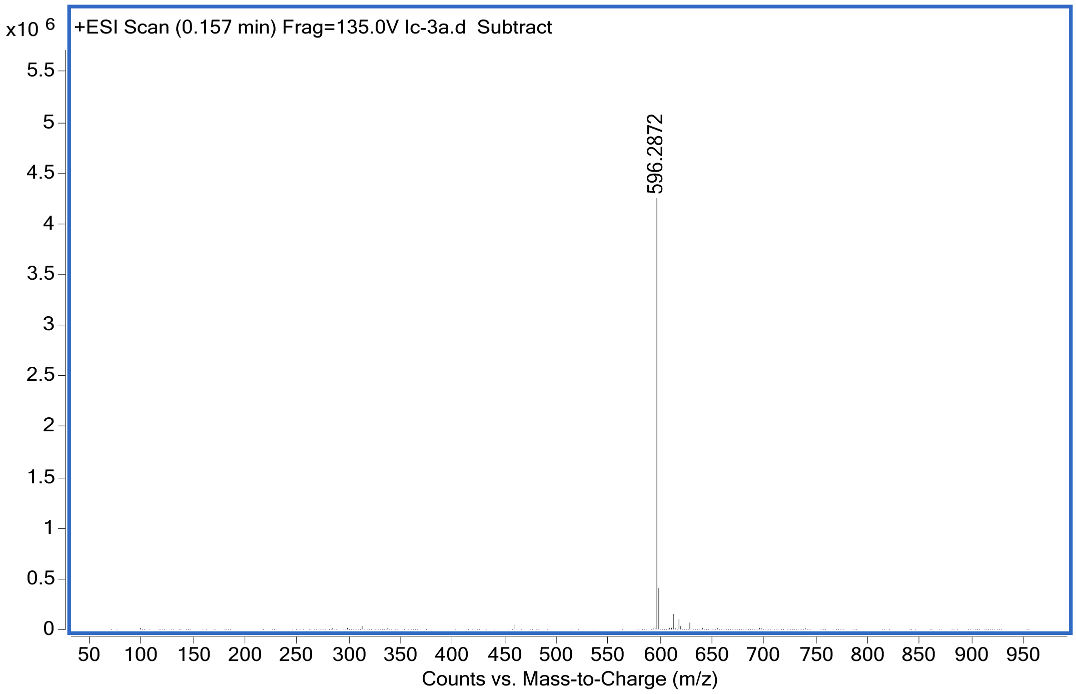

2. ^{1}H-NMR, ^{13}C-NMR and HRMS Spectra for 7b

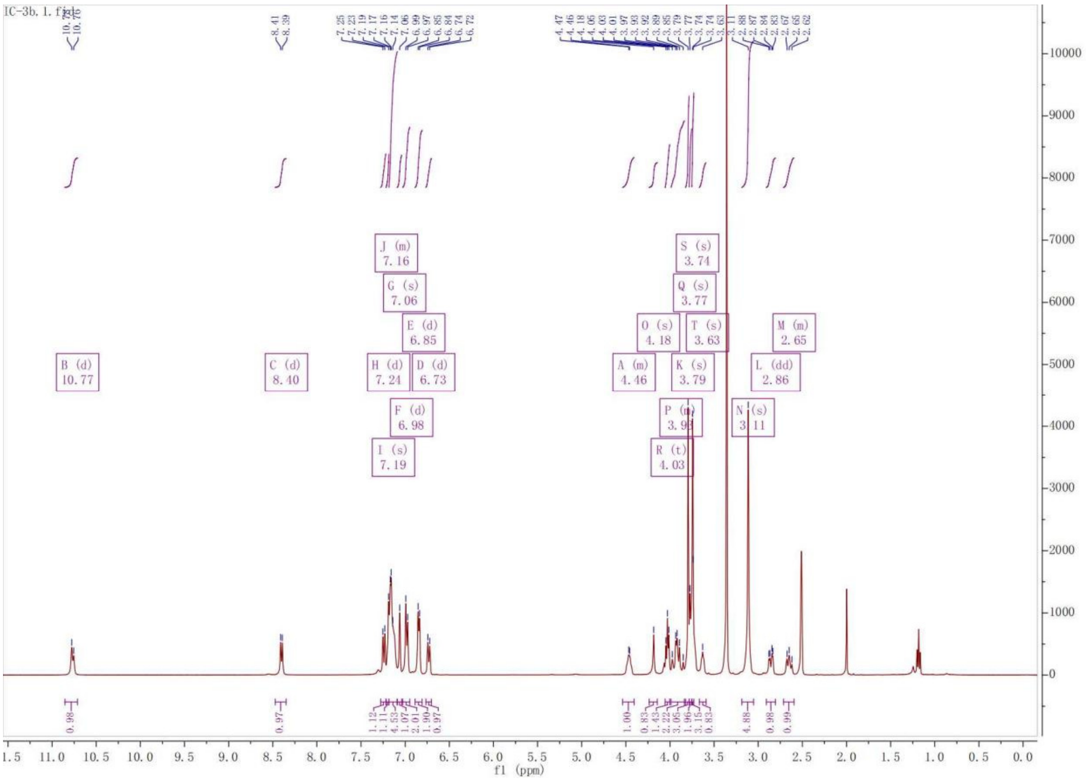

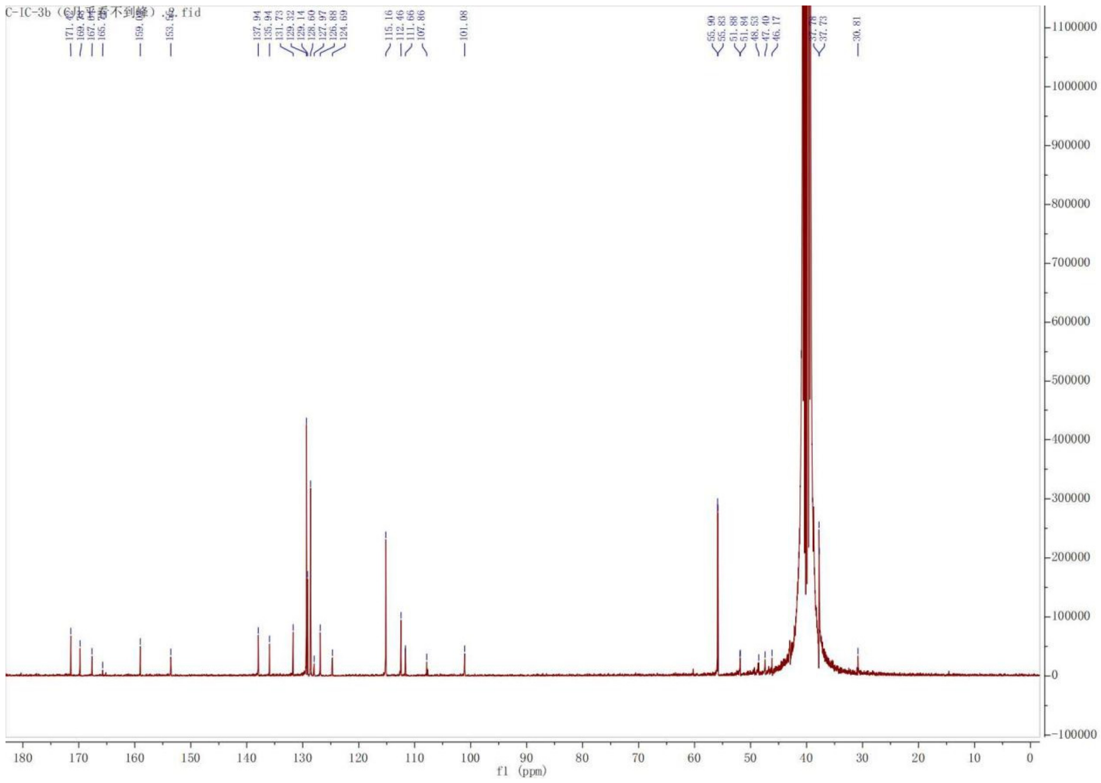

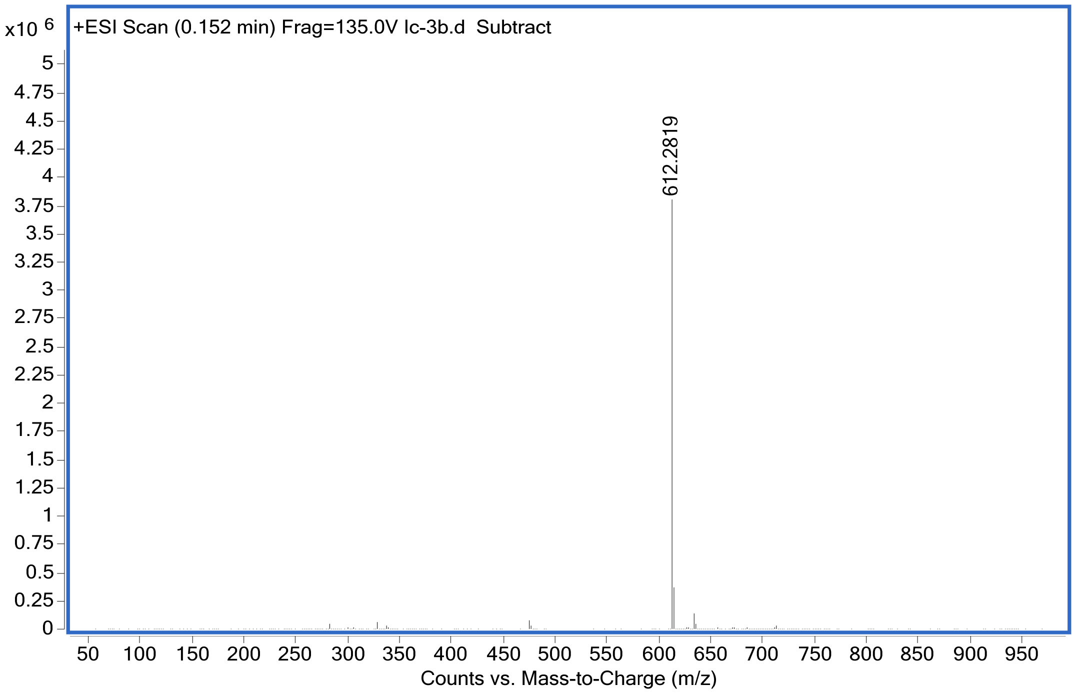

3.

4. ^{1}H-NMR, ^{13}C-NMR and HRMS Spectra for 7c

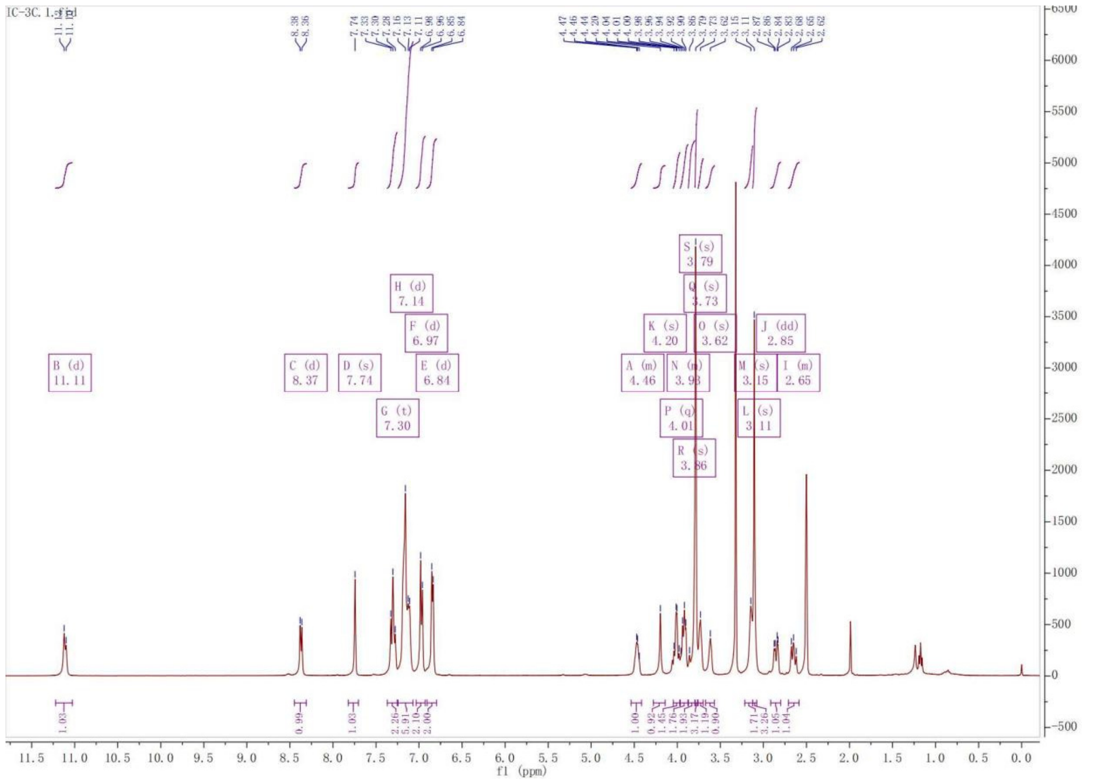

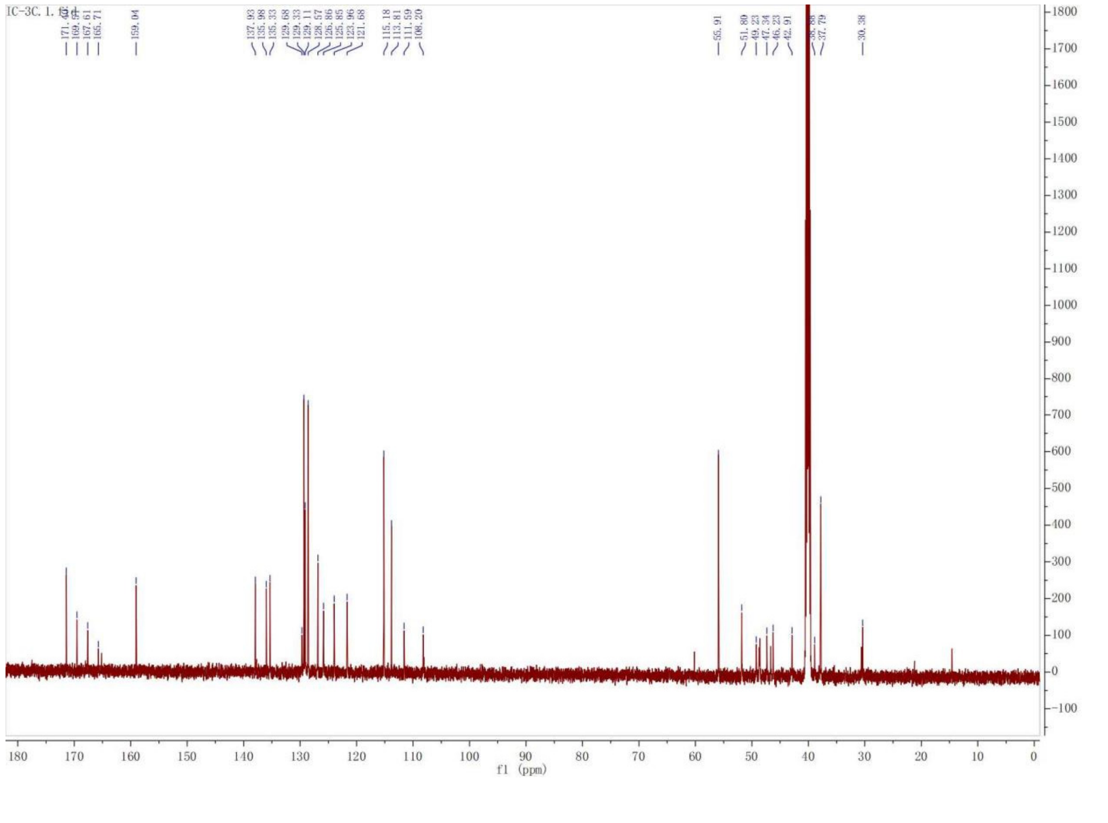

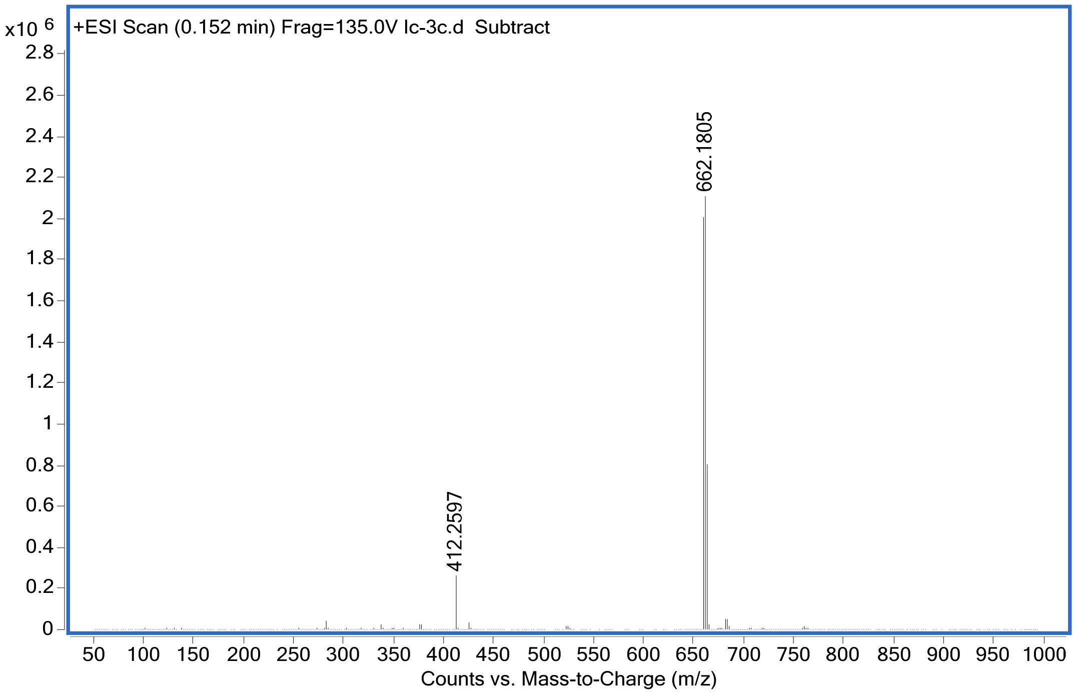

5.

6. ^{1}H-NMR, ^{13}C-NMR and HRMS Spectra for 7d

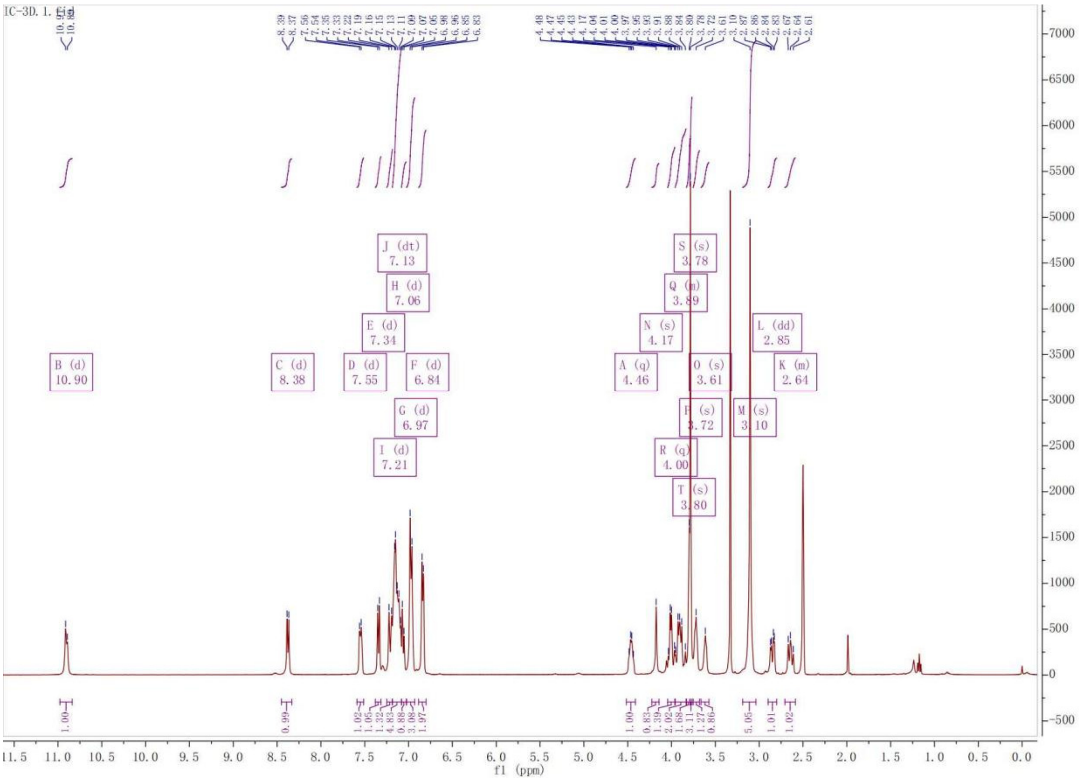

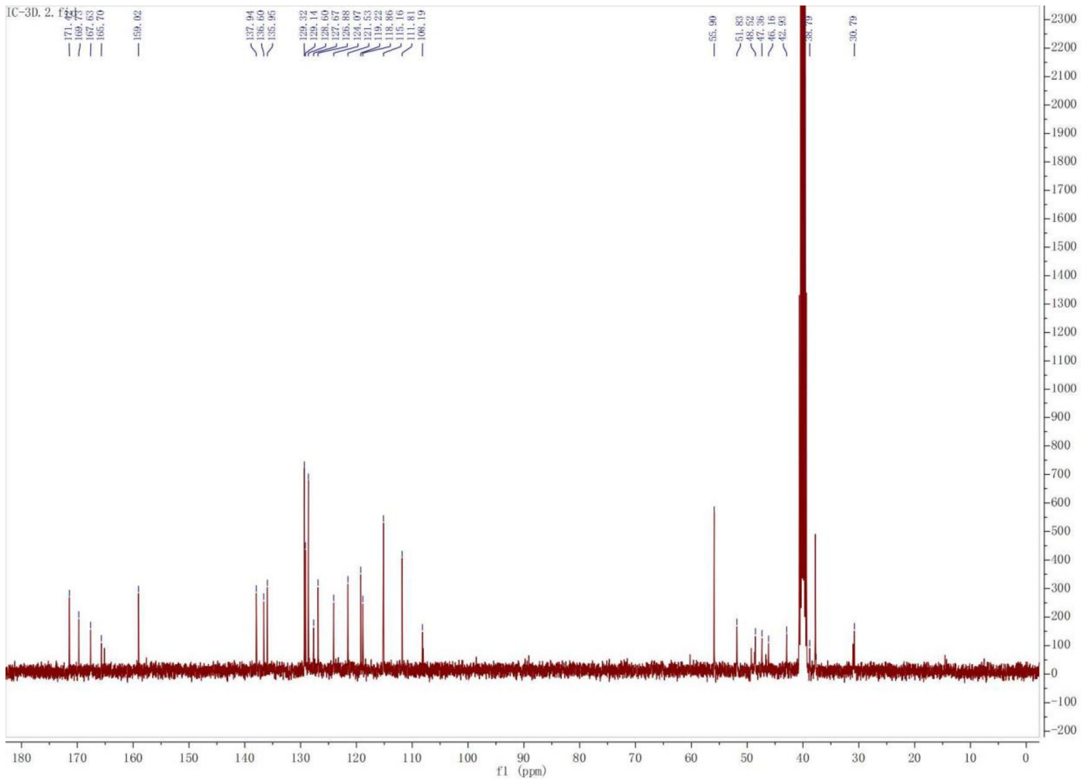

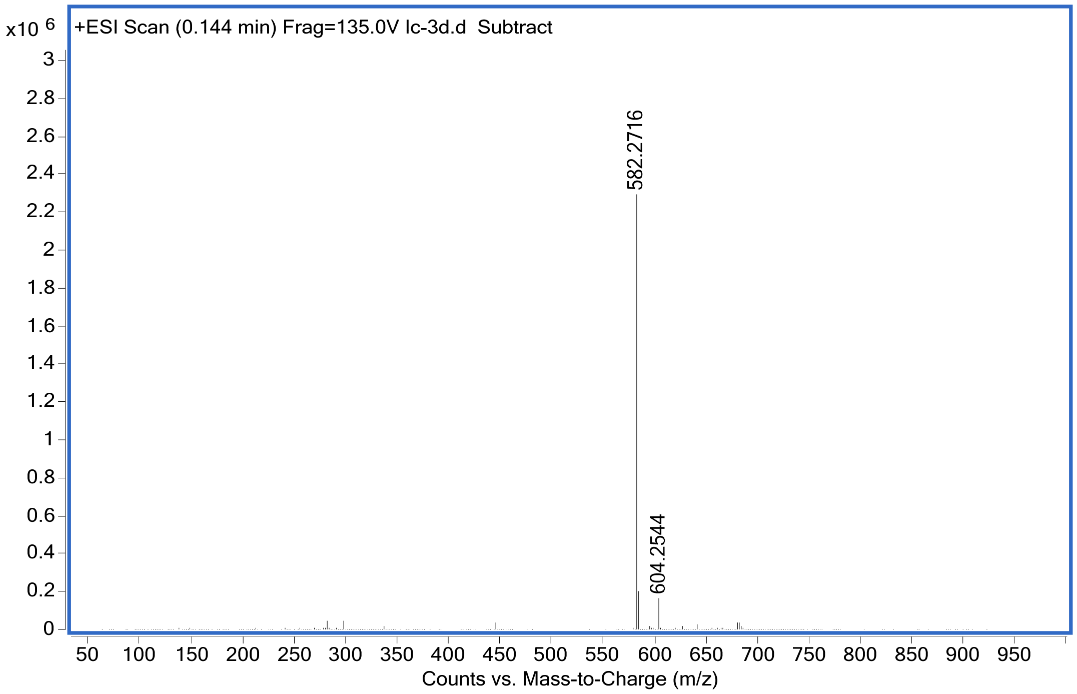

7.

8. ^{1}H-NMR, ^{13}C-NMR and HRMS Spectra for 7e

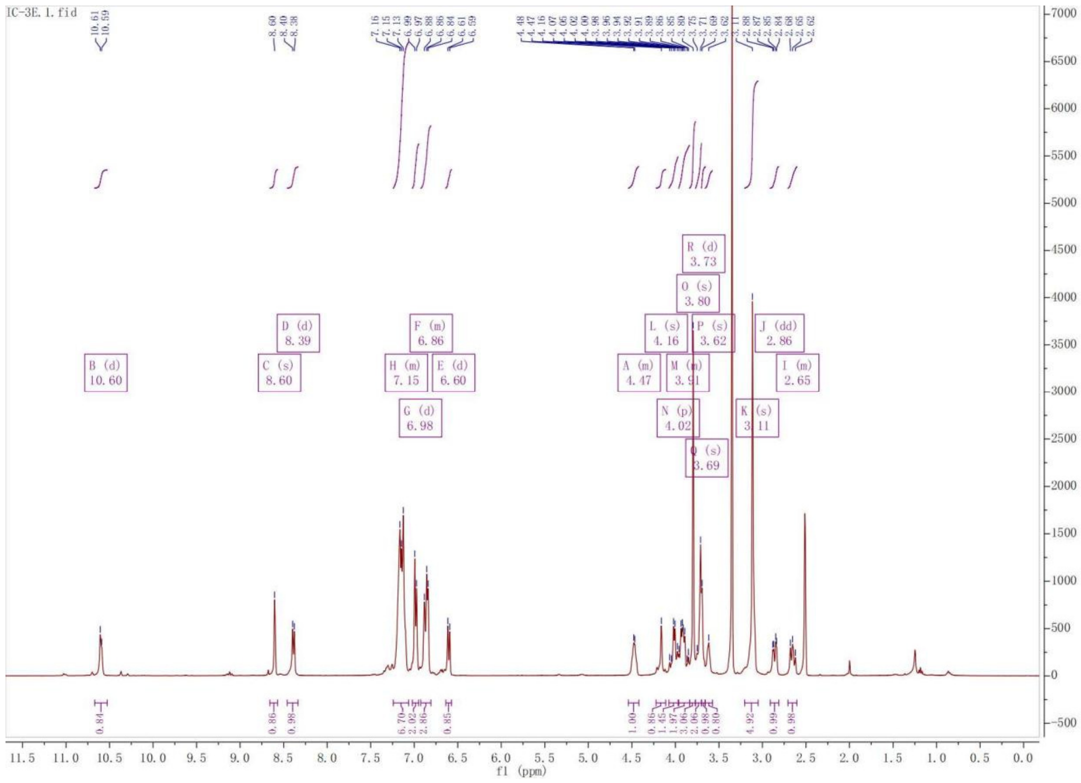
IC-3E.1.fid
B (d) 10.60
C (s) 8.60
D (d) 8.39
H (m) 7.15
F (m) 6.86
E (d) 6.60
G (d) 6.98
A (m) 4.47
L (s) 4.16
M (m) 3.91
N (p) 4.02
R (d) 3.73
O (s) 3.80
P (s) 3.62
Q (s) 3.69
K (s) 3.11
J (dd) 2.86
I (m) 2.65
f1 (ppm)

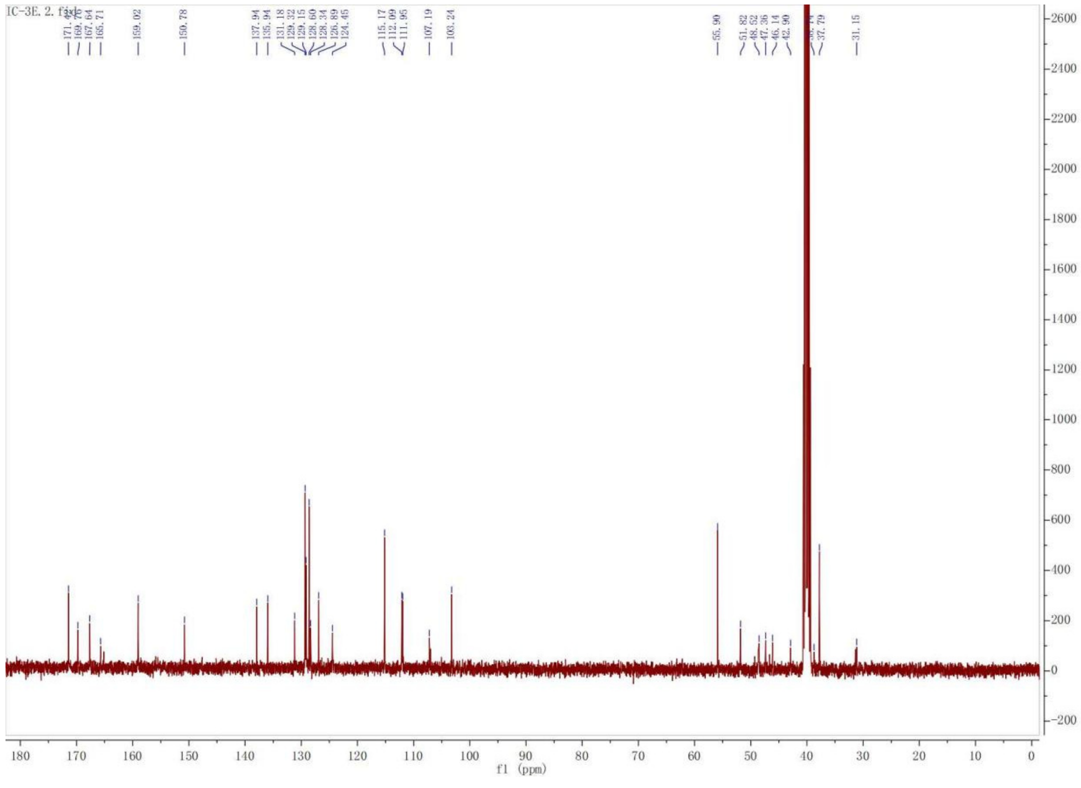
IC-3E.2.fid
f1 (ppm)

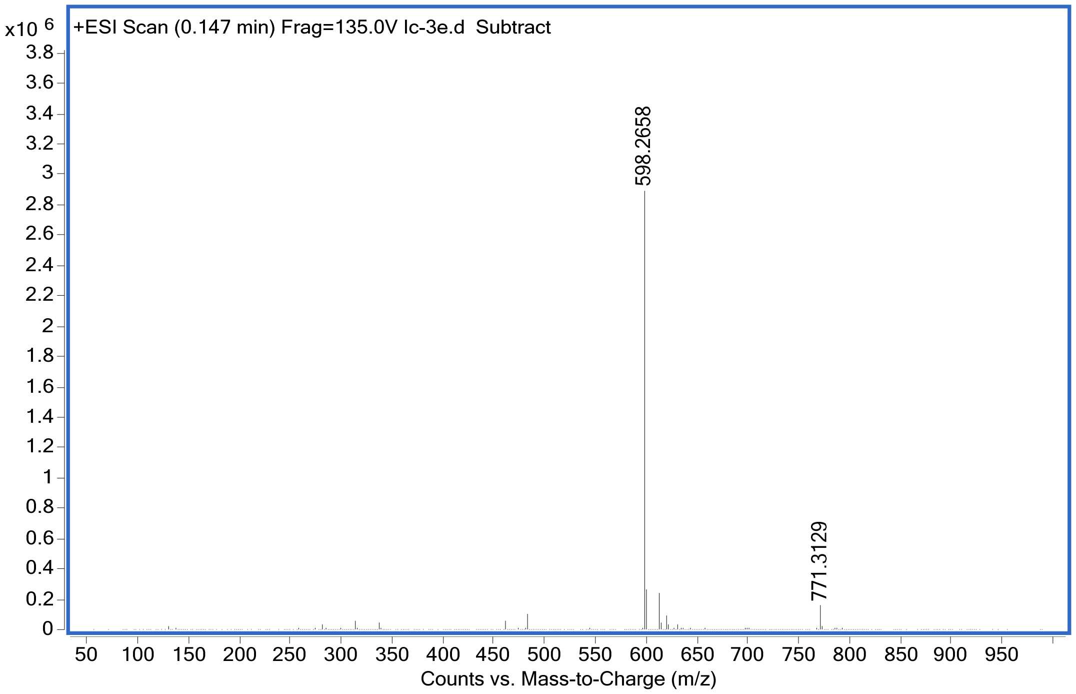

9.

10. ^{1}H-NMR, ^{13}C-NMR and HRMS Spectra for 7f

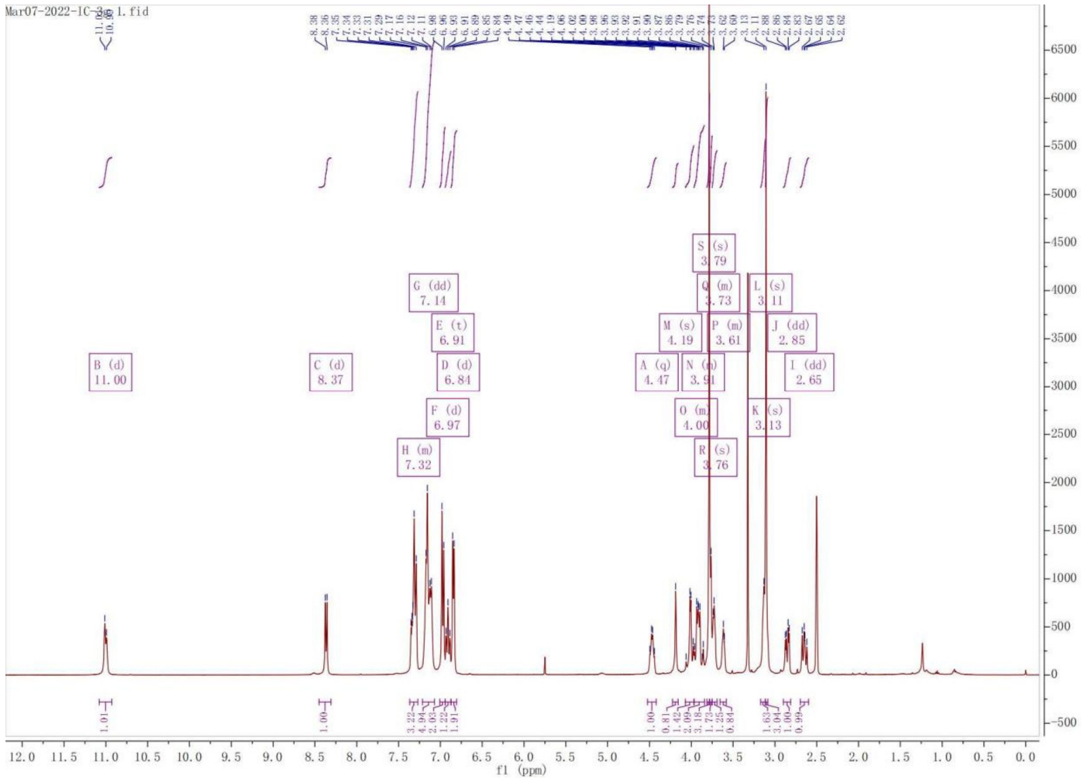

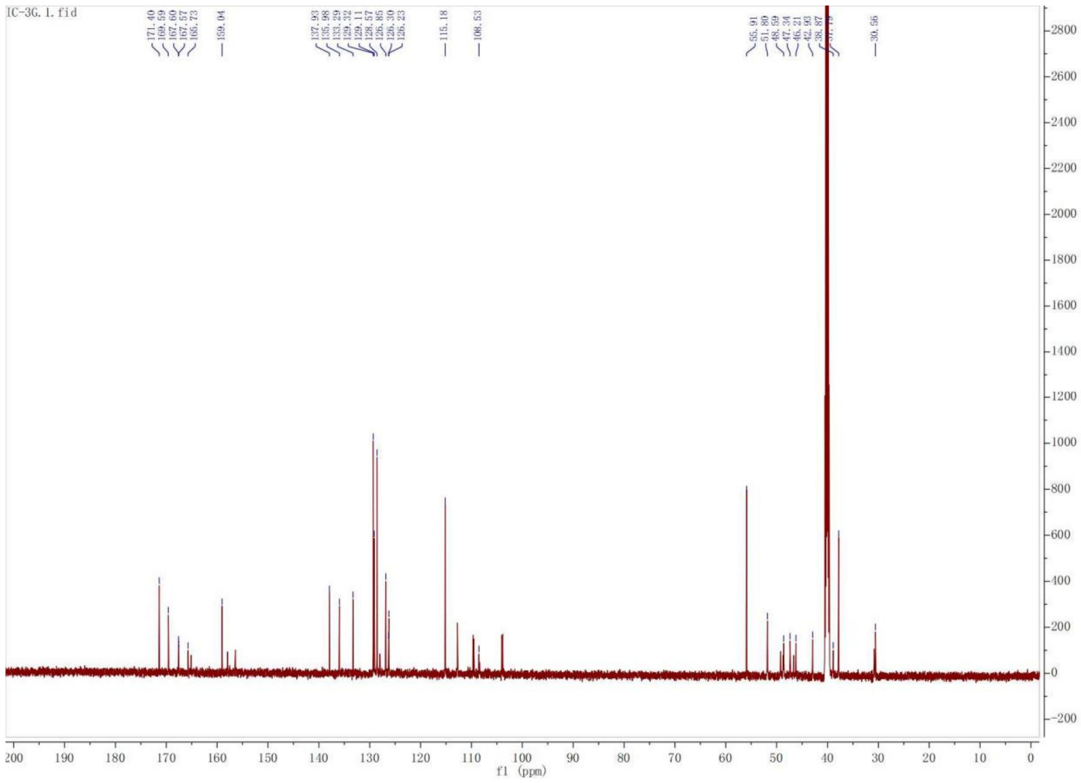

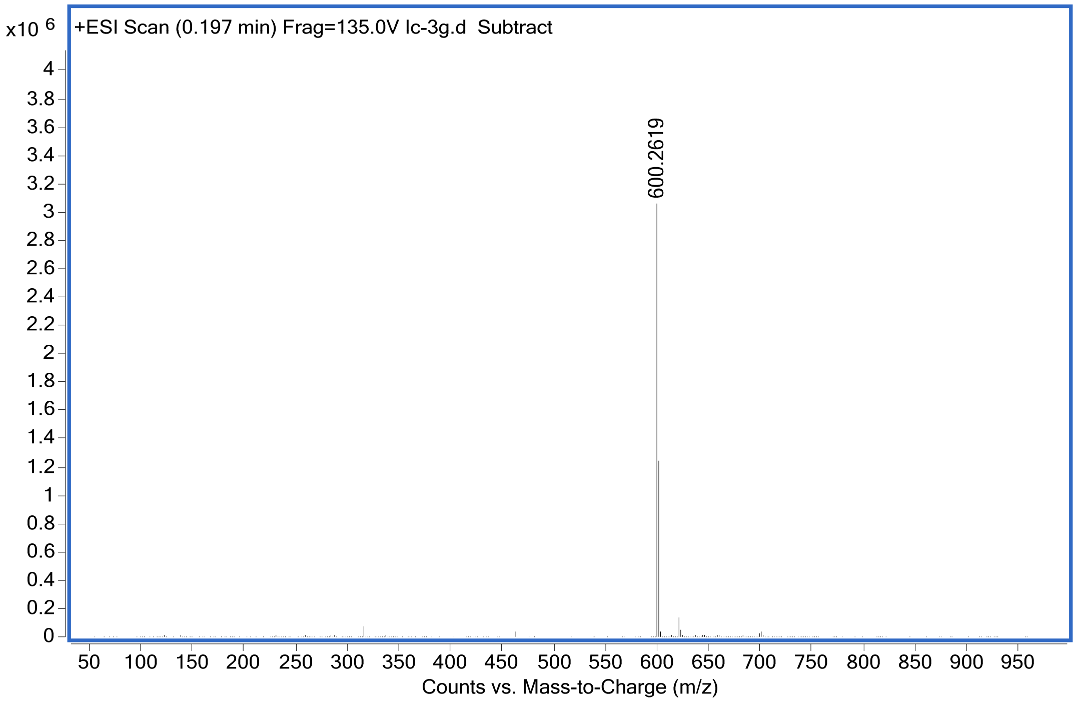

11. ^{1}H-NMR, ^{13}C-NMR and HRMS Spectra for F_2-7a

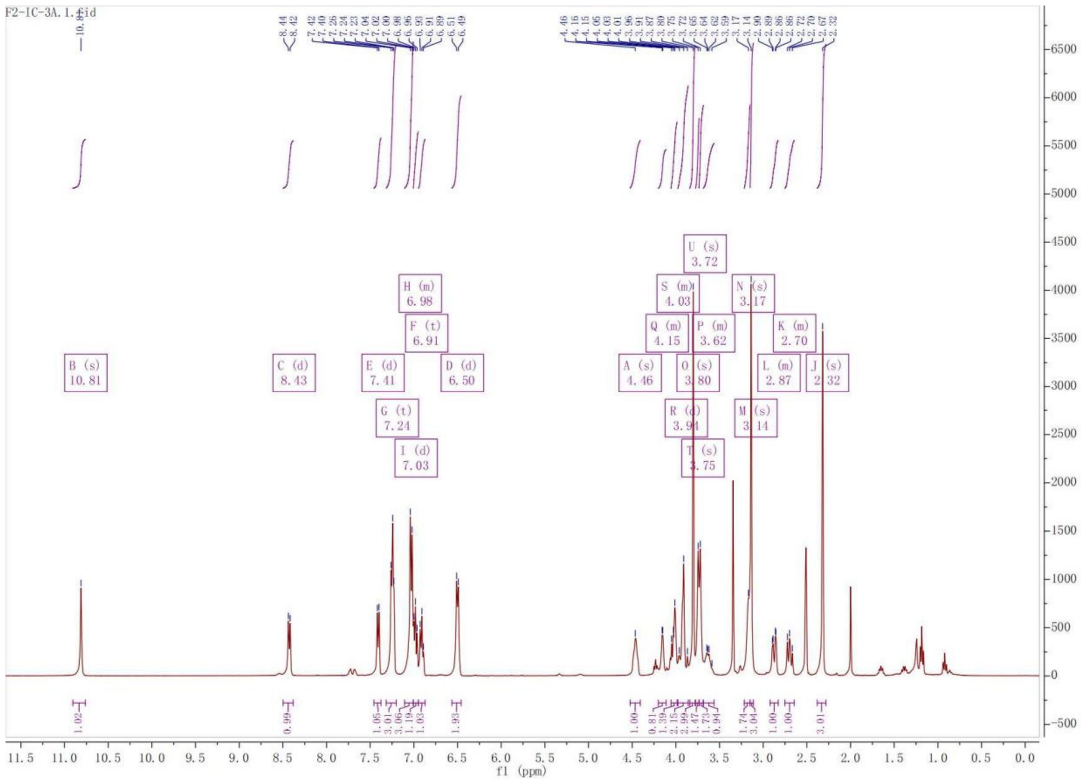
F2-IC-3A.1.fid
f1 (ppm)

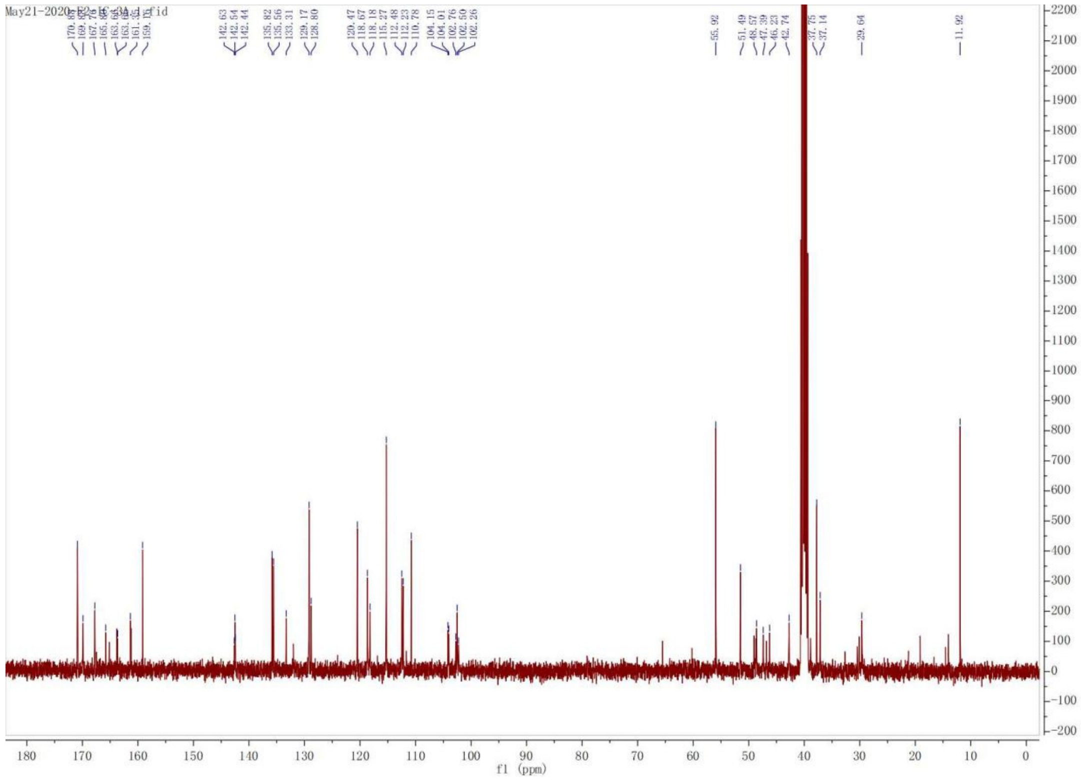
May21-2020
f1 (ppm)

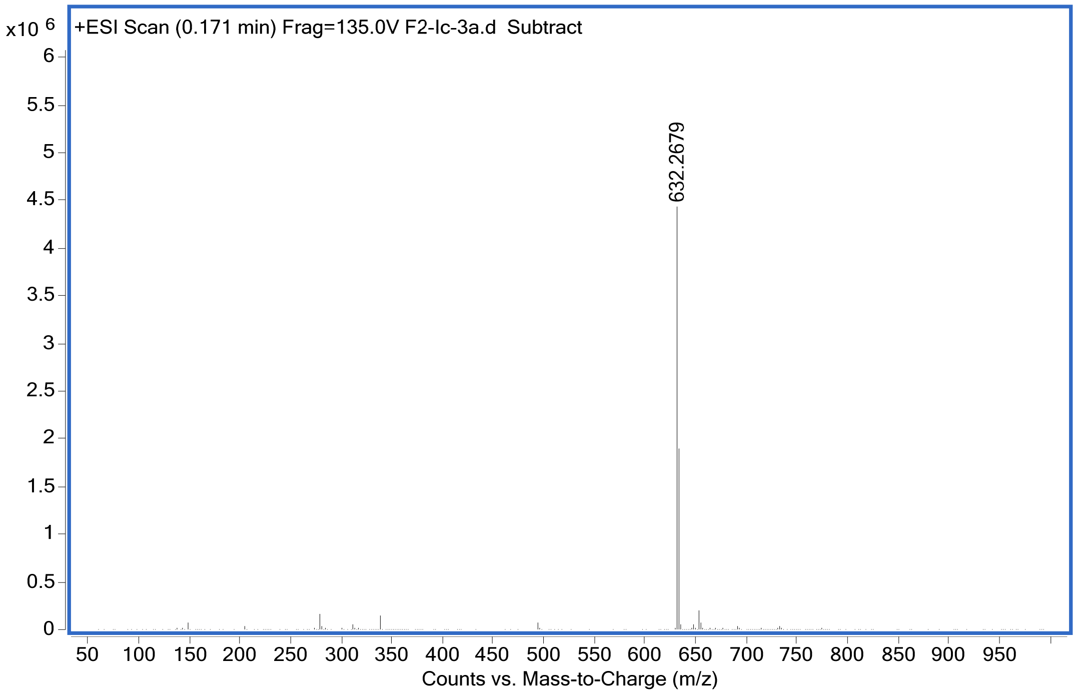

12. ^{1}H-NMR, ^{13}C-NMR and HRMS Spectra for F_2-7b

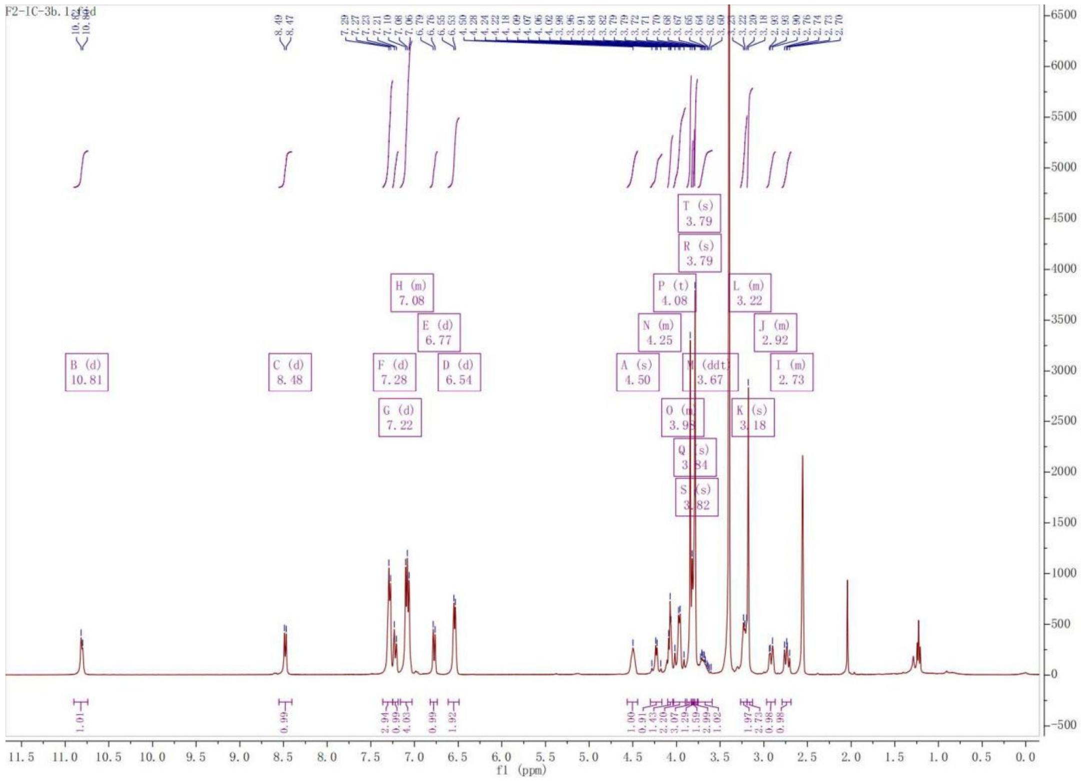

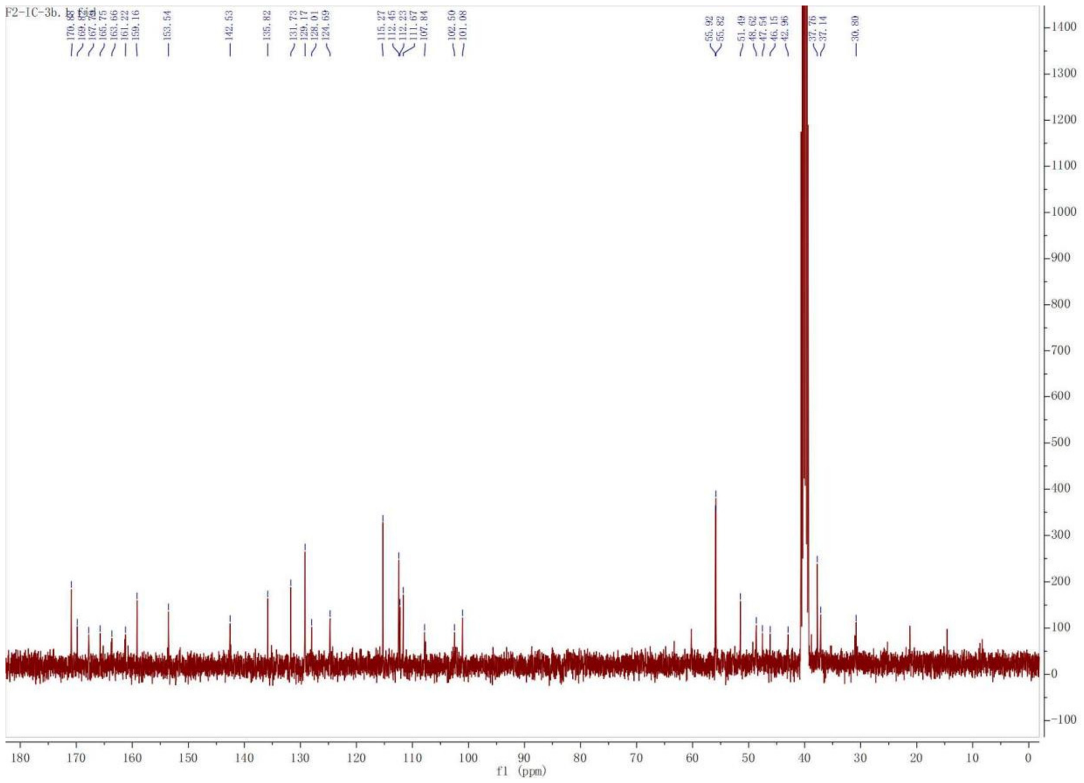

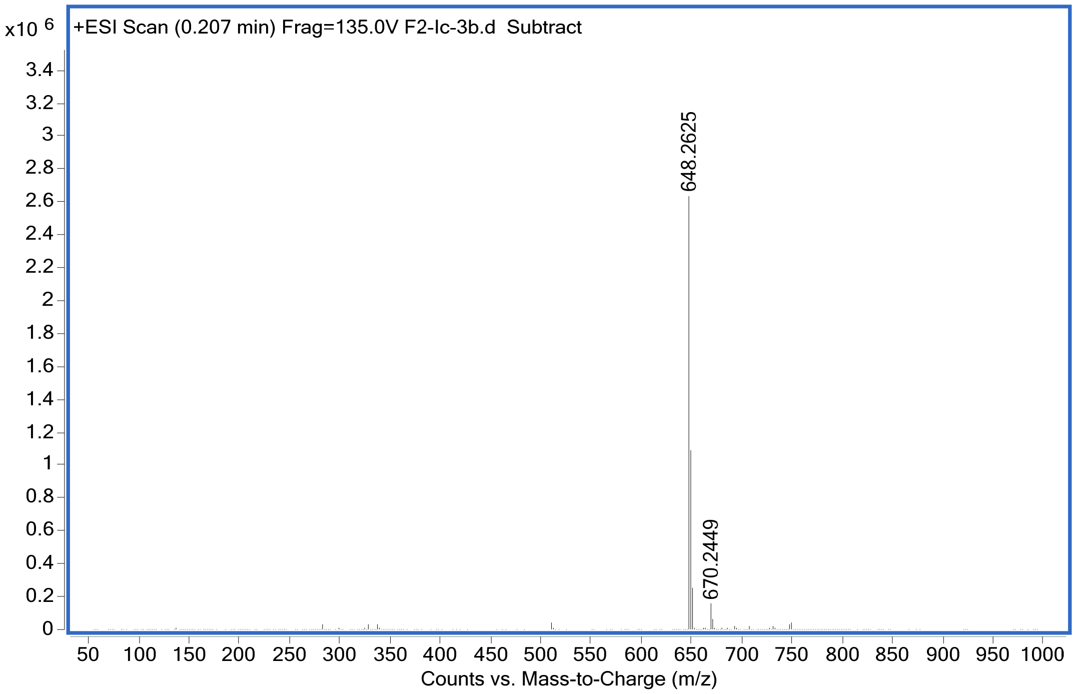

13.

14. ^{1}H-NMR, ^{13}C-NMR and HRMS Spectra for F_2-7c

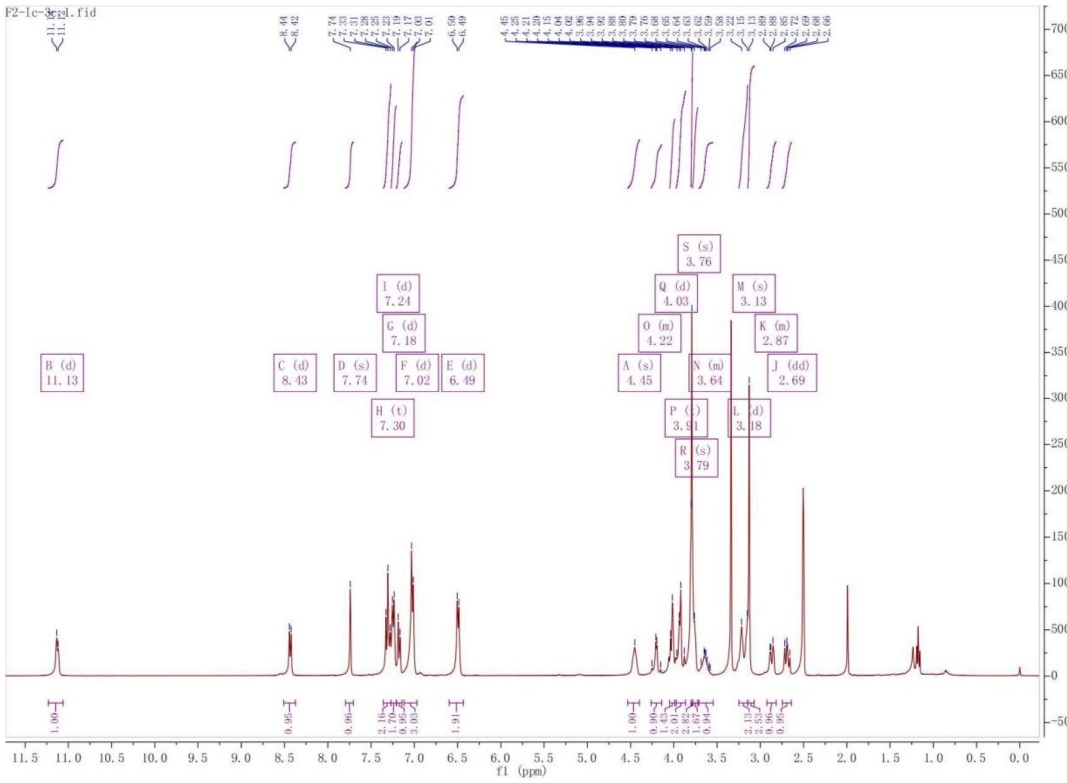
F2-Ic-3c-1.fid
f1 (ppm)

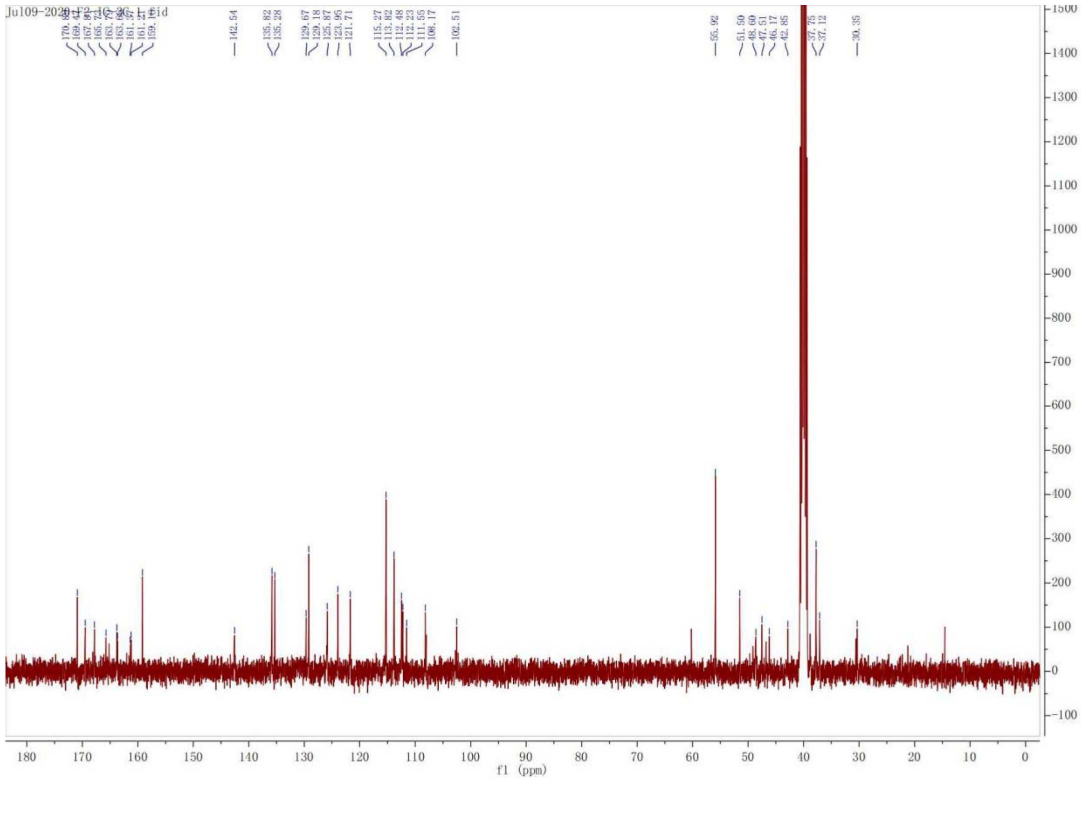
f1 (ppm)

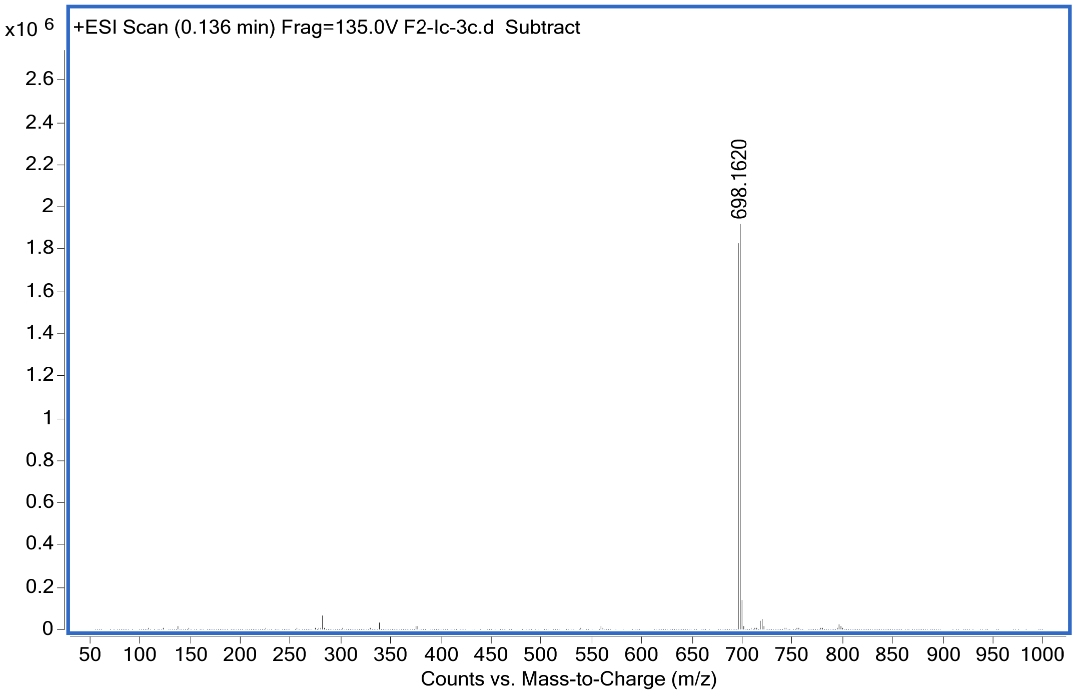

15.

16. ^{1}H-NMR, ^{13}C-NMR and HRMS Spectra for F_2-7d

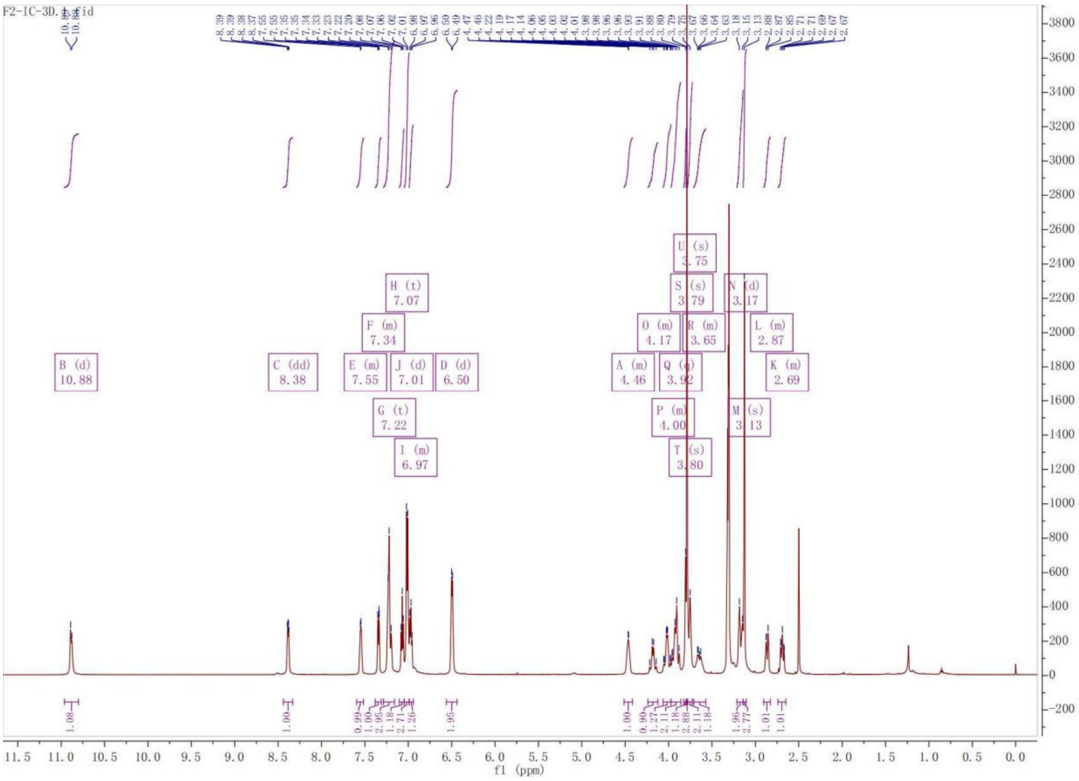

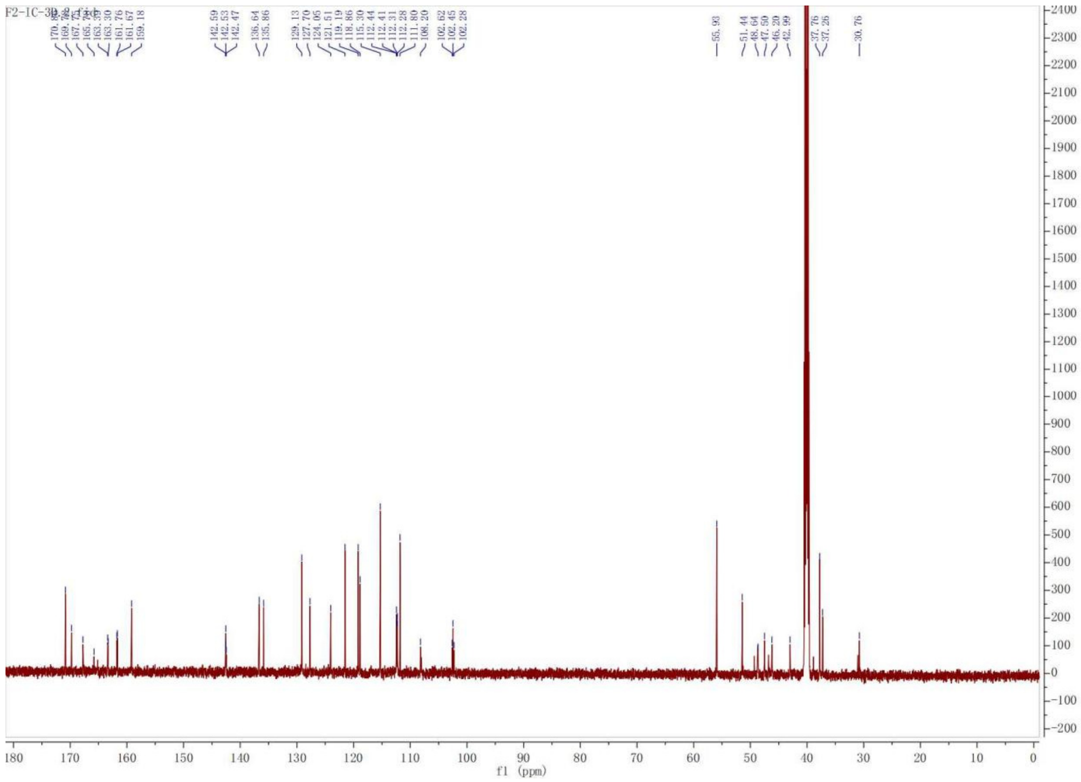

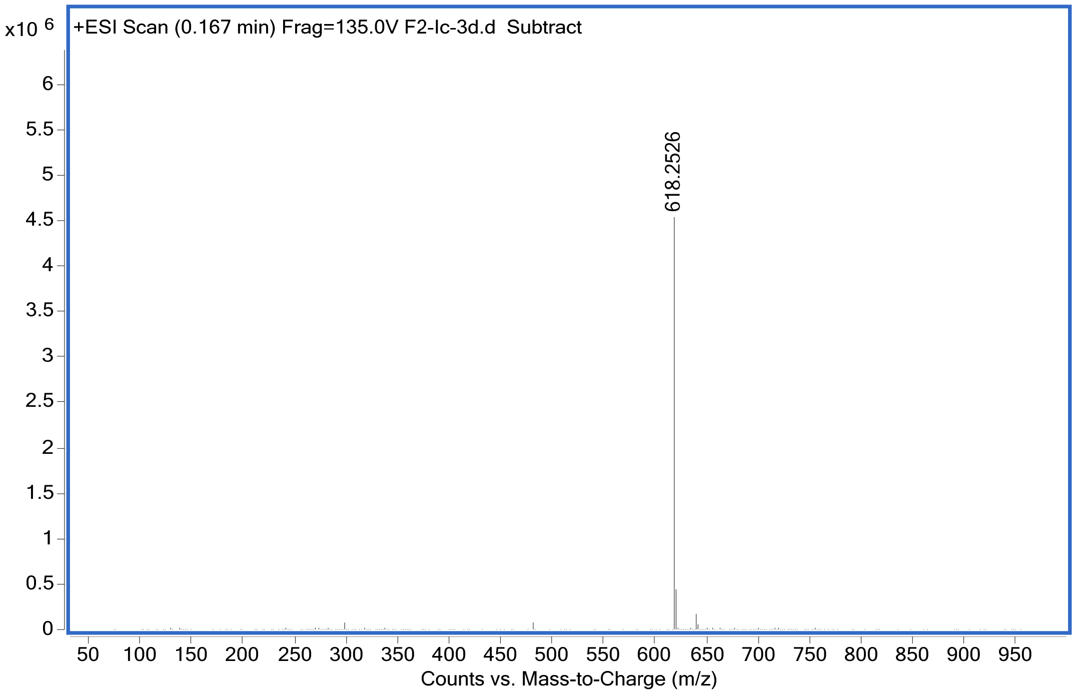

17.

18. ¹H-NMR, ¹³C-NMR and HRMS Spectra for F_2-7e

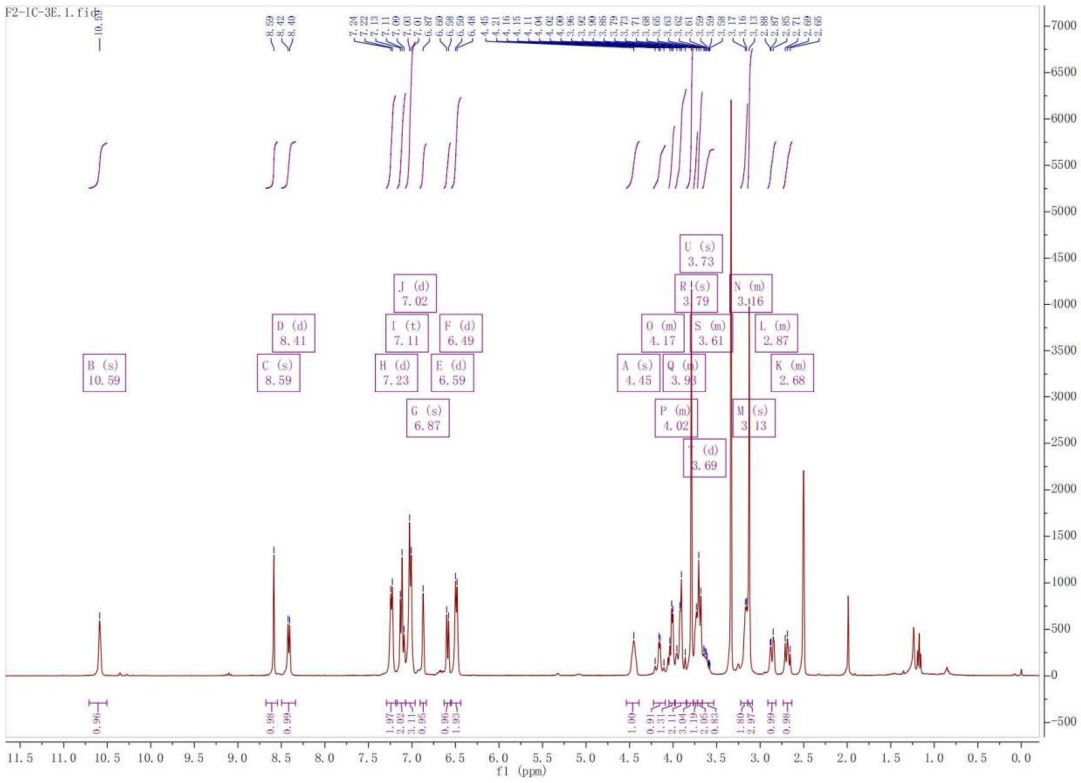
F2-IC-3E.1.fid
f1 (ppm)

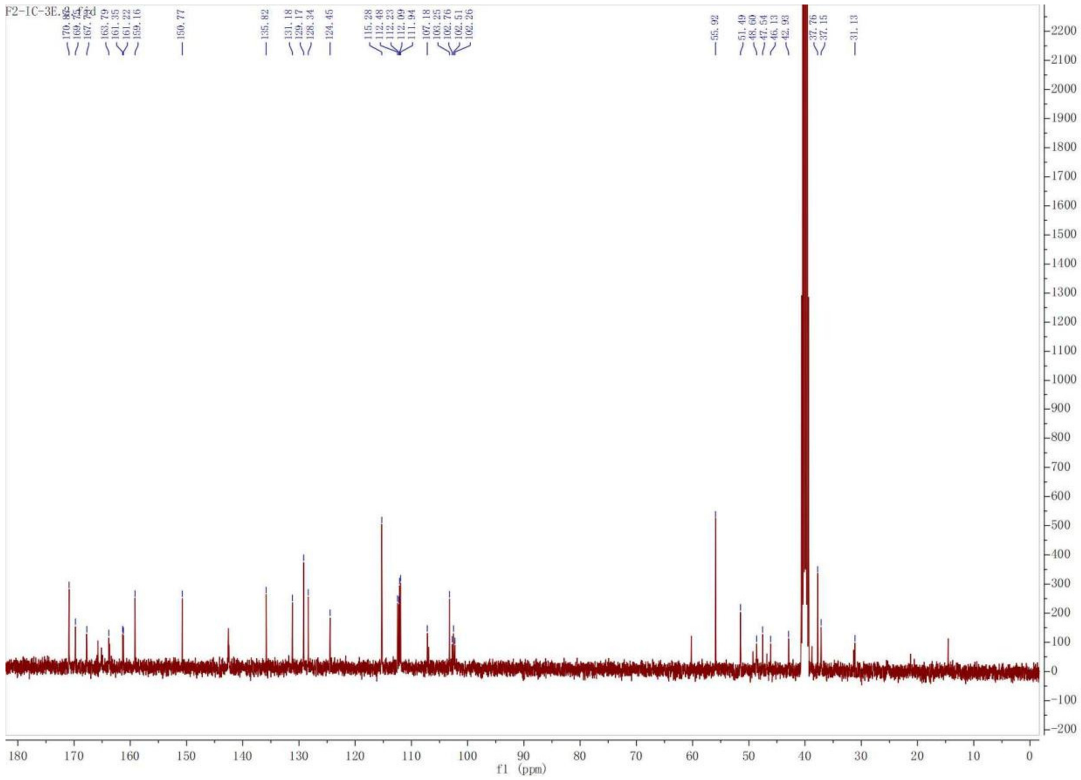
F2-IC-3E.2.fid
f1 (ppm)

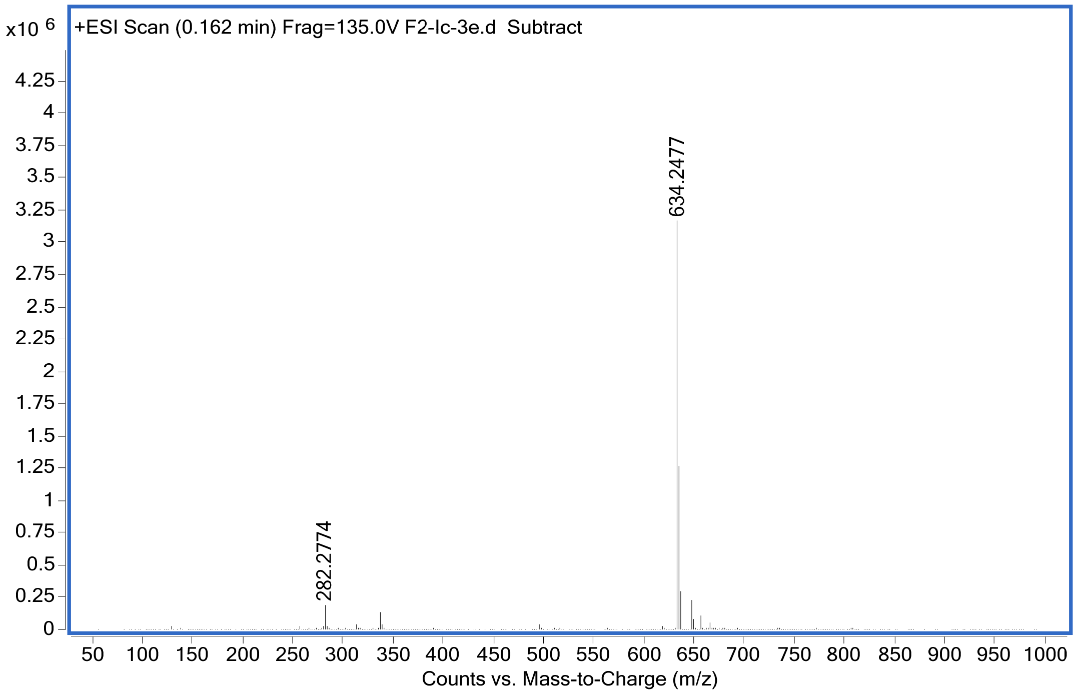

19. ^{1}H-NMR, ^{13}C-NMR and HRMS Spectra for F_2-7f

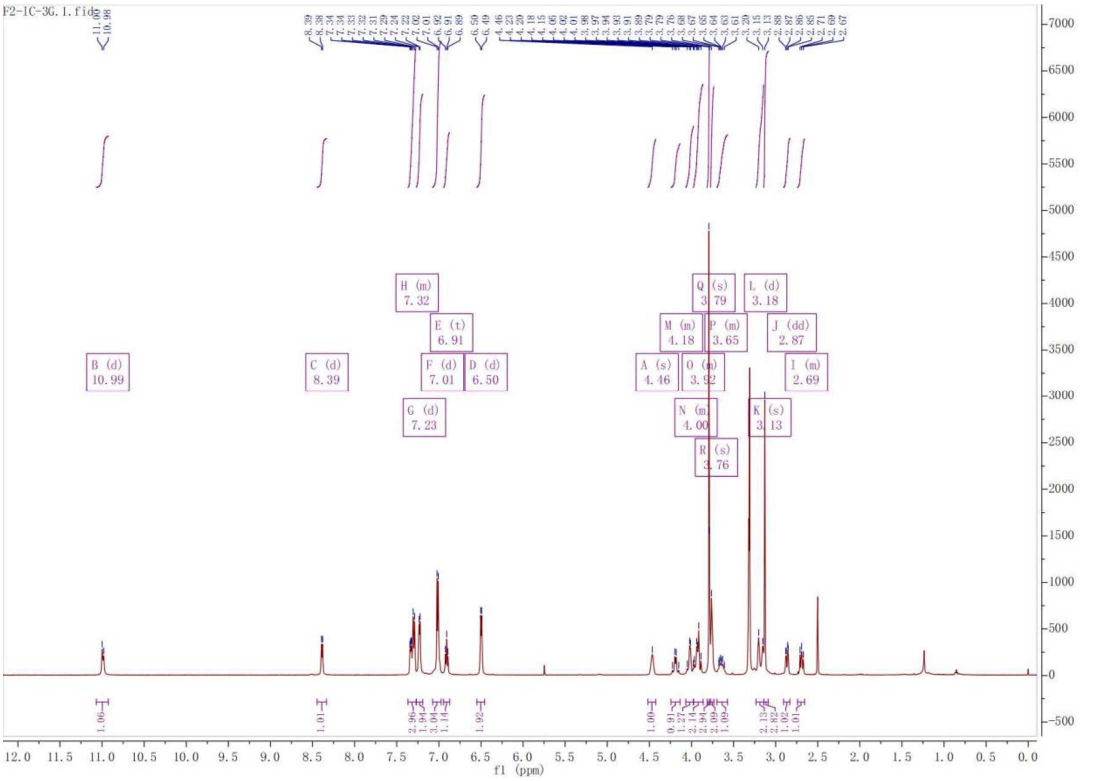

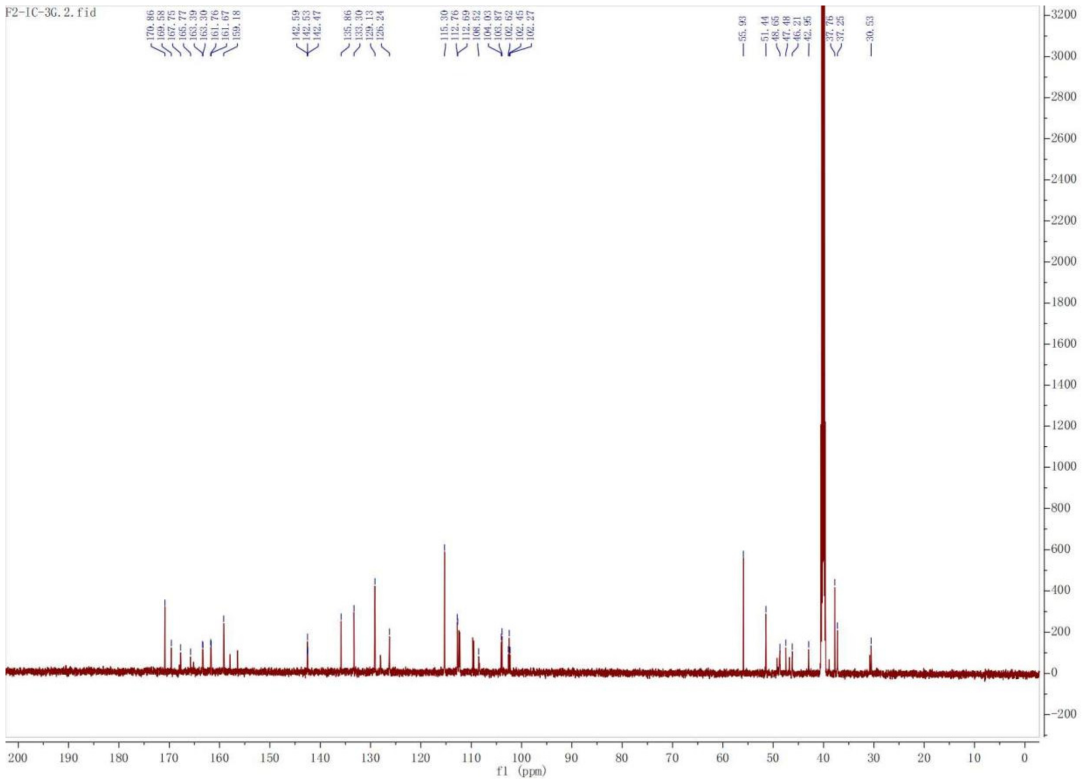

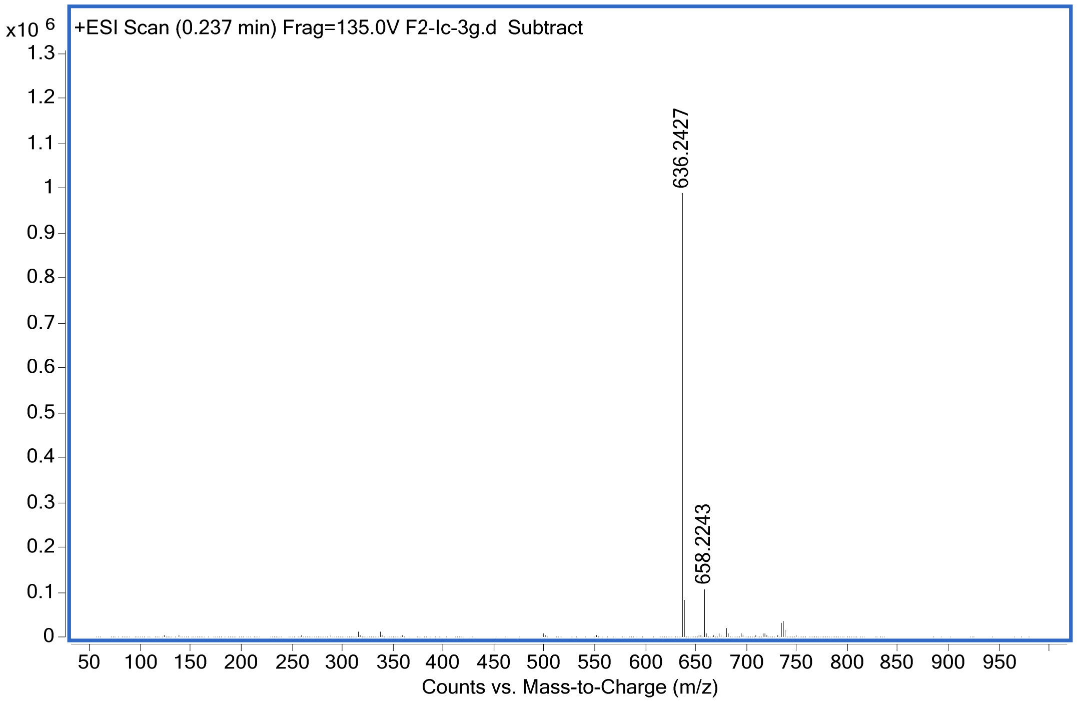

20. ^{1}H-NMR, ^{13}C-NMR and HRMS Spectra for 7g

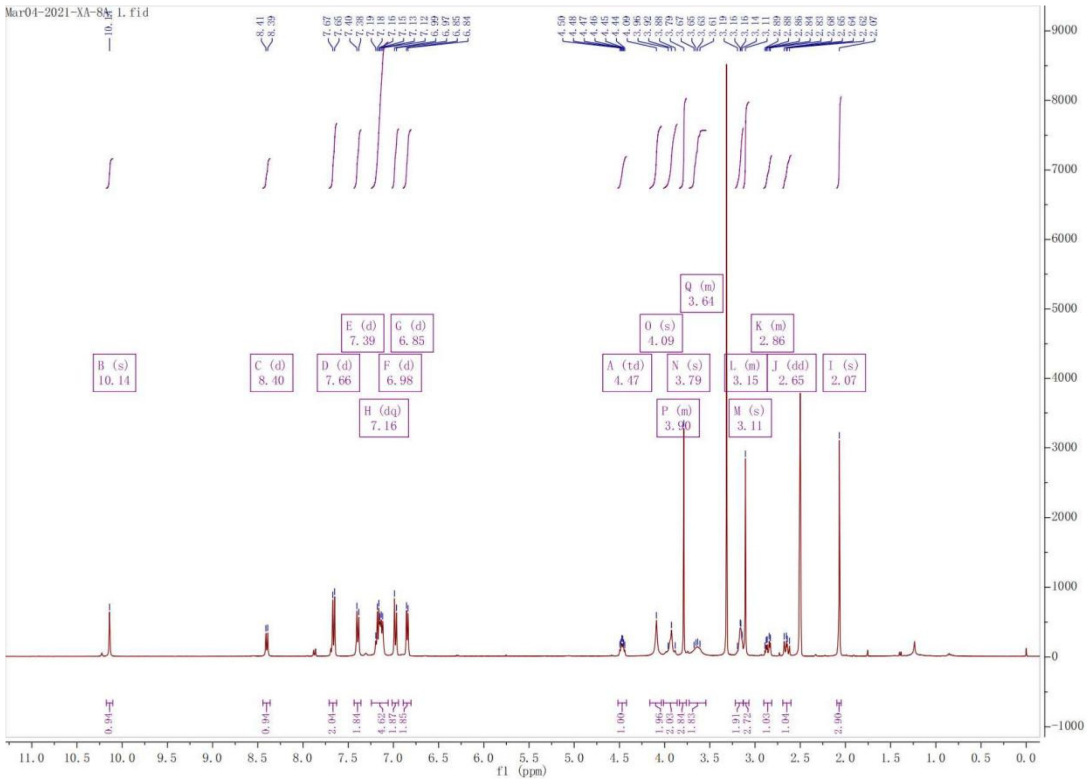

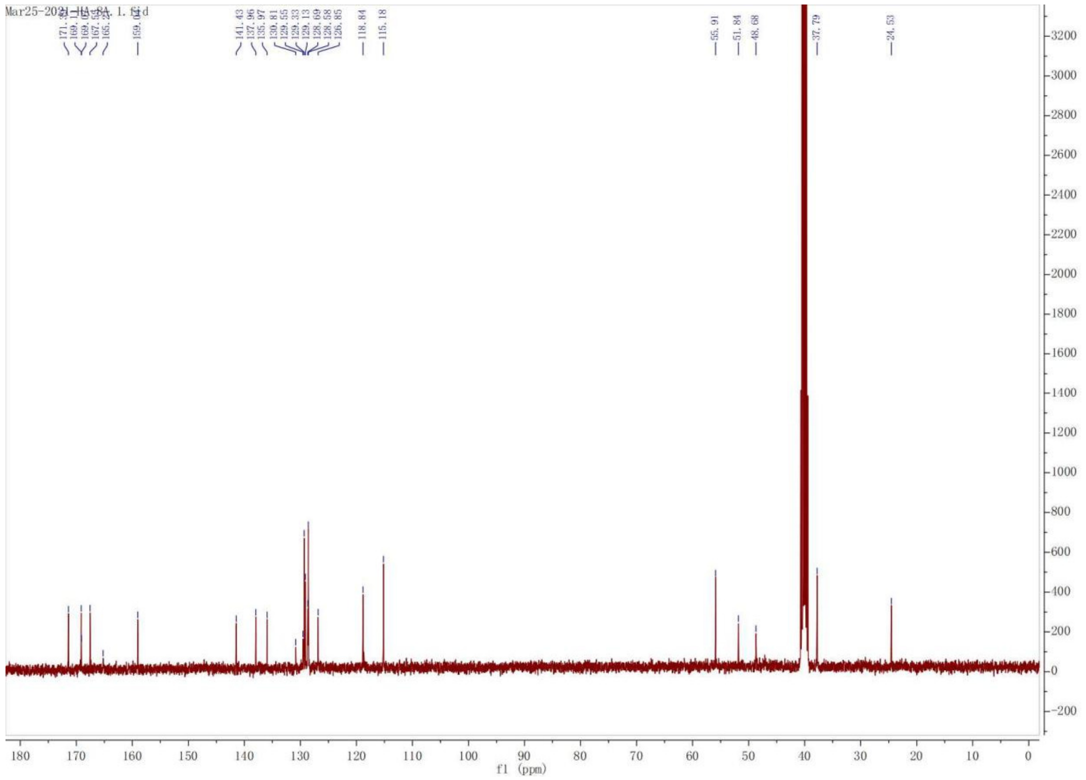

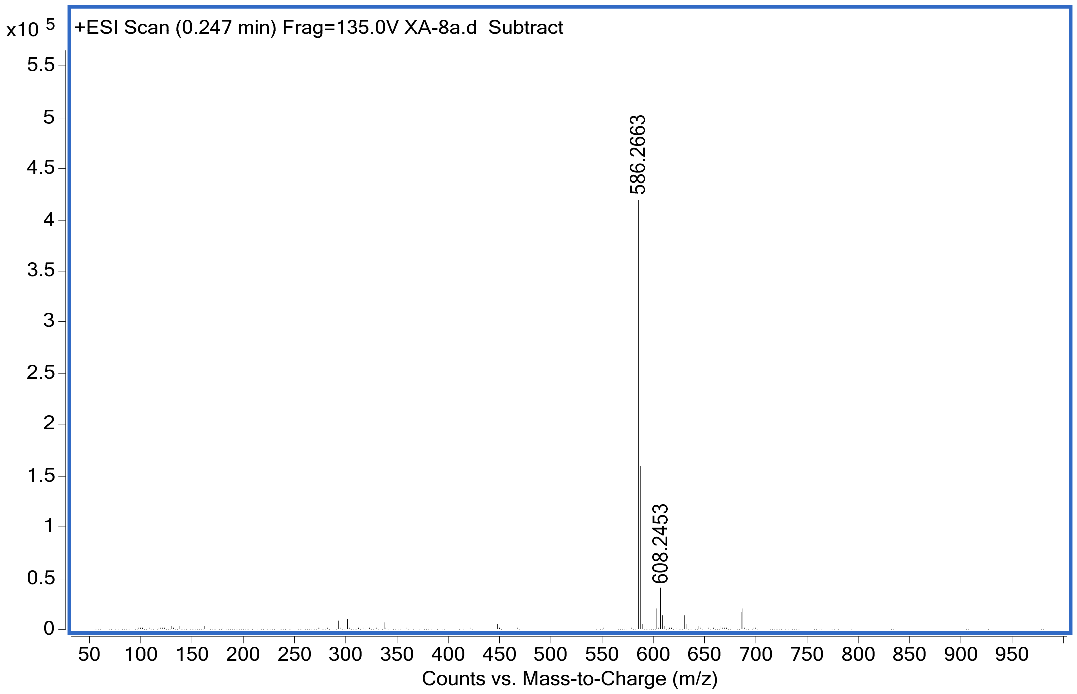

21. ^{1}H-NMR, ^{13}C-NMR and HRMS Spectra for 7h

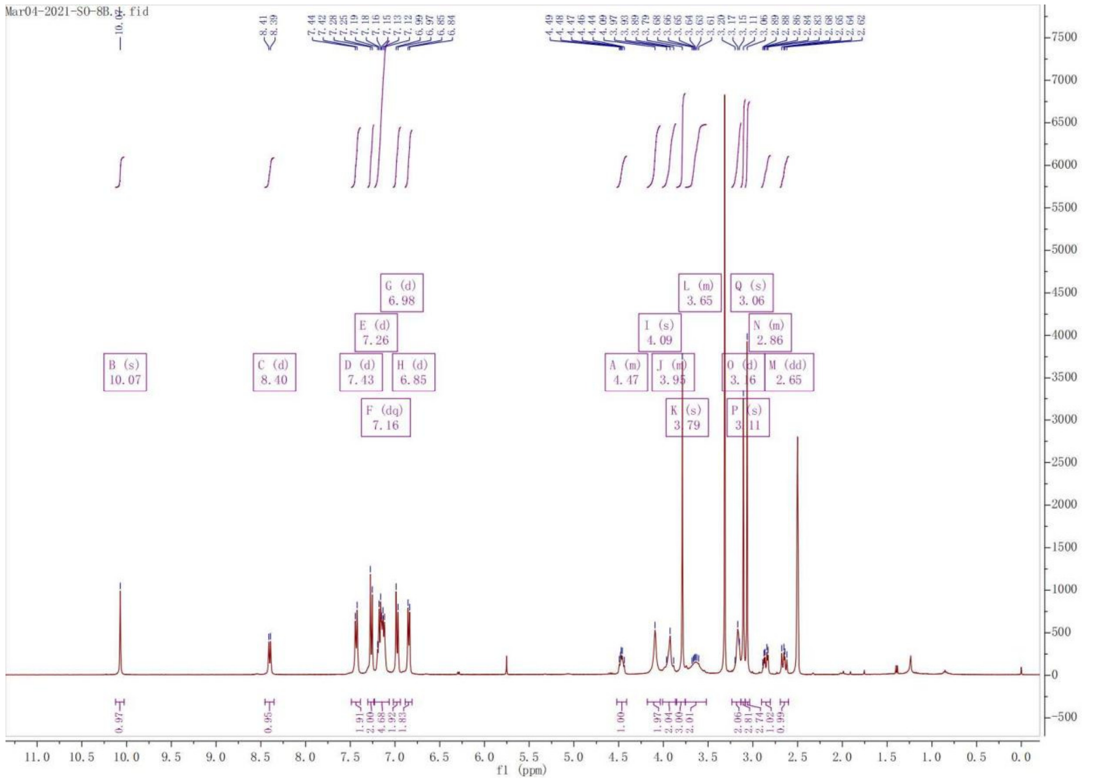

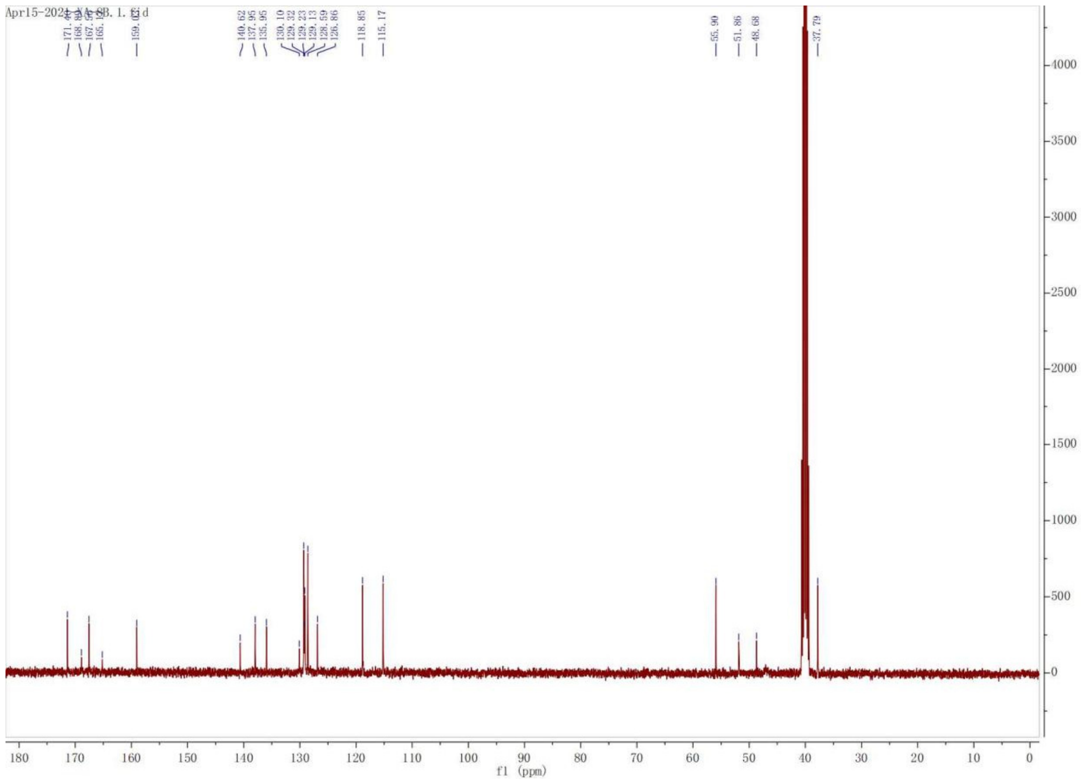

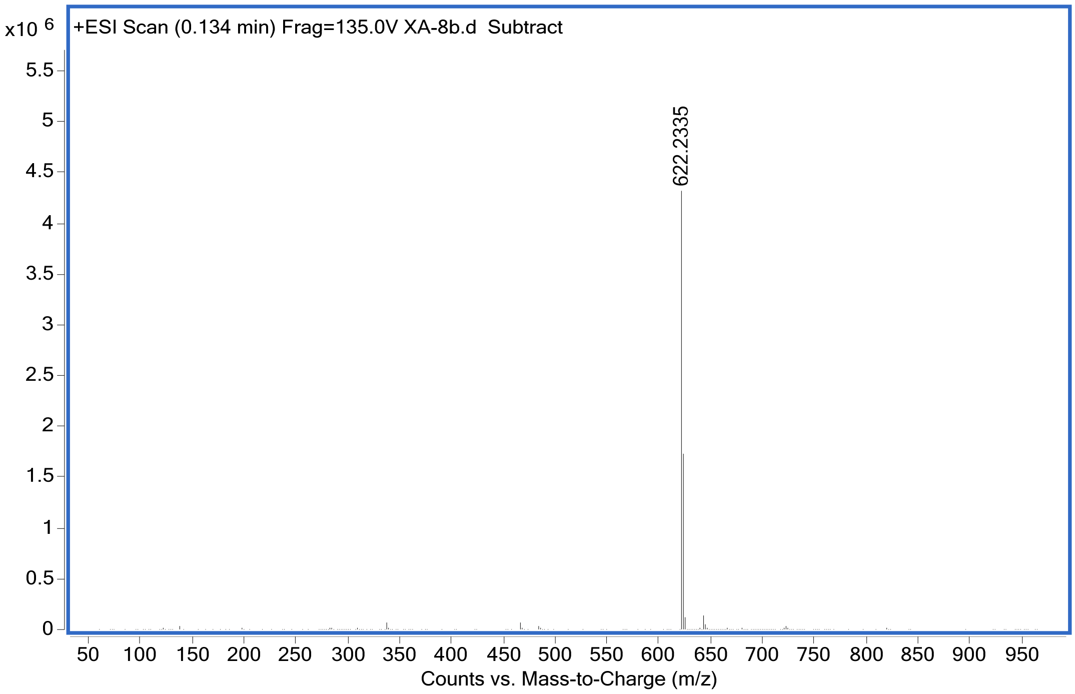

22.

23. ^{1}H-NMR, ^{13}C-NMR and HRMS Spectra for 7i

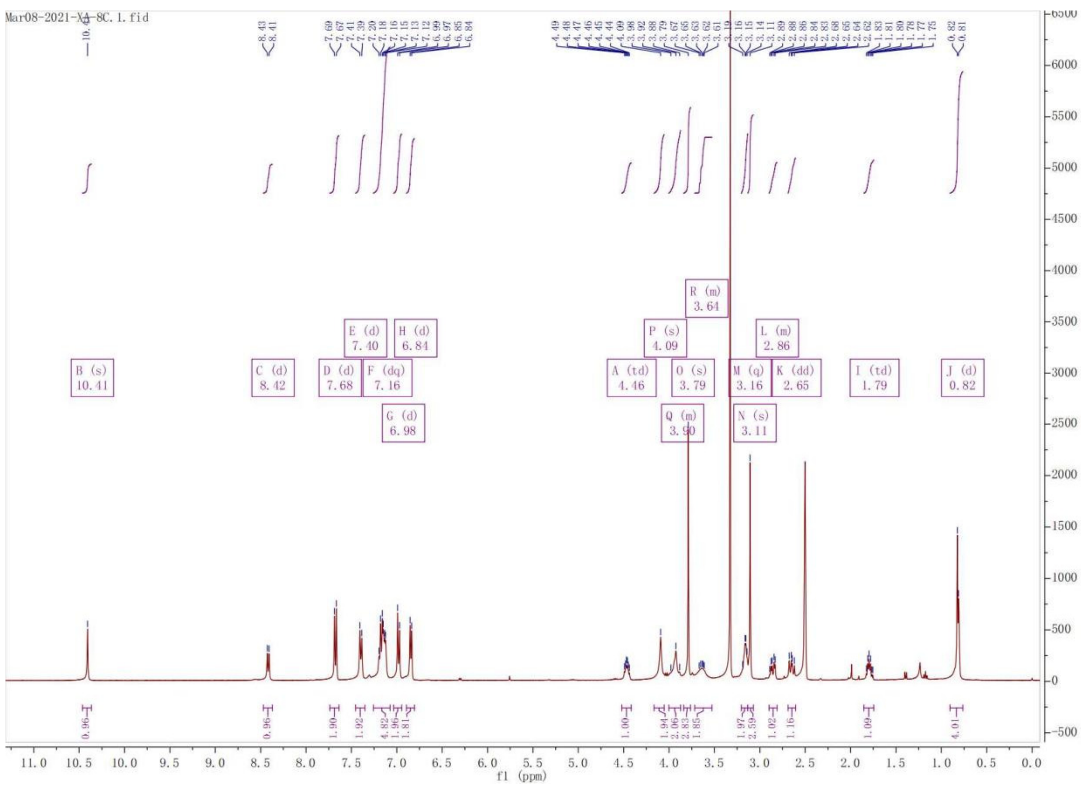

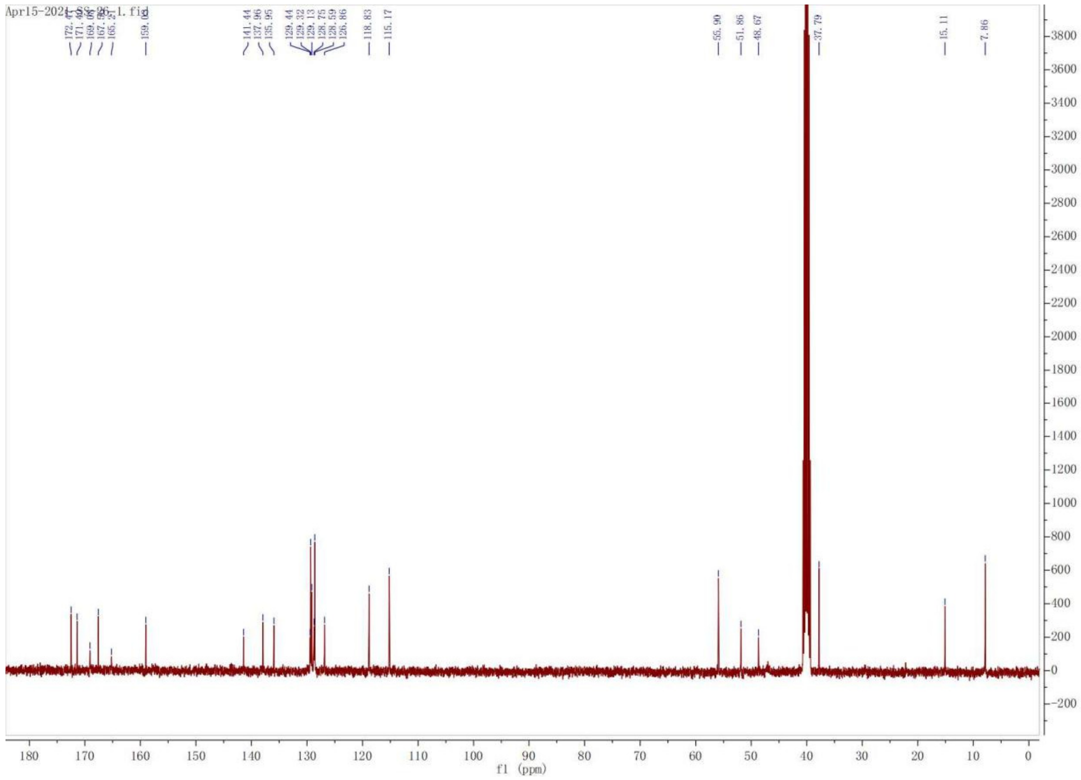

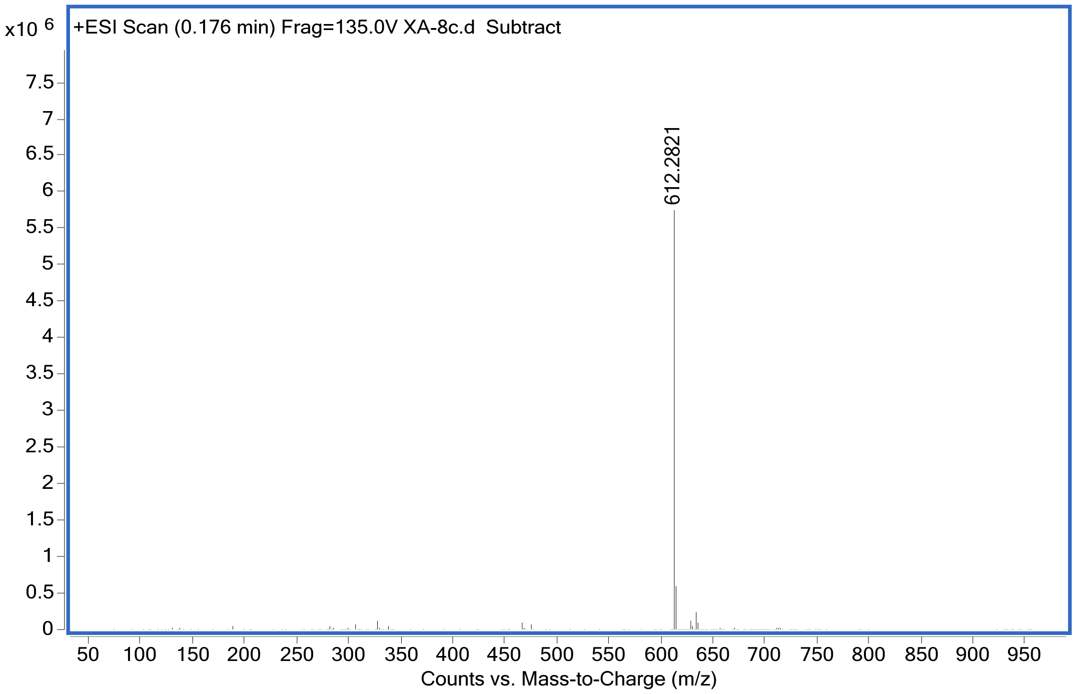

24.

25. ^{1}H-NMR, ^{13}C-NMR and HRMS Spectra for 7j

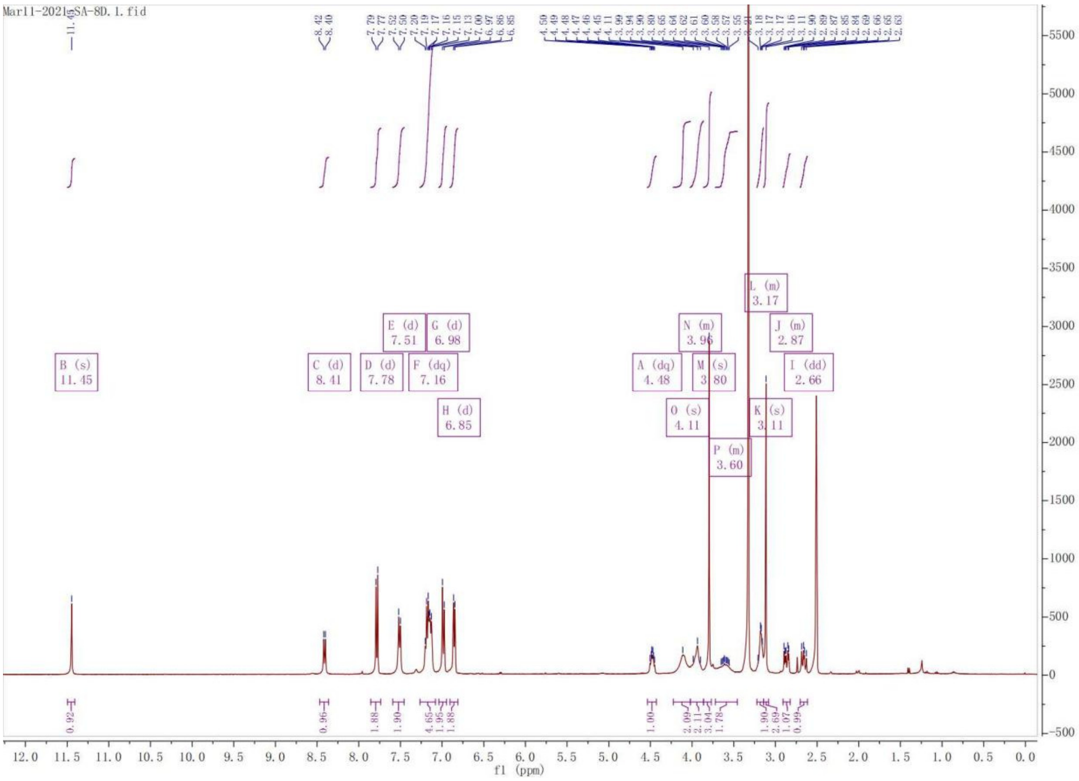

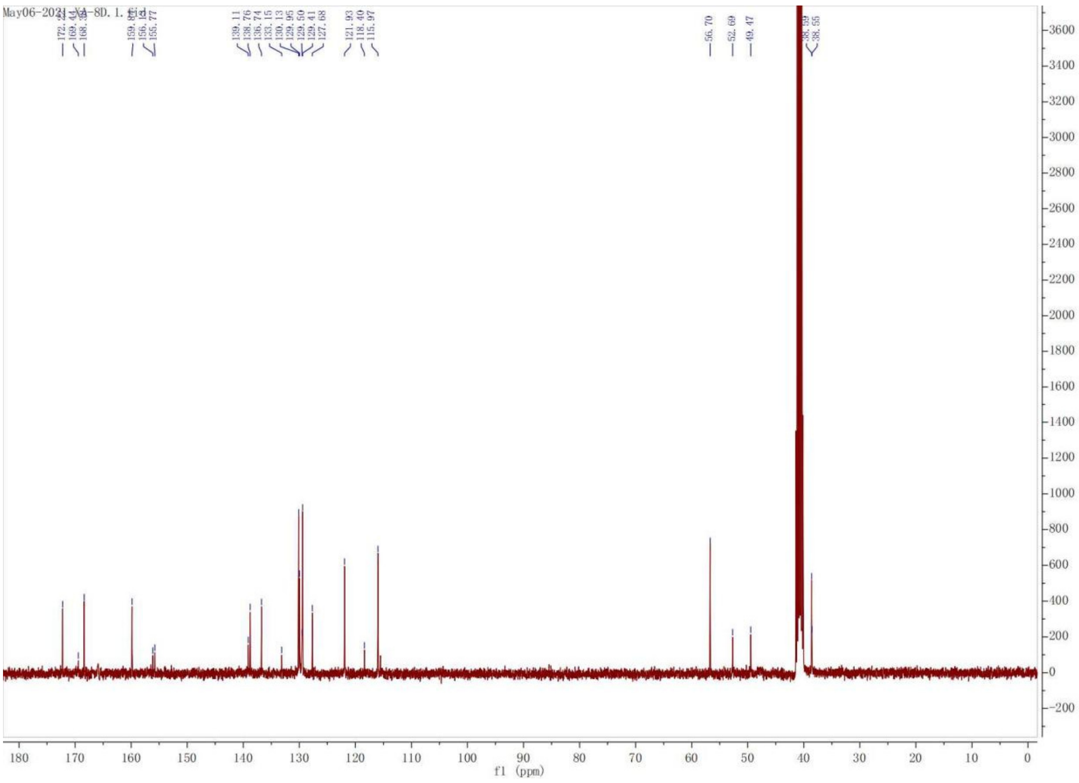

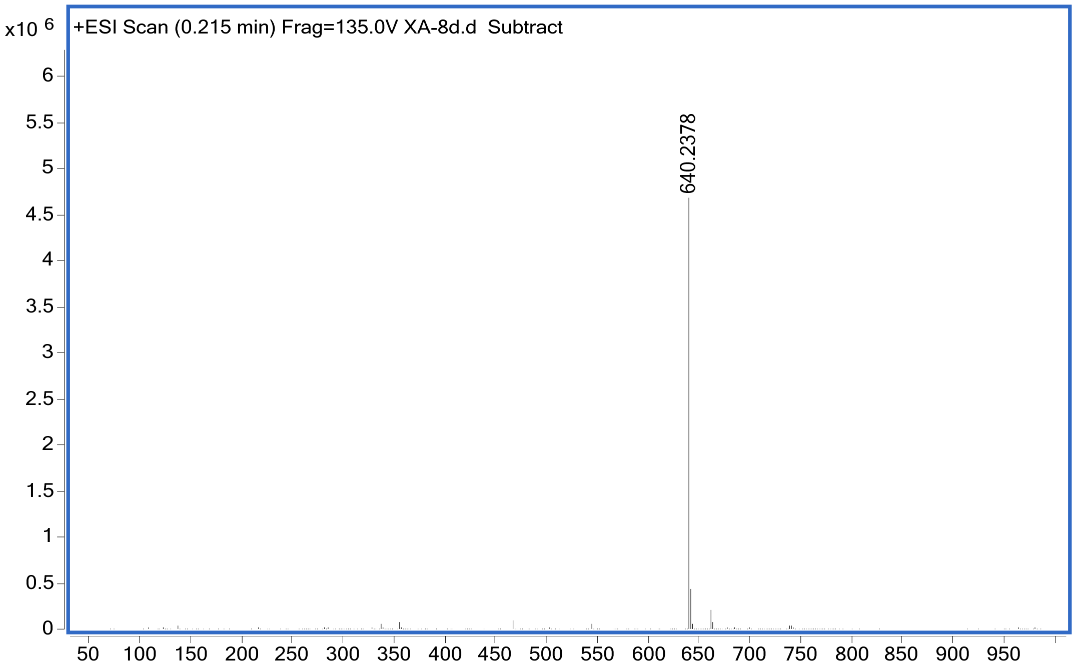

26.

27. ^{1}H-NMR, ^{13}C-NMR and HRMS Spectra for F_2-7g

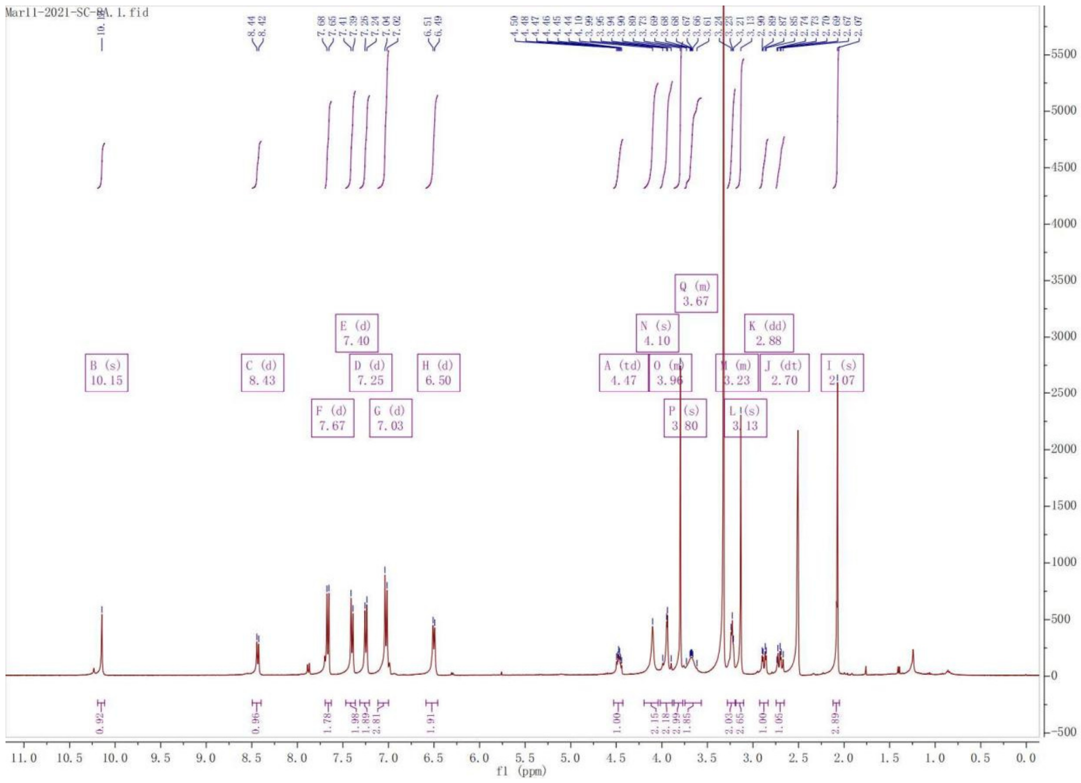

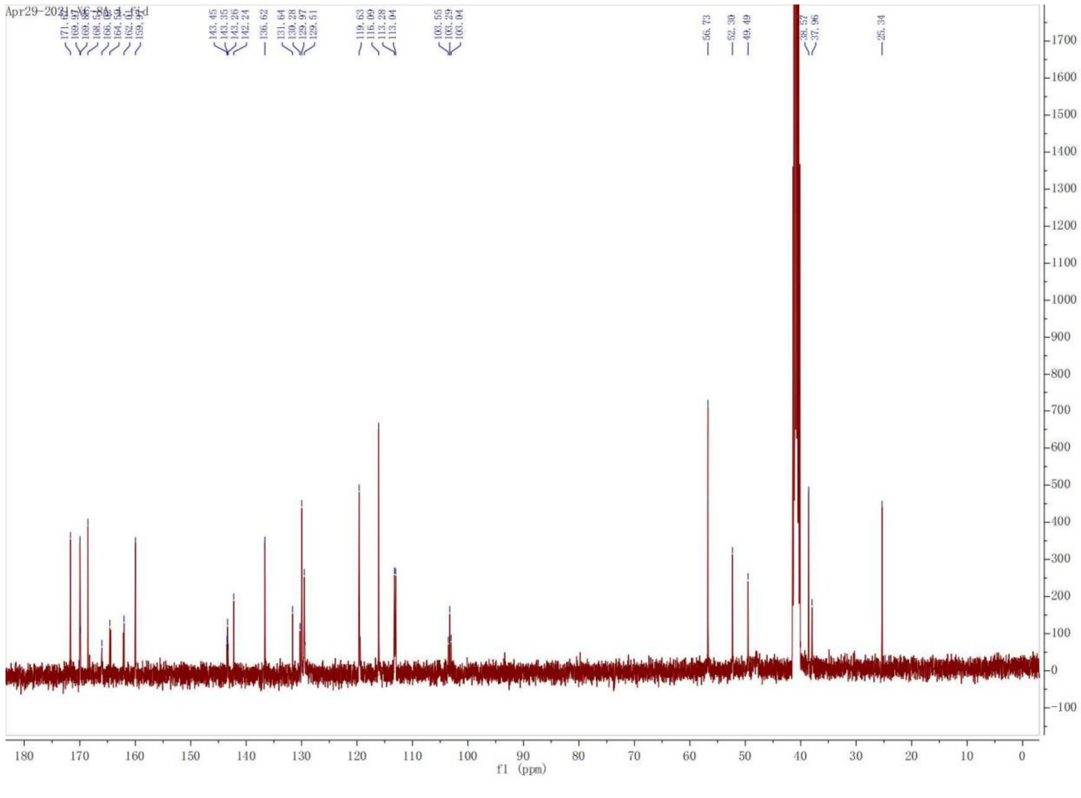

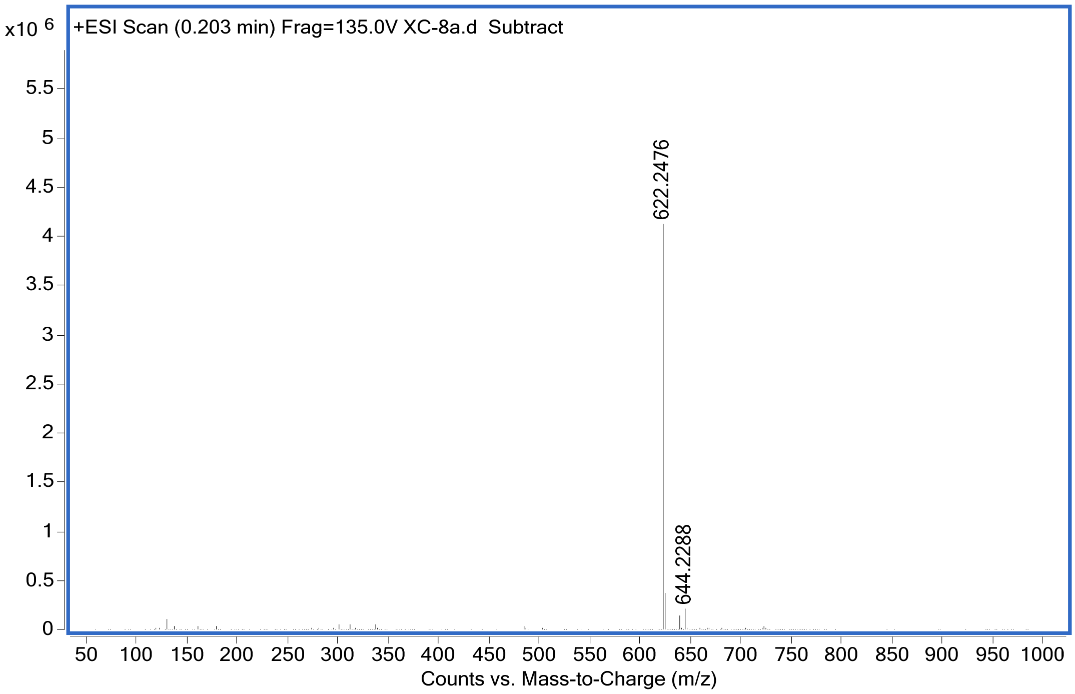

28.

29. ^{1}H-NMR, ^{13}C-NMR and HRMS Spectra for F_2-7h

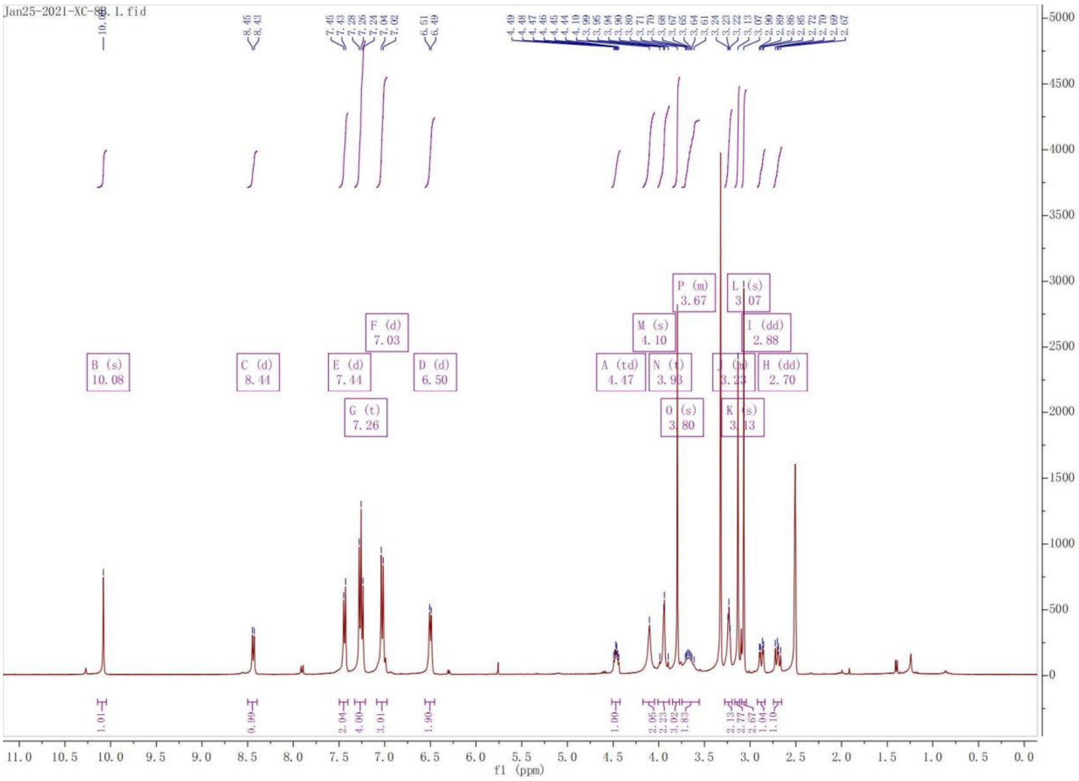

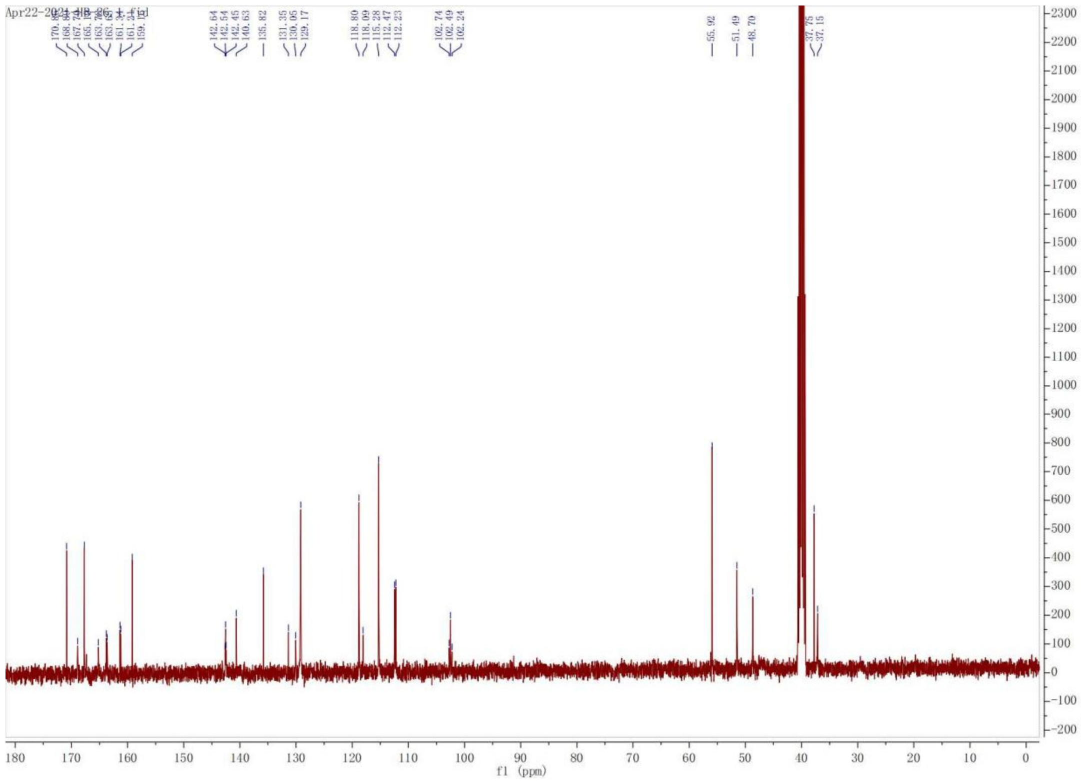

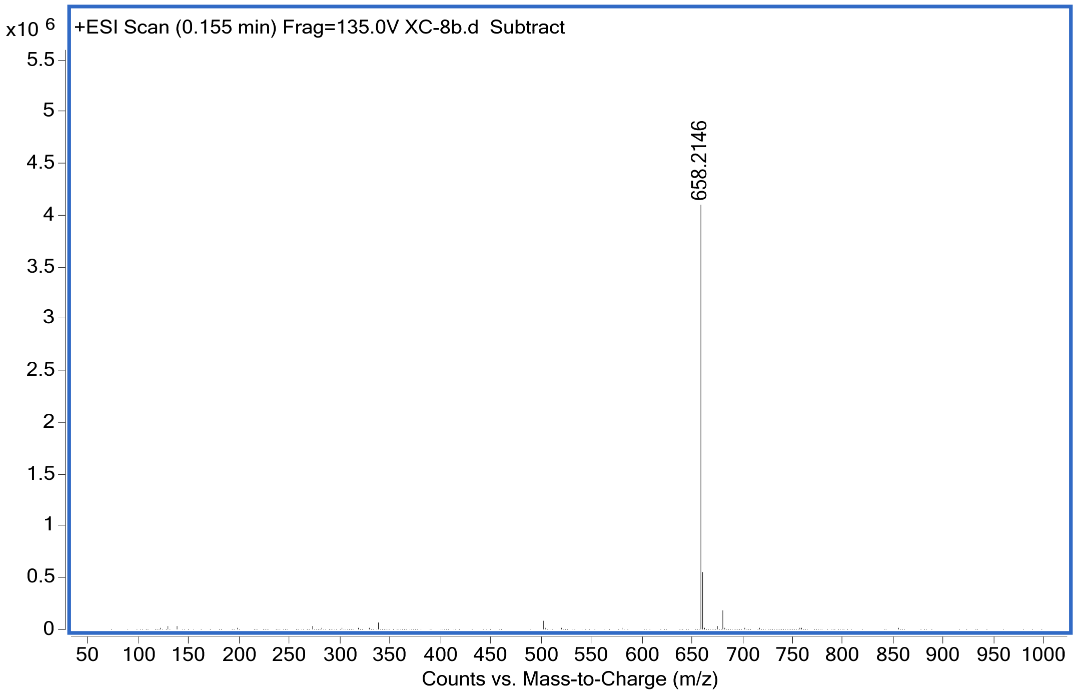

19. ^{1}H-NMR, ^{13}C-NMR and HRMS Spectra for F_2-7i

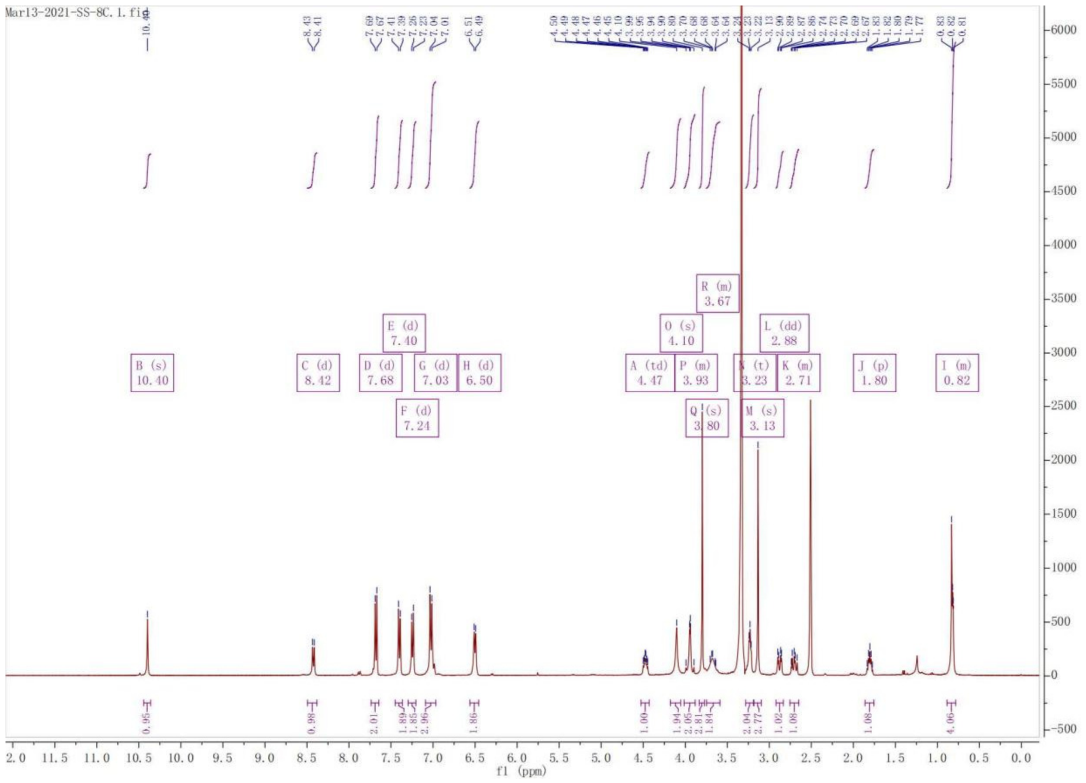

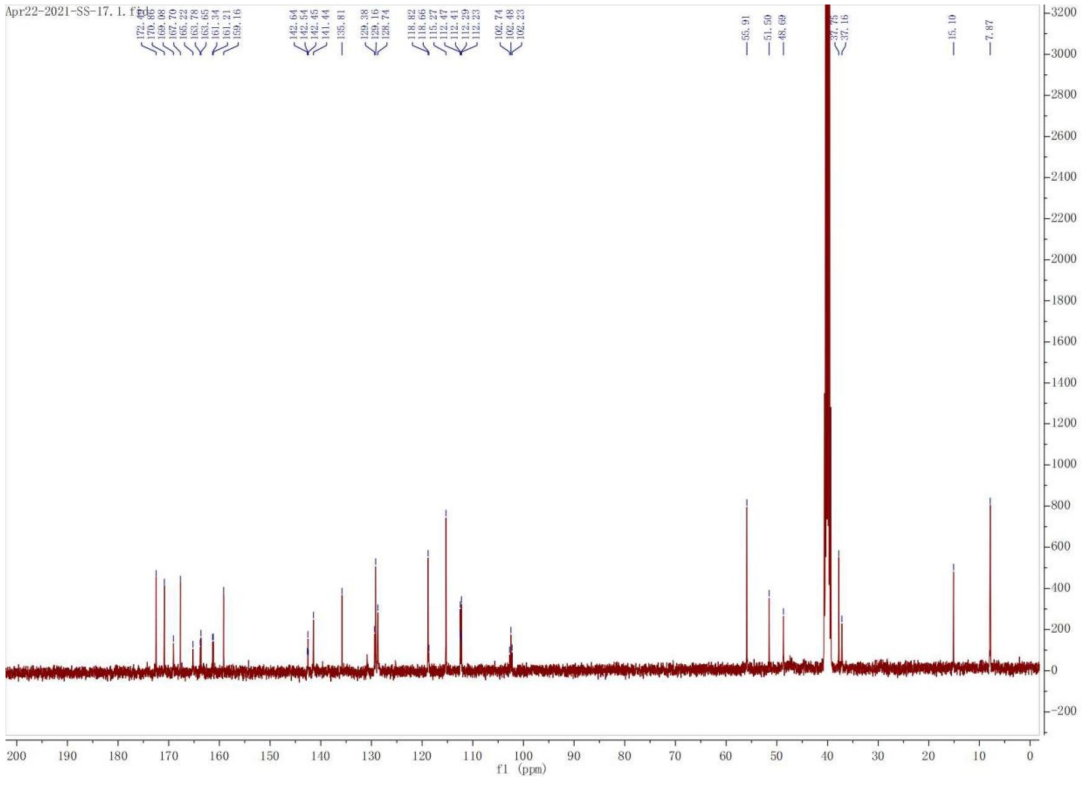

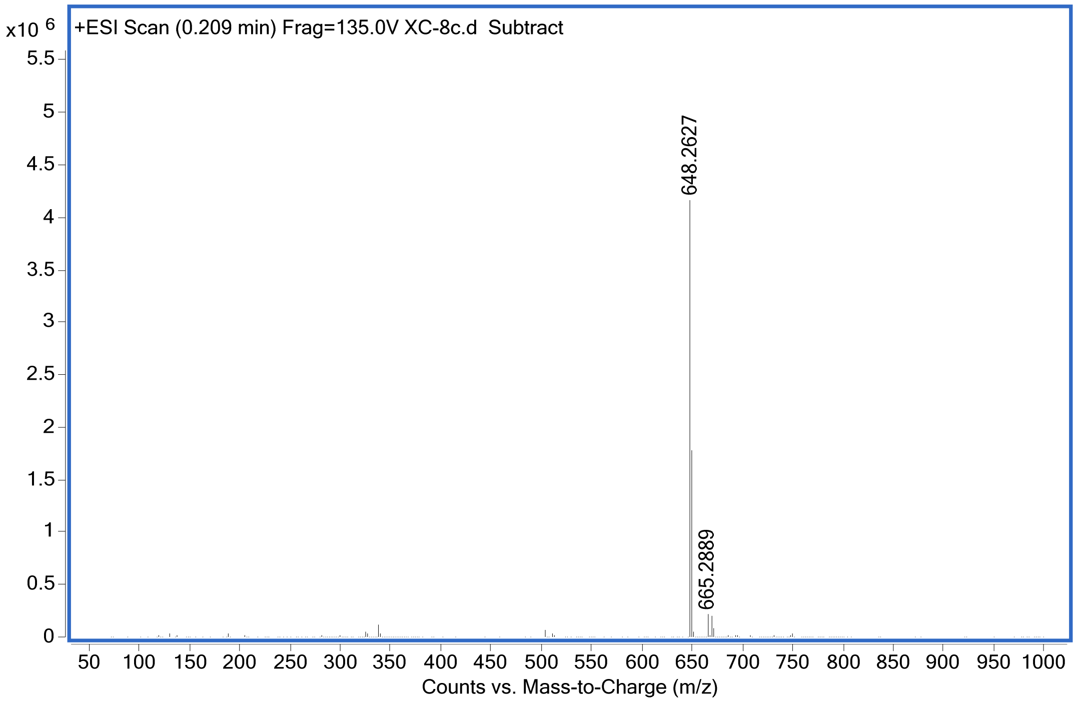

20. ^{1}H-NMR, ^{13}C-NMR and HRMS Spectra for F_2-7j

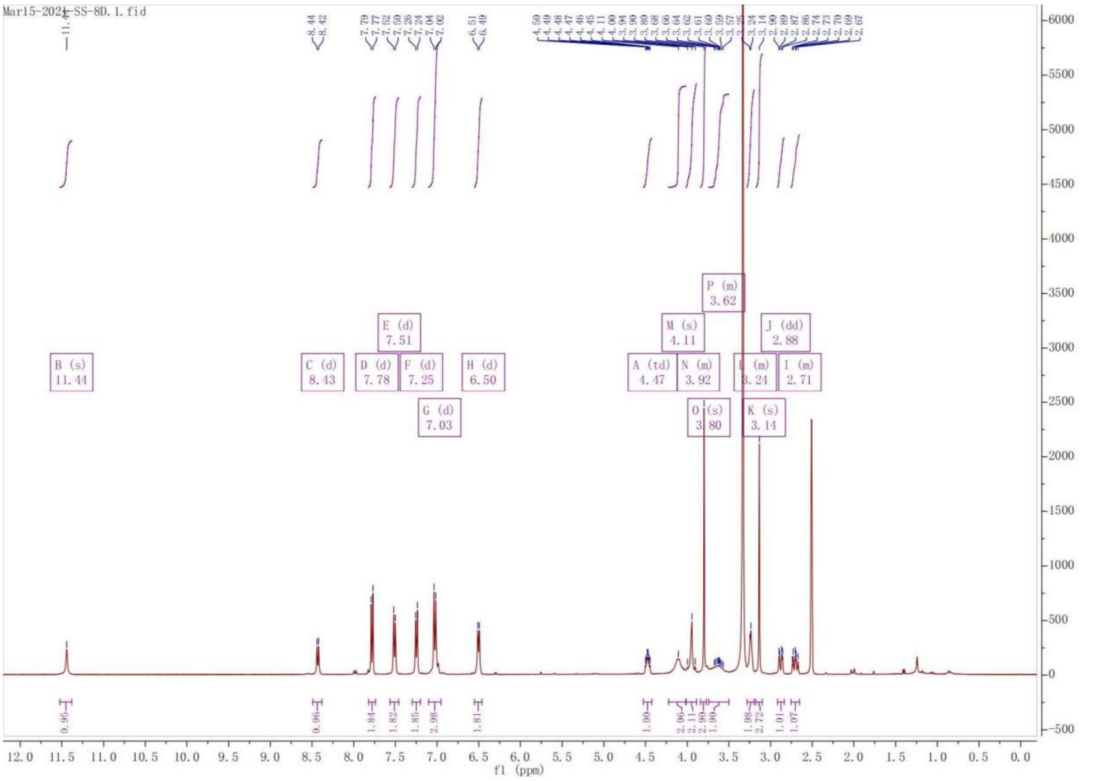

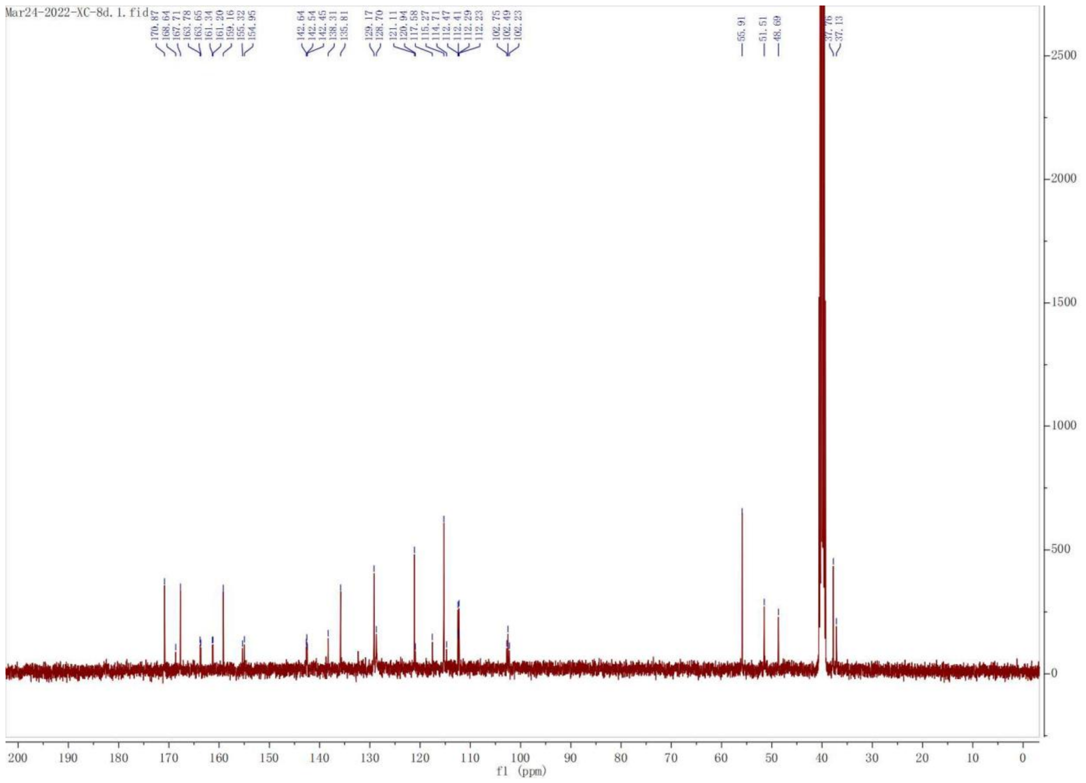

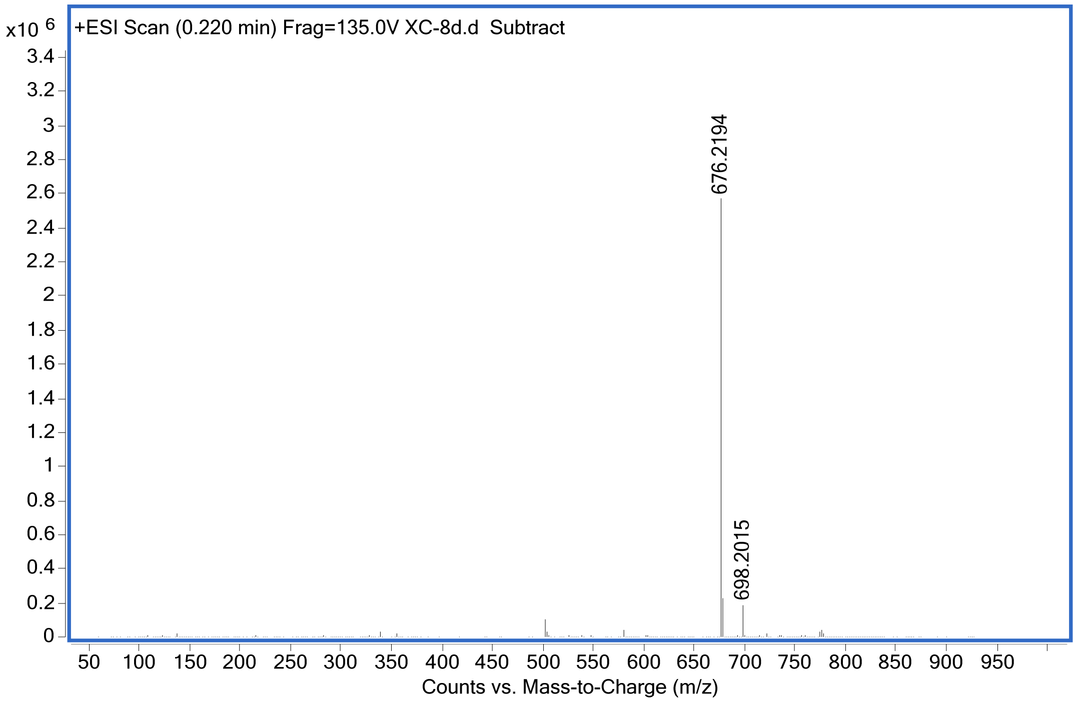

IX. References

1. Sun, L.; Dick, A.; Meuser, M.E.; Huang, T.; Zalloum, W.A.; Chen, C.H.; Cherukupalli, S.; Xu, S.; Ding, X.; Gao, P.; Kang, D.; De Clercq, E.; Pannecouque, C.; Cocklin, S.; Lee, K.H.; Liu, X.; Zhan, P. Design, Synthesis, and Mechanism Study of Benzenesulfonamide-Containing Phenylalanine Derivatives as Novel

HIV-1 Capsid Inhibitors with Improved Antiviral Activities. *J. Med. Chem.* **2020**, *63*, 4790-4810. https://doi.org/10.1021/acs.jmedchem.0c00015

2. Xu, S.; Sun, L.; Zalloum W. A.; Zhang, X.; Huang, T.; Ding, D.; Tao Y.; Zhao F.; Gao S.; Kang D.; De Clercq, E.; Pannecouque, C.; Dick, A.; Cocklin S.; Liu, X.; Zhan, P. From Design to Biological Mechanism Evaluation of Phenylalanine-bearing HIV-1 Capsid Inhibitors Targeting a Vital Assembly Interface. *Chin. Chem. Lett.* **2022**, https://doi.org/10.1016/j.cclet.2022.06.034.
3. Kortagere, S.; Madani, N.; Mankowski, M. K.; Schon, A.; Zentner, I.; Swaminathan, G.; Princiotto, A.; Anthony, K.; Oza, A.; Sierra, L. J.; Passic, S. R.; Wang, X.; Jones, D. M.; Stavale, E.; Krebs, F. C.; Martin-Garcia, J.; Freire, E.; Ptak, R. G.; Sodroski, J.; Cocklin, S.; Smith, A. B., 3rd, Inhibiting Early-stage Events in HIV-1 Replication by Small-molecule Targeting of the HIV-1 Capsid. *J. Virol.* **2012,** *86*, 8472-8481. https://doi.org/10.1128/JVI.05006-11
4. Studier, F. W., Protein Production by Auto-induction in High Density Shaking Cultures. *Protein Expr. Purif.* **2005,** *41*, 207-234. https://doi.org/10.1016/j.pep.2005.01.016
5. Jacques, D. A., McEwan, W. A., Hilditch, L., Price, A. J., Towers, G. J., & James, L. C. HIV-1 Uses Dynamic Capsid Pores to Import Nucleotides and Fuel Encapsidated DNA Synthesis. *Nature.* **2016**, *536*, 349. https://doi.org/10.1038/nature19098
6. Ester, M.; Kriegel, H.P.; Sander, J.; Xu, X. In Proceedings of 2nd International Conference on Knowledge Discovery and Data Mining; Simoudis, E., Han, J., Fayyad, U., Eds.; AAAI Press: Menlo Park, CA. **1996**, 226−231
7. OMEGA 3.0.0.1: OpenEye Scientific Software, Santa Fe, NM. http://www.eyesopen.com. Hawkins, P.C.D.; Skillman, A.G.; Warren, G.L.; Ellingson, B.A.; Stahl, M.T.
8. Hawkins, P. C.; Skillman, A. G.; Warren, G. L.; Ellingson, B. A.; Stahl, M. T. Conformer Generation with OMEGA: Algorithm and Validation Using High Quality Structures from the Protein Databank and the Cambridge Structural Database. *J. Chem. Inf. Model.* **2010**, *50*, 572-584.

https://doi.org/10.1021/ci100031x

9. OEDOCKING 3.0.1: OpenEye Scientific Software, Santa Fe, NM. http://www.eyesopen.com.
10. Kelley, B.P.; Brown, S.P.; Warren, G.L.; Muchmore, S.W. POSIT: Flexible Shape-Guided Docking For Pose Prediction. *J. Chem. Inf. Model.* **2015**, *55*, 1771-1780. https://doi.org/10.1021/acs.jcim.5b00142
11. McGann, M. FRED Pose Prediction and Virtual Screening Accuracy. *J. Chem. Inf. Model.* **2011**, *51*, 578-596. https://doi.org/10.1021/ci100436p
12. McGann, M. FRED and HYBRID Docking Performance on Standardized Datasets. *J. Comput. Aided Mol. Des.* **2012**, *26*, 897-906. https://doi.org/10.1007/s10822-012-9584-8
13. Case, D.A.; Betz, R.M.; Botello-Smith, W.; Cerutti, D.S.; Cheatham, T.E.; Darden, T.A.; Duke, R.E.; Giese, T.J.; Gohlke, H.; Goetz, A.W.; Homeyer, N.; Izadi, S.; Janowski, P.; Kaus, J.; Kovalenko, A.; Lee, T.S.; LeGrand, S.; Li, P.; Lin, C.; Luchko, T.; Luo, R.; Madej, B.; Mermelstein, D.; Merz, K.M.; Monard, G.; Nguyen, H.; Nguyen, H.T.; Omelyan I.; Onufriev, A.; Roe, D.R.; Roitberg, A.; Sagui, C.; Simmerling, C.L.; Swails, J.; Walker, R.C.; Wang, J.; Wolf, R.M.; Wu, X.; Xiao, L.; York D.M.; Kollman. P.A. AMBER 2016, University of California, San Francisco, **2016**.
14. Shao, J.; Tanner, S.W.; Thompson, N.; Cheatham, T.E. Clustering molecular Dynamics Trajectories: 1. Characterizing the Performance of Different Clustering Algorithms. *J. Chem. Theory. Comput.* **2007**, *3*, 2312−2334. https://doi.org/10.1021/ct700119m
15. Segall, M.; Champness, E.; Obrezanova, O.; Leeding, C., Beyond Profiling: Using ADMET Models to Guide Decisions. *Chem. Biodivers*. **2009**, *6*, 2144-2151. https://doi.org/10.1002/cbdv.200900148
16. Huey, R.; Morris, G.; Olson, A.J.; Goodsell, D.S. A Semiempirical Free Energy Force Field with Charge-based Desolvation. *J. Comput. Chem*. **2007**, *28*, 1145–1152. https://doi.org/10.1002/jcc.20634

17. Morris, G.M.; Huey, R.; Lindstrom, W.; Sanner, M.F.; Belew, R.K.; Goodsell, D.S.; Olson, A.J. AutoDock4 and AutoDockTools4: Automated Docking with Selective Receptor Flexibility. *J. Comput. Chem*. **2009**, *30*, 2785–2791. https://doi.org/10.1002/jcc.21256

18. Morris, G.M.; Huey, R.; Olson, A.J. Using AutoDock for Ligand-receptor Docking. Curr. Protoc. *Bioinform*. **2008**, *24*, 8–14. https://doi.org/10.1002/0471250953.bi0814s24

19. Bikadi, Z.; Hazai, E. Application of the PM6 Semi-empirical Method to Modeling Proteins Enhances Docking Accuracy of AutoDock. *J. Cheminform*. **2009**, *1*, 15. https://doi.org/10.1186/1758-2946-1-15

20. Karadsheh, R.; Meuser, M. E.; Cocklin, S., Composition and Orientation of The Core Region of Novel HIV-1 Entry Inhibitors Influences Metabolic Stability. *Molecules*. **2020**, *25*, 1430. https://doi.org/10.3390/molecules25061430

21. Tyzack, J. D.; Hunt, P. A.; Segall, M. D., Predicting Regioselectivity and Lability of Cytochrome P450 Metabolism Using Quantum Mechanical Simulations. *J. Chem. Inf. Model.* **2016**, *56*, 2180-2193. https://doi.org/10.1021/acs.jcim.6b00233

22. Hunt, P. A.; Segall, M. D.; Tyzack, J. D., WhichP450: a Multi-class Categorical Model to Predict the Major Metabolising CYP450 Isoform for a Compound. J. Comput. *Aided Mol. Des*. **2018**, *32*, 537-546. https://doi.org/10.1007/s10822-018-0107-0
